# A systematic comparison of different composite measures (DAS 28, CDAI, SDAI, and Boolean approach) for determining treatment effects on low disease activity and remission in rheumatoid arthritis

**DOI:** 10.1186/s41927-022-00314-7

**Published:** 2022-12-09

**Authors:** Kirsten Janke, Corinna Kiefer, Natalie McGauran, Bernd Richter, Dietmar Krause, Beate Wieseler

**Affiliations:** 1 Drug Assessment Department, Institute for Quality and Efficiency in Health Care (IQWiG), Im Mediapark 8, 50670 Cologne, Germany; 2 Medical Biometry Department, Institute for Quality and Efficiency in Health Care (IQWiG), Cologne, Germany; 3 Communications Unit, Institute for Quality and Efficiency in Health Care (IQWiG), Cologne, Germany; 4 Cochrane Metabolic and Endocrine Disorders Group, Institute of General Practice, Medical Faculty of the Heinrich-Heine-University Düsseldorf, Düsseldorf, Germany; 5 Rheumatology Practice Gladbeck, Gladbeck, Germany

**Keywords:** Rheumatoid arthritis, DAS 28, SDAI, CDAI, Boolean approach, DMARDs, Biologics, JAK inhibitors

## Abstract

**Background:**

Some composite measures for determining the treatment effects of disease-modifying antirheumatic drugs on remission and low disease activity (LDA) in rheumatoid arthritis (RA) may produce misleading results if they include an acute phase reactant (APR). To inform the choice of appropriate measure, we performed a systematic comparison of treatment effects using different composite measures.

**Methods:**

We used data generated for a systematic review of biologics in RA conducted by the Institute for Quality and Efficiency in Health Care and data from systematic reviews of newer biologics and Janus kinase (JAK) inhibitors provided by sponsors. The studies included had been conducted up to 2020 and investigated comparisons of biologics with placebo and head-to-head comparisons of biologics. Treatment effects on LDA and remission in studies investigating biologics or JAK inhibitors in RA were compared among 4 composite measures: the disease activity score 28 (DAS 28), the simplified disease activity index (SDAI), the Boolean approach (remission only), and the clinical disease activity index (CDAI)—only the latter does not include an APR.

**Results:**

49 placebo-controlled studies included 9 different biologics; 48 studies (16,233 patients) investigated LDA and 49 (16,338 patients) investigated remission. 11 active-controlled studies (5996 patients) investigated both LDA and remission and included 5 different head-to-head comparisons of biologics and 5 different comparisons (6 studies) of biologics with JAK inhibitors.

Statistically significantly larger treatment effects were found for biologics or JAK inhibitors versus placebo or active control in 16% of pairwise comparisons of composite measures (27 of 168). Most of these larger effects were observed for composite measures with an APR, i.e. the DAS 28 (19 comparisons) followed by the SDAI (*n* = 7). Larger effects were most frequently detected in favour of interleukin (IL)-6 inhibitors and to a lesser extent for JAK inhibitors versus treatments with different modes of action.

**Conclusions:**

The use of the DAS 28 and SDAI in clinical studies may generate results favouring certain treatments based on their mode of action (e.g. IL-6 inhibitors versus other biologics). To enable unbiased comparative effectiveness research, a composite measure without an APR (i.e. the CDAI) should thus be the measure of choice.

**Supplementary Information:**

The online version contains supplementary material available at 10.1186/s41927-022-00314-7.

## Background

Remission or at least low disease activity (LDA) is a major treatment outcome for patients with rheumatoid arthritis (RA) [1, 2]. Different composite measures with specific thresholds are available to measure the effects of treatment on these outcomes [2–6]. The most well-established one, the modified disease activity score including 28 joint counts (DAS 28 [4]), was developed in the 1990s and includes counts for swollen and tender joints, a patient global assessment, and an acute phase reactant (APR), either the C-reactive protein level or the erythrocyte sedimentation rate [7]. The composite score is calculated using a complex formula with weighting and/or transformation of the individual elements (Table 1). Besides being rather complex, a limitation of the DAS 28 is the use of a cut-off for remission of < 2.6, where patients may still have residual swollen joints and thus the risk of progression to joint damage and permanent functional disability [2, 8–10].

**Table 1:** Comparison of composite measures for assessment of remission and disease activity in rheumatoid arthritis*

Elements	DAS 28	SDAI	CDAI	Boolean approach (remission only)
Number of swollen joints (SJC)	0–28 (square root transformed)	0–28 (simple count)	0–28 (simple count)	≤ 1
Number of tender joints (TJC)	0–28 (square root transformed)	0–28 (simple count)	0–28 (simple count)	≤ 1
Patient global assessment (PtGA)	0–100 VAS in mm	0–10 scale	0–10 scale	≤ 1 (0–10 scale)
Physician global assessment (PhGA)	–	0–10 scale	0–10 scale	–
Acute phase reactant	ESR or CRP log transformed	CRP in mg/dl (0–10)	–	CRP ≤ 1 mg/dl
Total index	0–9.4 No immediate scoring, calculator required	0–86.0 No immediate scoring due to CRP, simple calculation	0–76.0 Immediate scoring, simple calculation	–
Formula	0.56 × √(28TJC) + 0.28 × √(28SJC) + 0.70 × ln(ESR) + 0.014 × PtGA or 0.56 × √(28TJC) + 0.28 × √(28SJC) + 0.36 × ln(CRP + 1) + 0.014 × PtGA + 0.96	28SJC + 28TJC + PhGA + PtGA + CRP	28SJC + 28TJC + PhGA + PtGA	–
*Cut-off*				
Remission	< 2.6	≤ 3.3	≤ 2.8	At any time point, patient must satisfy the above cut-offs
Low disease activity	< 3.2	≤ 11.0	≤ 10.0	–

*Columns 1–4 adapted from Tables 1 and 2 in [40], Column 5 from [2], Formulas added from [41]

Further, more simple composite measures with more stringent cut-offs were therefore developed for use in clinical practice (Table 1): the simplified disease activity index (SDAI) in 2003 [5], the clinical disease activity index (CDAI), which does not include an APR, in 2005 [6], as well as newer remission criteria by the American College of Rheumatology (ACR) and the European League Against Rheumatism (EULAR) in 2011 [2]. These criteria comprise either a Boolean approach or an index-based definition (cut-offs for remission: ≤ 3.3 for SDAI and ≤ 2.8 for CDAI) [11]. Compared with the original DAS 28 cut-off, patients fulfilling the newer cut-offs were found to have less residual disease activity as well as less functional disability and joint damage [12–14].

Modern RA therapy is characterized by a “treat-to-target” approach with regular assessment of disease activity using the composite measures mentioned above and, if the target is not achieved within a particular timeframe, subsequent therapeutic adaptation with the goal of reducing disease activity as early as possible [1]. Newer treatments, such as interleukin (IL)-6 and Janus kinase (JAK) inhibitors, directly inhibit APR production and may thus lead to better DAS 28 scores not reflected by clinical improvement [9, 15, 16]. The use of the DAS 28 may therefore lead to a higher proportion of patients fulfilling remission and LDA criteria than the use of other composite measures. The results of the DAS 28 may therefore be misleading when RA treatments with different modes of action are compared. To date, no systematic comparison of all four composite measures (DAS 28, CDAI, SDAI and Boolean approach) for measuring the effects of biologics and JAK inhibitors has been performed.

To inform the discussion on the choice of composite measure, we thus performed such an investigation. For this purpose, we largely used data generated for a systematic review of biologics in RA conducted by the German health technology assessment (HTA) agency, the Institute for Quality and Efficiency in Health Care (IQWiG) as well as data from systematic reviews of newer biologics and JAK inhibitors (see below for details). The studies included in these reviews investigated treatment effects on remission and LDA in patients with RA using different composite measures. We aimed to quantify the impact of the choice of composite measure. Furthermore, we discuss the consequences of potential differences in the results of the various composite measures in the studies analysed.

## Methods

### Study design and data sources

Data on remission and LDA assessed via the DAS 28, SDAI, CDAI and the Boolean approach (remission only) were included from a systematic review in an HTA report conducted by IQWiG on biologics in RA therapy. The full report is only available in German [17]—the core report [18], as well as a journal article on a network analysis based on the HTA report [19], are available in English. Clinical studies conducted up to 2017 on biologics approved up to 2016 by the European Medicines Agency (EMA) were included. These studies investigated head-to-head comparisons of biologics and comparisons of biologics with placebo. Results after 6 months of treatment were included in the analyses. Study sponsors reanalysed and provided the proportion of patients in remission or with LDA for the different composite measures used in the studies.

In addition, for newer biologics and JAK inhibitors approved by the EMA between 2017 and 2020, data on remission and LDA were considered that had been provided by study sponsors for inclusion in 5 early benefit assessments (also called dossier assessments) [20–24]. In Germany, this type of assessment is conducted within 3 months of market entry of a new drug based on a dossier submitted by the study sponsor and contains a systematic review of the evidence on a new drug versus standard care. The studies included in the assessments investigated head-to-head comparisons of biologics and comparisons of biologics with JAK inhibitors. Results after 6 to 12 months of treatment were included in the analyses.

According to the therapeutic indications specified in the summaries of product characteristics for biologics and JAK inhibitors approved in the European Union, the studies included in the systematic reviews above considered either methotrexate (MTX) naïve patients, patients after MTX failure, patients after biologic failure and / or patients with MTX intolerance. The treatments were administered in combination either with MTX or as monotherapy in patients intolerant to MTX. In placebo-controlled studies, the placebo was also administered in combination with MTX.

### Statistical analysis

Treatment effects for the outcomes of LDA and remission were estimated by odds ratios (ORs) for each of the composite measures. For studies comparing biologics with placebo, an OR > 1 indicates a beneficial effect of the biologic. For studies comparing biologics with each other and studies comparing JAK inhibitors and biologics, an OR > 1 indicates a beneficial effect for the first treatment mentioned.

Within each study we estimated the differences in estimates for all composite measures used for the assessment of remission and LDA calculating the ratio of ORs (ROR) for each comparison (e.g. ROR = OR_DAS 28 < 3.2_/OR_CDAI ≤ 10_ for LDA). An estimate of ROR > 1 thus indicates larger effect estimates for remission or LDA for the first composite measure versus the second one. For the main analyses, calculations considering data dependency were conducted (see Additional file 1 for more details on statistical methods). Sensitivity analyses not considering data dependency were also conducted (see Additional file 1: Tables 5 to 10).

RORs were calculated within each study for each possible comparison of composite measures and subsequently combined for each treatment comparison, using inverse variance weighted fixed-effect model meta-analyses for the whole patient population and, if possible, for the different subpopulations with available data (MTX naïve, after MTX failure, after biologic failure, with MTX intolerance). If separate data for different subpopulations were available from one study, both data sets were included in the analysis separately. Heterogeneity was assessed using the Q test [25] between all data sets on a treatment comparison. If data for different subpopulations were available for a treatment comparison, heterogeneity was also tested between the data set pools for different subpopulations. In the case of relevant heterogeneity (*p* < 0.05), no combined estimate was calculated. We used the statistical software R 4.1.1 [26] for all analyses on the study level and SAS 9.4 (SAS Institute, Cary NC) for meta-analyses. Data for the outcomes in the individual studies are included in Additional file 1: Tables 11 to 20.

## Results

### Placebo-controlled studies

An overview of results is provided in Table 2 and details are provided in Additional file 1: Tables 1 and 2.

**Table 2:** Overview of results on RORs for assessment of low disease activity and remission using the DAS 28, SDAI, CDAI and the Boolean approach (remission only), placebo-controlled studies

Biologic versus placebo; in combination with MTX	Low disease activity, ROR (*p* value)					Remission, ROR (*p* value)							
	Number of studies	Number of patients	DAS 28 versus CDAI	DAS 28 versus SDAI	SDAI versus CDAI	Number of studies	Number of patients	DAS 28 versus CDAI	DAS 28 versus SDAI	SDAI versus CDAI	DAS 28 versus Boolean approach	SDAI versus Boolean approach	CDAI versus Boolean approach
*IL-6 inhibitor*													
Tocilizumab	9	3307	**2.90 (< 0.001)**	**2.54 (< 0.001)**	**1.10 (< 0.001)**	9	3272	**2.86 (< 0.001)**	**2.70 (< 0.001)**	1.06 (0.240)	**2.63 (< 0.001)**	1.05 (0.648)	1.01 (0.953)
*TNFα inhibitor*													
Adalimumab	12	4520	**1.10 (0.045)**	1.07 (0.142)	–*	13	4640	**1.24 (0.019)**	1.14 (0.139)	–*	1.02 (0.814)	0.89 (0.119)	**0.82 (0.014)**
Certolizumab pegol	6	2445	1.01 (0.952)	1.03 (0.719)	0.98 (0.595)	6	2458	1.06 (0.670)	1.13 (0.377)	0.94 (0.160)	**1.35 (0.033)**	1.20 (0.069)	**1.29 (0.020)**
Etanercept	3†	877	0.96 (0.691)	0.97 (0.737)	1.00 (0.947)	3†	877	0.79 (0.284)	0.72 (0.136)	1.09 (0.241)	1.05 (0.825)	**1.42 (0.044)**	1.31 (0.137)
Golimumab	5	987	1.03 (0.777)	0.93 (0.495)	**1.08 (0.035)**	5	987	1.00 (0.994)	0.92 (0.700)	1.07 (0.436)	0.93 (0.803)	0.96 (0.833)	0.93 (0.764)
Infliximab	1‡	171	0.65 (0.181)	0.57 (0.083)	1.15 (0.244)	1‡	171	0.70 (0.696)	0.31 (0.338)	2.23 (0.172)	0.50 (0.606)	1.61 (0.678)	0.72 (0.770)
*IL-1 receptor antagonist*													
Anakinra	2‡	1035	**1.42 (0.012)**	1.27 (0.080)	**1.12 (0.017)**	2‡	1035	**3.40 (0.017)**	**2.82 (0.046)**	1.20 (0.408)	0.65 (0.595)	**0.23 (0.024)**	**0.20 (0.015)**
*T-cell activation inhibitor*													
Abatacept	8	2363	1.16 (0.051)	1.06 (0.416)	–*	8	2364	1.01 (0.953)	0.94 (0.687)	0.99 (0.859)	0.80 (0.279)	0.81 (0.205)	0.82 (0.227)
*B-cell depleting agent*													
Rituximab	2§	528	1.38 (0.441)	1.37 (0.423)	1.01 (0.941)	2§	534	0.80 (0.744)	0.54 (0.436)	1.52 (0.416)	2.15 (0.164)	3.12 (0.112)	2.53 (0.121)

The thresholds used for the assessments were: DAS 28 < 3.2, SDAI ≤ 11 and CDAI ≤ 10 for low disease activity, DAS 28 < 2.6, SDAI ≤ 3.3, CDAI ≤ 2.8 and Boolean approach (≤ 1 swollen joint, ≤ 1 tender joint, C-reactive protein ≤ 1 mg/dl and global assessment of disease activity by the patient ≤ 1 on a scale from 0 to 10) for remission. Statistically significant RORs are shown in bold font. 95% CIs are shown in Additional file 1: Tables 1 and 2

*CDAI* clinical disease activity index; *DAS 28* disease activity score 28; *MTX* methotrexate; *RORs* ratio of odds ratios; *SDAI* simplified disease activity index

* Not interpretable due to relevant heterogeneity among studies and / or study pools (*p* < 0.05)

^†^ Includes only studies on MTX-naïve patients and patients after MTX failure

^‡^ Includes only studies on patients after MTX failure

^§^ Includes only studies on patients after biologic failure

We considered results from 49 placebo-controlled studies identified in the previous systematic review [17]. The studies included 9 different biologics: 48 studies (16,233 patients) investigated LDA and 49 (16,338 patients) investigated remission. About 65% of the patients were included after MTX or biologic failure and about 35% were MTX-naïve. Nine combinations of the 4 composite measures were compared (3 for LDA, 6 for remission) resulting in a total of 81 comparisons, of which 3 (all SDAI vs. CDAI) were not interpretable due to relevant heterogeneity. 78 comparisons were thus included in the analysis (25 for LDA and 53 for remission).

**Fig. 1:**
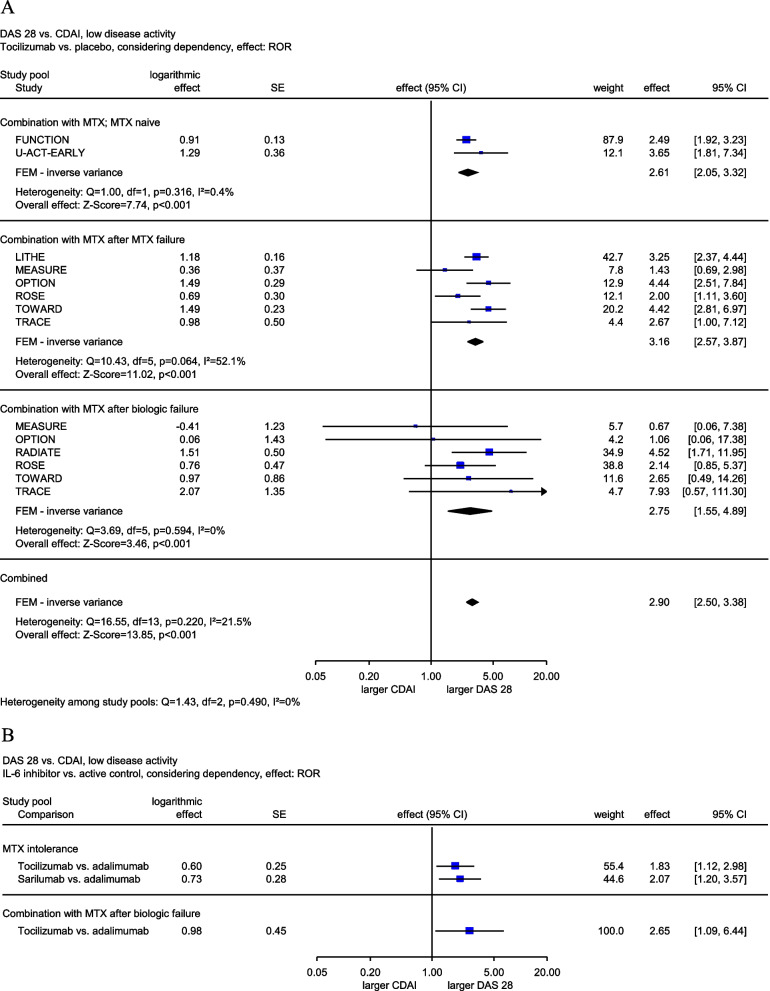
Forest plots of RORs for DAS 28 and CDAI for the assessment of low disease activity for comparisons of IL-6 inhibitors versus placebo (**A**) or active controls (**B**)

Statistically significantly larger treatment effects versus placebo were observed when using certain composite measures in 16 of the 78 comparisons (20.5%): 7 out of 25 (28.0%) for LDA and 9 out of 53 (17.0%) for remission. 11 of these 16 comparisons (68.8%) showed these effects in the DAS 28 (6 vs. CDAI, 3 vs. SDAI, 2 vs. Boolean approach): 5 for the IL-6 inhibitor tocilizumab (all with RORs > 2), 3 for the IL-1 inhibitor anakinra (2 with RORs > 2), 2 for the tumour necrosis factor (TNF)α-inhibitor adalimumab, and 1 for the TNFα-inhibitor certolizumab pegol. Four of the 16 comparisons showed statistically significantly larger treatment effects in the SDAI (3 vs. CDAI, 1 vs. Boolean approach): 2 for tocilizumab and anakinra and 2 for the TNFα inhibitors golimumab and etanercept. One of the 16 comparisons showed a statistically significantly larger treatment effect in the CDAI (vs. Boolean approach) for certolizumab pegol. To visualize the larger treatment effects measured with the DAS 28 versus the CDAI for tocilizumab in the single studies, please see the forest plot in Fig. 1a as an example.

No statistically significant differences in treatment effects were shown in 59 of the 78 comparisons and statistically significantly smaller treatment effects were shown in 3 comparisons: the SDAI or CDAI versus a Boolean approach for 1 comparison including adalimumab (RR < 1) and 2 comparisons including anakinra (RR < 0.5). However, at least for anakinra, these findings should be interpreted with caution due to the very small number of patients in remission using these composite measures (1 to 5 patients per treatment arm, see Additional file 1: Table 13), which probably contributed to the high RORs.

### Active-controlled studies

An overview of results is provided in Table 3 and details are provided in Additional file 1: Tables 3 and 4.

**Table 3:** Overview of results on ratios of odds ratios for assessment of low disease activity and remission using the DAS 28, SDAI, CDAI and the Boolean approach (remission only), active-controlled studies

Treatment comparison	Low disease activity, ROR (*p* value)					Remission, ROR (*p* value)							
	Number of studies	Number of patients	DAS 28 versus CDAI	DAS 28 versus SDAI	SDAI versus CDAI	Number of studies	Number of patients	DAS 28 versus CDAI	DAS 28 versus SDAI	SDAI versus CDAI	DAS 28 versus Boolean approach	SDAI versus Boolean approach	CDAI versus Boolean approach
*IL-6 inhibitor versus TNFα inhibitor*													
Sarilumab versus adalimumab; monotherapy	1*	169	**2.07 (0.009)**	**2.14 (0.005)**	0.97 (0.687)	1*	169	1.62 (0.478)	1.42 (0.586)	1.14 (0.641)	2.69 (0.187)	1.89 (0.314)	1.66 (0.439)
Tocilizumab versus adalimumab; monotherapy	1*	131	**1.83 (0.016)**	**1.82 (0.013)**	1.00 (0.974)	1*	131	**3.45 (0.009)**	2.27 (0.080)	**1.54 (0.020)**	2.27 (0.117)	1.00 (1.000)	0.65 (0.298)
Tocilizumab + MTX versus adalimumab + MTX	1†	69	**2.65 (0.031)**	1.28 (0.570)	**2.08 (< 0.001)**	1†	69	0.93 (0.908)	0.43 (0.318)	2.13 (0.129)	0.89 (0.872)	2.08 (0.269)	0.97 (0,962)
*TNFα inhibitor versus TNFα inhibitor*													
Certolizumab pegol + MTX versus adalimumab + MTX	1‡	836	0.96 (0.680)	1.04 (0.679)	**0.93 (0.016)**	1‡	836	1.00 (0.982)	1.06 (0.696)	0.94 (0.360)	1.19 (0.321)	1.12 (0.406)	1.19 (0.229)
*JAK inhibitor versus TNFα inhibitor*													
Baricitinib + MTX versus adalimumab + MTX	1‡	817	1.00 (0.987)	0.99 (0.917)	1.01 (0.800)	1‡	817	0.79 (0.147)	0.76 (0.073)	1.04 (0.538)	0.83 (0.311)	1.09 (0.582)	1.04 (0.778)
Filgotinib + MTX versus adalimumab + MTX	1‡	800	1.06 (0.588)	0.98 (0.878)	**1.07 (0.042)**	1‡	800	0.96 (0.796)	1.02 (0.887)	0.94 (0.310)	0.96 (0.789)	0.94 (0.627)	0.99 (0.960)
Tofacitinib + MTX versus adalimumab + MTX	2§	1152	0.85 (0.088)	0.87 (0.115)	0.98 (0.397)	2§	1152	**0.74 (0.020)**	0.82 (0.105)	0.90 (0.091)	0.77 (0.086)	0.94 (0.615)	1.05 (0.727)
Upadacitinib + MTX versus adalimumab + MTX	1‡	978	1.07 (0.476)	1.04 (0.634)	1.02 (0.462)	1‡	978	1.00 (0.999)	0.93 (0.660)	1.07 (0.293)	0.93 (0.700)	0.99 (0.971)	0.93 (0.643)
*JAK inhibitor* *versus T-cell activation inhibitor*													
Upadacitinib + MTX versus abatacept + MTX	1†	438	**1.46 (0.032)**	**1.38 (0.049)**	1.06 (0.131)	1†	438	1.37 (0.082)	1.20 (0.244)	1.14 (0.193)	1.38 (0.101)	1.16 (0.349)	1.01 (0.951)
*T-cell activation inhibitor versus TNFα inhibitor*													
Abatacept + MTX versus adalimumab + MTX	1‡	606	0.96 (0.721)	0.98 (0.860)	0.98 (0.583)	1‡	606	0.89 (0.539)	0.94 (0.753)	0.94 (0.453)	1.06 (0.811)	1.13 (0.555)	1.19 (0.386)

The thresholds used for the assessments were: DAS 28 < 3.2, SDAI ≤ 11 and CDAI ≤ 10 for low disease activity, DAS 28 < 2.6, SDAI ≤ 3.3, CDAI ≤ 2.8 and Boolean approach (≤ 1 swollen joint, ≤ 1 tender joint, C-reactive protein ≤ 1 mg/dl and global assessment of disease activity by the patient ≤ 1 on a scale from 0 to 10) for remission

Statistically significant RORs are shown in bold font

95% CIs are shown in Additional file 1: Tables 3 and 4

*CDAI* clinical disease activity index; *DAS 28* disease activity score 28; *MTX* methotrexate; *RORs* ratio of odds ratios, *SDAI* simplified disease activity index

^*^Includes only studies on patients with MTX intolerance

^†^ Includes only studies on patients after biologic failure

^‡^ Includes only studies on patients after MTX failure

^§^Includes only studies on patients after MTX or biologic failure

The 11 active-controlled studies investigated both LDA and remission and included 5 different head-to-head comparisons of biologics and 5 different comparisons (6 studies) of biologics with JAK inhibitors. A total of 5996 patients were included. About 95% of the patients were included after MTX or biologic failure and 5% were intolerant to MTX.

The same 9 combinations of composite measures were compared as in the placebo-controlled trials, resulting in 90 comparisons (30 for LDA and 60 for remission) of which all were interpretable.

Statistically significantly larger treatment effects versus the active control were observed when using certain composite measures in 11 of the 90 comparisons (12.2%); 9 out of 30 (30.0%) for LDA and 2 out of 60 (3.3%) for remission. 8 of the 11 comparisons (72.7%) showed these effects in the DAS 28 (5 vs. CDAI, 3 vs. SDAI): 6 for the IL-6 inhibitors tocilizumab and sarilumab (of which 4 showed RORs > 2) and 2 for the JAK inhibitor upadacitinib. The other 3 of the 11 comparisons showed statistically significantly larger treatment effects in the SDAI (all vs. CDAI) for tocilizumab (2 comparisons) and the JAK inhibitor filgotinib. To visualize the larger treatment effects measured with the DAS 28 versus the CDAI for tocilizumab and sarilumab in the single studies, please see the forest plot in Fig. 1b as an example.

No statistically significant differences in treatment effects were shown in 77 of the 90 comparisons and statistically significantly smaller treatment effects were shown in 2 comparisons (DAS 28 vs. CDAI for the JAK inhibitor tofacitinib and SDAI vs. CDAI for certolizumab pegol).

### Sensitivity analyses

The only statistically significant results that were robust in the sensitivity analyses were those on comparisons of composite measures including the DAS 28 for placebo-controlled trials with tocilizumab (Additional file 1: Table 5). This can be explained by the large sample size available (about 3300 patients from 9 studies) and the substantial treatment effects observed (all RORs > 2). However, all ROR estimates remained unchanged in the sensitivity analyses, only the confidence intervals were wider.

## Discussion

Our study provides the first systematic comparison of differences between the estimated treatment effects of biologics and JAK inhibitors on remission and LDA recorded with 4 composite measures. In the overall patient population and in all subpopulations, statistically significantly larger treatment effects compared to the other measures were most frequently observed if the DAS 28 was used. To a lesser extent, such larger effects were also shown in a further composite measure including an APR, the SDAI. Interestingly, statistically significant differences in treatment effects for SDAI versus CDAI were observed for the assessment of LDA, but not for remission. This may be due to smaller differences in treatment effects measured by SDAI versus CDAI than in DAS 28 versus CDAI, where differences in treatment effects might therefore be detected more easily. In addition, less precise effect estimates were observed for remission than for LDA (e.g. SDAI vs. CDAI for tocilizumab: ROR [95% CI] of 1.10 [1.05 to 1.15] for LDA and 1.06 [0.96 to 1.17] for remission). The difference in treatment effects was found to be similar for both outcomes; however, statistically significant differences in ROR were not shown for remission. This may be due to lower numbers of patients with events for this outcome in the single studies using both composite measures (see, e.g. Additional file 1: Tables 13 and 14). With regard to the treatments affected, the larger effects were most common and most pronounced for the IL-6 inhibitor tocilizumab. Smaller differences were also shown for the IL-1 inhibitor anakinra, other biologics and JAK inhibitors, although these findings were not confirmed in sensitivity analyses. Hence, the DAS 28 and SDAI in particular make it difficult to interpret comparative effectiveness studies of treatments with different modes of action (e.g. IL inhibitors vs. TNFα inhibitors), as the apparently larger treatment effects in favour of IL-1 or IL-6 inhibitors may not accurately reflect clinical improvement.

### Consequences of using inappropriate composite measures

Even though the deficits of the DAS 28 (and to a lesser extent the SDAI) have been known for several years, composite measures including an APR are still being used in primary studies [10]. This might be partly due to the fact that they are still recommended in official guidance: the current EMA guideline for the design of clinical studies on RA still mentions the DAS 28 as a validated composite measure to assess LDA and remission [27]. In addition, the ACR recommendations on RA disease activity measures, which were updated in 2019, still recommend the DAS 28, among others [28]. These guidelines should be updated to ensure study results that reflect clinical benefits for patients, rather than differences in the mode of action of treatments. The replacement of the DAS 28 and SDAI with the CDAI is all the more important because various newer treatments (e.g. JAK inhibitors such as upadacitinib and filgotinib or IL-6 inhibitors such as sarilumab) directly inhibit APR production.

Furthermore, composite measures including an APR still influence the conclusions of systematic reviews and HTA reports [29–36] and resulting documents such as clinical guidelines. In consequence, decisions based on these documents, such as reimbursement or treatment decisions, may be biased. Systematic reviewers and HTA bodies should thus avoid using the DAS 28 and SDAI, specifically for comparative effectiveness research. If only DAS 28 or SDAI results are available, HTA bodies should require study sponsors to provide CDAI results. With the present publication, we provide these results for a large number of RA studies (see Additional file 1: Tables 11 to 20), demonstrating that these important data can be generated by re-analysis of available study data, even if the original study analysis did not include CDAI results [19].

### Previous research

Our findings confirm and supplement previous research on composite measures in RA. As early as 2005, Aletaha et al. indicated in their validation of the CDAI that composite measures including APRs were dispensable [6]. Moreover, Schoels et al. (2017) found that even if lower cut-offs were used for the DAS 28, a considerable proportion of patients were classified as being in remission, despite the presence of a significant swollen joint count [37]. This is in line with Futó et al., whose visualization of the DAS 28, SDAI and CDAI showed that APRs overshadowed changes in clinical outcomes; the authors described APRs as “major confounding factors” [38]. In an exposure–response modelling of tocilizumab in RA using the DAS 28, SDAI and CDAI, Bastida et al. [39] found that APRs decrease faster than clinical outcomes and concluded that the “CDAI is a better option than the DAS 28 and SDAI to assess disease activity in tocilizumab-treated patients”.

### Strengths and limitations of our analysis

The major strength of our analysis is the systematic approach and the broad evidence base retrieved from several systematic reviews allowing consideration of all 4 composite measures, a broad range of biologics and JAK inhibitors, and a broad range of patients, i.e. MTX-naïve patients as well as patients after MTX or biologic failure. A limitation is the relatively small number of studies available for the direct comparisons.

## Conclusions

The use of composite measures including an APR to measure the treatment effects in patients with RA leads to overestimation of the treatment effects of drugs with direct inhibitive effects on APRs and thus to inaccurate classification of the main treatment outcomes compared to other RA treatments. Our findings underline the need for the use of the CDAI as the composite measure of choice in clinical studies, in particular to enable unbiased results in comparative effectiveness research.

## Supplementary Information

**Additional file 1. **Details on statistical methods, supplementary tables and figures

## Data Availability

The datasets supporting the conclusions of this article are included within the article and in Additional file 1.

## Abbreviations

ACR: American college of rheumatology
APR: Acute phase reactant
CDAI: Clinical disease activity index
DAS 28: Disease activity score 28
DMARD: Disease-modifying antirheumatic drug
EULAR: European league against rheumatism
HTA: Health technology assessment
IL: Interleukin
IQWiG: Institut für Qualität und Wirtschaftlichkeit im Gesundheitswesen (Institute for Quality and Efficiency in Health Care)
JAK: Janus kinase
LDA: Low disease activity
MTX: Methotrexate
OR: Odds ratio
RA: Rheumatoid arthritis
ROR: Ratio of odds ratios
SDAI: Simplified disease activity index
TNF: Tumour necrosis factor
