# Supplementary material for: A systematic comparison of different composite measures (DAS 28, CDAI, SDAI, and Boolean approach) for determining treatment effects on low disease activity and remission in rheumatoid arthritis
Source: BMC Rheumatol. 2022 Dec 9;6:82. doi: 10.1186/s41927-022-00314-7 (PMC9732992; doi:10.1186/s41927-022-00314-7)
Supplement: Supplementary file 1 — Additional file 1. Details on statistical methods, supplementary tables and figures [file 41927_2022_314_MOESM1_ESM.docx]

**Details on statistical methods, supplementary tables and figures**

**Table of Contents**

[List of tables 2](#_Toc97015152)

[List of figures 4](#_Toc97015153)

[Details on statistical methods 12](#_Toc97015154)

[Main analyses considering data dependency 13](#_Toc97015155)

[Tabular results 13](#_Toc97015156)

[Biologics vs. placebo 13](#_Toc97015157)

[Biologics vs. biologics / JAK inhibitors vs. biologics 17](#_Toc97015158)

[Forest plots 20](#_Toc97015159)

[Biologics vs. placebo 20](#_Toc97015160)

[JAK inhibitor (tofacitinib) vs. TNFα inhibitor (adalimumab) 70](#_Toc97015161)

[Sensitivity analyses not considering data dependency 74](#_Toc97015162)

[Summary of results 74](#_Toc97015163)

[Tabular results 76](#_Toc97015164)

[Biologics vs. placebo 76](#_Toc97015165)

[Biologics vs. biologics / JAK inhibitors vs. biologics 80](#_Toc97015166)

[Forest plots 83](#_Toc97015167)

[Biologics vs. placebo 83](#_Toc97015168)

[JAK inhibitor (tofacitinib) vs. TNFα inhibitor (adalimumab) 133](#_Toc97015169)

[Data for low disease activity and remission measured by DAS 28, SDAI, CDAI and Boolean definition (remission only) from single studies 137](#_Toc97015170)

# List of tables

[Supplement Table 1: Results on RORs for assessment of low disease activity using the DAS 28 < 3.2, SDAI ≤ 11 or CDAI ≤ 10 in MTX-naïve patients, patients after MTX failure and patients after biologic failure, main analysis 14](#_Toc96930301)

[Supplement Table 2: Results on RORs for assessment of remission using the DAS 28 < 2.6, SDAI ≤ 3.3, CDAI ≤ 2.8 or Boolean approach in MTX-naïve patients, patients after MTX failure and patients after biologic failure, main analysis 15](#_Toc96930302)

[Supplement Table 3: Results on RORs for assessment of low disease activity using the DAS 28 < 3.2, SDAI ≤ 11 or CDAI ≤ 10 for direct comparisons of biological medicines among each other or comparisons with JAK inhibitors in patients after MTX failure, biologic failure or patients intolerant to MTX, main analysis 17](#_Toc96930303)

[Supplement Table 4: Results on RORs for assessment of remission using the DAS 28 < 2.6, SDAI ≤ 3.3, CDAI ≤ 2.8 or Boolean approach for direct comparisons of biological medicines among each other or comparisons with JAK inhibitors in patients after MTX failure, biologic failure or patients intolerant to MTX, main analysis 18](#_Toc96930304)

[Supplement Table 5: Overview of results on RORs for assessment of low disease activity and remission using the DAS 28, SDAI, CDAI and the Boolean approach (remission only), placebo-controlled studies 74](#_Toc96930305)

[Supplement Table 6: Overview of results on RORs for assessment of low disease activity and remission using the DAS 28, SDAI, CDAI and the Boolean approach (remission only), active-controlled studies 75](#_Toc96930306)

[Supplement Table 7: Results on RORs for assessment of low disease activity using the DAS 28 < 3.2, SDAI ≤ 11 or CDAI ≤ 10 in MTX-naïve patients, patients after MTX failure and patients after biologic failure, sensitivity analysis 77](#_Toc96930307)

[Supplement Table 8: Results on RORs for assessment of remission using the DAS 28 < 2.6, SDAI ≤ 3.3, CDAI ≤ 2.8 or Boolean definition in MTX-naïve patients, patients after MTX failure and patients after biologic failure, sensitivity analysis 78](#_Toc96930308)

[Supplement Table 9: Results on RORs for assessment of low disease activity using the DAS 28 < 3.2, SDAI ≤ 11 or CDAI ≤ 10 for direct comparisons of biological medicines among each other or comparisons with JAK inhibitors in patients after MTX failure, biologic failure or patients intolerant to MTX, sensitivity analysis 80](#_Toc96930309)

[Supplement Table 10: Results on RORs for assessment of remission using the DAS 28 < 2.6, SDAI ≤ 3.3, CDAI ≤ 2.8 or Boolean definition for direct comparisons of biological medicines among each other or comparisons with JAK inhibitors in patients after MTX failure, biologic failure or patients intolerant to MTX, sensitivity analysis 81](#_Toc96930310)

[Supplement Table 11: Results on remission available for the DAS 28 < 2.6. SDAI ≤ 3.3. CDAI ≤ 2.8 or Boolean definition from single studies investigating MTX-naïve patients 137](#_Toc96930311)

[Supplement Table 12: Results on low disease activity available for the DAS 28 < 3.2. SDAI ≤ 11 or CDAI ≤ 10 from single studies investigating MTX-naïve patients 142](#_Toc96930312)

[Supplement Table 13: Results on remission available for the DAS 28 < 2.6. SDAI ≤ 3.3. CDAI ≤ 2.8 or Boolean definition from single studies investigating patients after MTX failure 145](#_Toc96930313)

[Supplement Table 14: Results on low disease activity available for the DAS 28 < 3.2. SDAI ≤ 11 or CDAI ≤ 10 from single studies investigating patients after MTX failure 154](#_Toc96930314)

[Supplement Table 15: Results on remission available for the DAS 28 < 2.6. SDAI ≤ 3.3. CDAI ≤ 2.8 or Boolean definition from single studies investigating patients with MTX intolerance 160](#_Toc96930315)

[Supplement Table 16: Results on low disease activity available for the DAS 28 < 3.2. SDAI ≤ 11 or CDAI ≤ 10 from single studies investigating patients with MTX intolerance 161](#_Toc96930316)

[Supplement Table 17: Results on remission available for the DAS 28 < 2.6. SDAI ≤ 3.3. CDAI ≤ 2.8 or Boolean definition from single studies investigating patients after biologic failure 162](#_Toc96930317)

[Supplement Table 18: Results on low disease activity available for the DAS 28 < 3.2. SDAI ≤ 11 or CDAI ≤ 10 from single studies investigating patients after biologic failure 165](#_Toc96930318)

[Supplement Table 19: Results on remission available for the DAS 28 < 2.6. SDAI ≤ 3.3. CDAI ≤ 2.8 or Boolean definition from single studies investigating patients after MTX or biologic failure 167](#_Toc96930319)

[Supplement Table 20: Results on low disease activity available for the DAS 28 < 3.2. SDAI ≤ 11 or CDAI ≤ 10 from single studies investigating patients after MTX or biologic failure 168](#_Toc96930320)

# List of figures

[Supplement Figure 1: DAS 28 vs. CDAI, low disease activity, abatacept vs. placebo (combined with MTX), main analysis 20](#_Toc97015171)

[Supplement Figure 2: DAS 28 vs. SDAI, low disease activity, abatacept vs. placebo (combined with MTX), main analysis 21](#_Toc97015172)

[Supplement Figure 3: SDAI vs. CDAI, low disease activity, abatacept vs. placebo (combined with MTX), main analysis 22](#_Toc97015173)

[Supplement Figure 4: DAS 28 vs. CDAI, remission, abatacept vs. placebo (combined with MTX), main analysis 23](#_Toc97015174)

[Supplement Figure 5: DAS 28 vs. SDAI, remission, abatacept vs. placebo (combined with MTX), main analysis 24](#_Toc97015175)

[Supplement Figure 6: SDAI vs. CDAI, remission, abatacept vs. placebo (combined with MTX), main analysis 25](#_Toc97015176)

[Supplement Figure 7: DAS 28 vs. Boolean approach, remission, abatacept vs. placebo (combined with MTX), main analysis 26](#_Toc97015177)

[Supplement Figure 8: SDAI vs. Boolean approach, remission, abatacept vs. placebo (combined with MTX), main analysis 27](#_Toc97015178)

[Supplement Figure 9: CDAI vs. Boolean approach, remission, abatacept vs. placebo (combined with MTX), main analysis 28](#_Toc97015179)

[Supplement Figure 10: DAS 28 vs. CDAI, low disease activity, adalimumab vs. placebo (combined with MTX), main analysis 29](#_Toc97015180)

[Supplement Figure 11: DAS 28 vs. SDAI, low disease activity, adalimumab vs. placebo (combined with MTX), main analysis 30](#_Toc97015181)

[Supplement Figure 12: SDAI vs. CDAI, low disease activity, adalimumab vs. placebo (combined with MTX), main analysis 31](#_Toc97015182)

[Supplement Figure 13: DAS 28 vs. CDAI, remission, adalimumab vs. placebo (combined with MTX), main analysis 32](#_Toc97015183)

[Supplement Figure 14: DAS 28 vs. SDAI, remission, adalimumab vs. placebo (combined with MTX), main analysis 33](#_Toc97015184)

[Supplement Figure 15: SDAI vs. CDAI, remission, adalimumab vs. placebo (combined with MTX), main analysis 34](#_Toc97015185)

[Supplement Figure 16: DAS 28 vs. Boolean approach, remission, adalimumab vs. placebo (combined with MTX), main analysis 35](#_Toc97015186)

[Supplement Figure 17: SDAI vs. Boolean approach, remission, adalimumab vs. placebo (combined with MTX), main analysis 36](#_Toc97015187)

[Supplement Figure 18: CDAI vs. Boolean approach, remission, adalimumab vs. placebo (combined with MTX), main analysis 37](#_Toc97015188)

[Supplement Figure 19: DAS 28 vs. CDAI, low disease activity, anakinra vs. placebo (combined with MTX), main analysis 37](#_Toc97015189)

[Supplement Figure 20: DAS 28 vs. SDAI, low disease activity, anakinra vs. placebo (combined with MTX), main analysis 38](#_Toc97015190)

[Supplement Figure 21: SDAI vs. CDAI, low disease activity, anakinra vs. placebo (combined with MTX), main analysis 38](#_Toc97015191)

[Supplement Figure 22: DAS 28 vs. CDAI, remission, anakinra vs. placebo (combined with MTX), main analysis 38](#_Toc97015192)

[Supplement Figure 23: DAS 28 vs. SDAI, remission, anakinra vs. placebo (combined with MTX), main analysis 39](#_Toc97015193)

[Supplement Figure 24: SDAI vs. CDAI, remission, anakinra vs. placebo (combined with MTX), main analysis 39](#_Toc97015194)

[Supplement Figure 25: DAS 28 vs. Boolean approach, remission, anakinra vs. placebo (combined with MTX), main analysis 39](#_Toc97015195)

[Supplement Figure 26: SDAI vs. Boolean approach, remission, anakinra vs. placebo (combined with MTX), main analysis 40](#_Toc97015196)

[Supplement Figure 27: CDAI vs. Boolean approach, remission, anakinra vs. placebo (combined with MTX), main analysis 40](#_Toc97015197)

[Supplement Figure 28: DAS 28 vs. CDAI, low disease activity, certolizumab pegol vs. placebo (combined with MTX), main analysis 41](#_Toc97015198)

[Supplement Figure 29: DAS 28 vs. SDAI, low disease activity, certolizumab pegol vs. placebo (combined with MTX), main analysis 42](#_Toc97015199)

[Supplement Figure 30: SDAI vs. CDAI, low disease activity, certolizumab pegol vs. placebo (combined with MTX), main analysis 43](#_Toc97015200)

[Supplement Figure 31: DAS 28 vs. CDAI, remission, certolizumab pegol vs. placebo (combined with MTX), main analysis 44](#_Toc97015201)

[Supplement Figure 32: DAS 28 vs. SDAI, remission, certolizumab pegol vs. placebo (combined with MTX), main analysis 45](#_Toc97015202)

[Supplement Figure 33: SDAI vs. CDAI, remission, certolizumab pegol vs. placebo (combined with MTX), main analysis 46](#_Toc97015203)

[Supplement Figure 34: DAS 28 vs. Boolean approach, remission, certolizumab pegol vs. placebo (combined with MTX), main analysis 47](#_Toc97015204)

[Supplement Figure 35: SDAI vs. Boolean approach, remission, certolizumab pegol vs. placebo (combined with MTX), main analysis 48](#_Toc97015205)

[Supplement Figure 36: CDAI vs. Boolean approach, remission, certolizumab pegol vs. placebo (combined with MTX), main analysis 49](#_Toc97015206)

[Supplement Figure 37: DAS 28 vs. CDAI, low disease activity, etanercept vs. placebo (combined with MTX), main analysis 49](#_Toc97015207)

[Supplement Figure 38: DAS 28 vs. SDAI, low disease activity, etanercept vs. placebo (combined with MTX), main analysis 50](#_Toc97015208)

[Supplement Figure 39: SDAI vs. CDAI, low disease activity, etanercept vs. placebo (combined with MTX), main analysis 50](#_Toc97015209)

[Supplement Figure 40: DAS 28 vs. CDAI, remission, etanercept vs. placebo (combined with MTX), main analysis 51](#_Toc97015210)

[Supplement Figure 41: DAS 28 vs. SDAI, remission, etanercept vs. placebo (combined with MTX), main analysis 51](#_Toc97015211)

[Supplement Figure 42: SDAI vs. CDAI, remission, etanercept vs. placebo (combined with MTX), main analysis 52](#_Toc97015212)

[Supplement Figure 43: DAS 28 vs. Boolean approach, remission, etanercept vs. placebo (combined with MTX), main analysis 52](#_Toc97015213)

[Supplement Figure 44: SDAI vs. Boolean approach, remission, etanercept vs. placebo (combined with MTX), main analysis 53](#_Toc97015214)

[Supplement Figure 45: CDAI vs. Boolean approach, remission, etanercept vs. placebo (combined with MTX), main analysis 53](#_Toc97015215)

[Supplement Figure 46: DAS 28 vs. CDAI, low disease activity, golimumab vs. placebo (combined with MTX), main analysis 54](#_Toc97015216)

[Supplement Figure 47: DAS 28 vs. SDAI, low disease activity, golimumab vs. placebo (combined with MTX), main analysis 54](#_Toc97015217)

[Supplement Figure 48: SDAI vs. CDAI, low disease activity, golimumab vs. placebo (combined with MTX), main analysis 55](#_Toc97015218)

[Supplement Figure 49: DAS 28 vs. CDAI, remission, golimumab vs. placebo (combined with MTX), main analysis 56](#_Toc97015219)

[Supplement Figure 50: DAS 28 vs. SDAI, remission, golimumab vs. placebo (combined with MTX), main analysis 56](#_Toc97015220)

[Supplement Figure 51: SDAI vs. CDAI, remission, golimumab vs. placebo (combined with MTX), main analysis 57](#_Toc97015221)

[Supplement Figure 52: DAS 28 vs. Boolean approach, remission, golimumab vs. placebo (combined with MTX), main analysis 57](#_Toc97015222)

[Supplement Figure 53: SDAI vs. Boolean approach, remission, golimumab vs. placebo (combined with MTX), main analysis 58](#_Toc97015223)

[Supplement Figure 54: CDAI vs. Boolean approach remission, golimumab vs. placebo (combined with MTX), main analysis 58](#_Toc97015224)

[Supplement Figure 55: DAS 28 vs. CDAI, low disease activity, rituximab vs. placebo (combined with MTX), main analysis 59](#_Toc97015225)

[Supplement Figure 56: DAS 28 vs. SDAI, low disease activity, rituximab vs. placebo (combined with MTX), main analysis 59](#_Toc97015226)

[Supplement Figure 57: SDAI vs. CDAI, low disease activity, rituximab vs. placebo (combined with MTX), main analysis 59](#_Toc97015227)

[Supplement Figure 58: DAS 28 vs. CDAI, remission, rituximab vs. placebo (combined with MTX), main analysis 60](#_Toc97015228)

[Supplement Figure 59: DAS 28 vs. SDAI, remission, rituximab vs. placebo (combined with MTX), main analysis 60](#_Toc97015229)

[Supplement Figure 60: SDAI vs. CDAI, remission, rituximab vs. placebo (combined with MTX), main analysis 60](#_Toc97015230)

[Supplement Figure 61: DAS 28 vs. Boolean approach, remission, rituximab vs. placebo (combined with MTX), main analysis 61](#_Toc97015231)

[Supplement Figure 62: SDAI vs. Boolean approach, remission, rituximab vs. placebo (combined with MTX), main analysis 61](#_Toc97015232)

[Supplement Figure 63: CDAI vs. Boolean approach, remission, rituximab vs. placebo (combined with MTX), main analysis 61](#_Toc97015233)

[Supplement Figure 64: DAS 28 vs. CDAI, low disease activity, tocilizumab vs. placebo (combined with MTX), main analysis 62](#_Toc97015234)

[Supplement Figure 65: DAS 28 vs. SDAI, low disease activity, tocilizumab vs. placebo (combined with MTX), main analysis 63](#_Toc97015235)

[Supplement Figure 66: SDAI vs. CDAI, low disease activity, tocilizumab vs. placebo (combined with MTX), main analysis 64](#_Toc97015236)

[Supplement Figure 67: DAS 28 vs. CDAI, remission, tocilizumab vs. placebo (combined with MTX), main analysis 65](#_Toc97015237)

[Supplement Figure 68: DAS 28 vs. SDAI, remission, tocilizumab vs. placebo (combined with MTX), main analysis 66](#_Toc97015238)

[Supplement Figure 69: SDAI vs. CDAI, remission, tocilizumab vs. placebo (combined with MTX), main analysis 67](#_Toc97015239)

[Supplement Figure 70: DAS 28 vs. Boolean approach, remission, tocilizumab vs. placebo (combined with MTX), main analysis 68](#_Toc97015240)

[Supplement Figure 71: SDAI vs. Boolean approach, remission, tocilizumab vs. placebo (combined with MTX), main analysis 69](#_Toc97015241)

[Supplement Figure 72: CDAI vs. Boolean approach, remission, tocilizumab vs. placebo (combined with MTX), main analysis 70](#_Toc97015242)

[Supplement Figure 73: DAS 28 vs. CDAI, low disease activity, tofacitinib vs. adalimumab (combined with MTX), main analysis 70](#_Toc97015243)

[Supplement Figure 74: DAS 28 vs. SDAI, low disease activity, tofacitinib vs. adalimumab (combined with MTX), main analysis 71](#_Toc97015244)

[Supplement Figure 75: SDAI vs. CDAI, low disease activity, tofacitinib vs. adalimumab (combined with MTX), main analysis 71](#_Toc97015245)

[Supplement Figure 76: DAS 28 vs. CDAI, remission, tofacitinib vs. adalimumab (combined with MTX), main analysis 71](#_Toc97015246)

[Supplement Figure 77: DAS 28 vs. SDAI, remission, tofacitinib vs. adalimumab (combined with MTX), main analysis 72](#_Toc97015247)

[Supplement Figure 78: SDAI vs. CDAI, remission, tofacitinib vs. adalimumab (combined with MTX), main analysis 72](#_Toc97015248)

[Supplement Figure 79: DAS 28 vs. Boolean approach, remission, tofacitinib vs. adalimumab (combined with MTX), main analysis 72](#_Toc97015249)

[Supplement Figure 80: SDAI vs. Boolean approach, remission, tofacitinib vs. adalimumab (combined with MTX), main analysis 73](#_Toc97015250)

[Supplement Figure 81: CDAI vs. Boolean approach, remission, tofacitinib vs. adalimumab (combined with MTX), main analysis 73](#_Toc97015251)

[Supplement Figure 82: DAS 28 vs. CDAI, low disease activity, abatacept vs. placebo (combined with MTX), sensitivity analysis 83](#_Toc97015252)

[Supplement Figure 83: DAS 28 vs. SDAI, low disease activity, abatacept vs. placebo (combined with MTX), sensitivity analysis 84](#_Toc97015253)

[Supplement Figure 84: SDAI vs. CDAI, low disease activity, abatacept vs. placebo (combined with MTX), sensitivity analysis 85](#_Toc97015254)

[Supplement Figure 85: DAS 28 vs. CDAI, remission, abatacept vs. placebo (combined with MTX), sensitivity analysis 86](#_Toc97015255)

[Supplement Figure 86: DAS 28 vs. SDAI, remission, abatacept vs. placebo (combined with MTX), sensitivity analysis 87](#_Toc97015256)

[Supplement Figure 87: SDAI vs. CDAI, remission, abatacept vs. placebo (combined with MTX), sensitivity analysis 88](#_Toc97015257)

[Supplement Figure 88: DAS 28 vs. Boolean approach, remission, abatacept vs. placebo (combined with MTX), sensitivity analysis 89](#_Toc97015258)

[Supplement Figure 89: SDAI vs. Boolean approach, remission, abatacept vs. placebo (combined with MTX), sensitivity analysis 90](#_Toc97015259)

[Supplement Figure 90: CDAI vs. Boolean approach, remission, abatacept vs. placebo (combined with MTX), sensitivity analysis 91](#_Toc97015260)

[Supplement Figure 91: DAS 28 vs. CDAI, low disease activity, adalimumab vs. placebo (combined with MTX), sensitivity analysis 92](#_Toc97015261)

[Supplement Figure 92: DAS 28 vs. SDAI, low disease activity, adalimumab vs. placebo (combined with MTX), sensitivity analysis 93](#_Toc97015262)

[Supplement Figure 93: SDAI vs. CDAI, low disease activity, adalimumab vs. placebo (combined with MTX), sensitivity analysis 94](#_Toc97015263)

[Supplement Figure 94: DAS 28 vs. CDAI, remission, adalimumab vs. placebo (combined with MTX), sensitivity analysis 95](#_Toc97015264)

[Supplement Figure 95: DAS 28 vs. SDAI, remission, adalimumab vs. placebo (combined with MTX), sensitivity analysis 96](#_Toc97015265)

[Supplement Figure 96: SDAI vs. CDAI, remission, adalimumab vs. placebo (combined with MTX), sensitivity analysis 97](#_Toc97015266)

[Supplement Figure 97: DAS 28 vs. Boolean approach, remission, adalimumab vs. placebo (combined with MTX), sensitivity analysis 98](#_Toc97015267)

[Supplement Figure 98: SDAI vs. Boolean approach, remission, adalimumab vs. placebo (combined with MTX), sensitivity analysis 99](#_Toc97015268)

[Supplement Figure 99: CDAI vs. Boolean approach, remission, adalimumab vs. placebo (combined with MTX), sensitivity analysis 100](#_Toc97015269)

[Supplement Figure 100: DAS 28 vs. CDAI, low disease activity, anakinra vs. placebo (combined with MTX), sensitivity analysis 100](#_Toc97015270)

[Supplement Figure 101: DAS 28 vs. SDAI, low disease activity, anakinra vs. placebo (combined with MTX), sensitivity analysis 101](#_Toc97015271)

[Supplement Figure 102: SDAI vs. CDAI, low disease activity, anakinra vs. placebo (combined with MTX), sensitivity analysis 101](#_Toc97015272)

[Supplement Figure 103: DAS 28 vs. CDAI, remission, anakinra vs. placebo (combined with MTX), sensitivity analysis 101](#_Toc97015273)

[Supplement Figure 104: DAS 28 vs. SDAI, remission, anakinra vs. placebo (combined with MTX), sensitivity analysis 102](#_Toc97015274)

[Supplement Figure 105: SDAI vs. CDAI, remission, anakinra vs. placebo (combined with MTX), sensitivity analysis 102](#_Toc97015275)

[Supplement Figure 106: DAS 28 vs. Boolean approach, remission, anakinra vs. placebo (combined with MTX), sensitivity analysis 102](#_Toc97015276)

[Supplement Figure 107: SDAI vs. Boolean approach, remission, anakinra vs. placebo (combined with MTX), sensitivity analysis 103](#_Toc97015277)

[Supplement Figure 108: CDAI vs. Boolean approach, remission, anakinra vs. placebo (combined with MTX), sensitivity analysis 103](#_Toc97015278)

[Supplement Figure 109: DAS 28 vs. CDAI, low disease activity, certolizumab pegol vs. placebo (combined with MTX), sensitivity analysis 104](#_Toc97015279)

[Supplement Figure 110: DAS 28 vs. SDAI, low disease activity, certolizumab pegol vs. placebo (combined with MTX), sensitivity analysis 105](#_Toc97015280)

[Supplement Figure 111: SDAI vs. CDAI, low disease activity, certolizumab pegol vs. placebo (combined with MTX), sensitivity analysis 106](#_Toc97015281)

[Supplement Figure 112: DAS 28 vs. CDAI, remission, certolizumab pegol vs. placebo (combined with MTX), sensitivity analysis 107](#_Toc97015282)

[Supplement Figure 113: DAS 28 vs. SDAI, remission, certolizumab pegol vs. placebo (combined with MTX), sensitivity analysis 108](#_Toc97015283)

[Supplement Figure 114: SDAI vs. CDAI, remission, certolizumab pegol vs. placebo (combined with MTX), sensitivity analysis 109](#_Toc97015284)

[Supplement Figure 115: DAS 28 vs. Boolean approach, remission, certolizumab pegol vs. placebo (combined with MTX), sensitivity analysis 110](#_Toc97015285)

[Supplement Figure 116: SDAI vs. Boolean approach, remission, certolizumab pegol vs. placebo (combined with MTX), sensitivity analysis 111](#_Toc97015286)

[Supplement Figure 117: CDAI vs. Boolean approach, remission, certolizumab pegol vs. placebo (combined with MTX), sensitivity analysis 112](#_Toc97015287)

[Supplement Figure 118: DAS 28 vs. CDAI, low disease activity, etanercept vs. placebo (combined with MTX), sensitivity analysis 113](#_Toc97015288)

[Supplement Figure 119: DAS 28 vs. SDAI, low disease activity, etanercept vs. placebo (combined with MTX), sensitivity analysis 113](#_Toc97015289)

[Supplement Figure 120: SDAI vs. CDAI, low disease activity, etanercept vs. placebo (combined with MTX), sensitivity analysis 114](#_Toc97015290)

[Supplement Figure 121: DAS 28 vs. CDAI, remission, etanercept vs. placebo (combined with MTX), sensitivity analysis 114](#_Toc97015291)

[Supplement Figure 122: DAS 28 vs. SDAI, remission, etanercept vs. placebo (combined with MTX), sensitivity analysis 115](#_Toc97015292)

[Supplement Figure 123: SDAI vs. CDAI, remission, etanercept vs. placebo (combined with MTX), sensitivity analysis 115](#_Toc97015293)

[Supplement Figure 124: DAS 28 vs. Boolean approach, remission, etanercept vs. placebo (combined with MTX), sensitivity analysis 116](#_Toc97015294)

[Supplement Figure 125: SDAI vs. Boolean approach, remission, etanercept vs. placebo (combined with MTX), sensitivity analysis 116](#_Toc97015295)

[Supplement Figure 126: CDAI vs. Boolean approach, remission, etanercept vs. placebo (combined with MTX), sensitivity analysis 117](#_Toc97015296)

[Supplement Figure 127: DAS 28 vs. CDAI, low disease activity, golimumab vs. placebo (combined with MTX), sensitivity analysis 117](#_Toc97015297)

[Supplement Figure 128: DAS 28 vs. SDAI, low disease activity, golimumab vs. placebo (combined with MTX), sensitivity analysis 118](#_Toc97015298)

[Supplement Figure 129: SDAI vs. CDAI, low disease activity, golimumab vs. placebo (combined with MTX), sensitivity analysis 118](#_Toc97015299)

[Supplement Figure 130: DAS 28 vs. CDAI, remission, golimumab vs. placebo (combined with MTX), sensitivity analysis 119](#_Toc97015300)

[Supplement Figure 131: DAS 28 vs. SDAI, remission, golimumab vs. placebo (combined with MTX), sensitivity analysis 119](#_Toc97015301)

[Supplement Figure 132: SDAI vs. CDAI, remission, golimumab vs. placebo (combined with MTX), sensitivity analysis 120](#_Toc97015302)

[Supplement Figure 133: DAS 28 vs. Boolean approach, remission, golimumab vs. placebo (combined with MTX), sensitivity analysis 120](#_Toc97015303)

[Supplement Figure 134: SDAI vs. Boolean approach, remission, golimumab vs. placebo (combined with MTX), sensitivity analysis 121](#_Toc97015304)

[Supplement Figure 135: CDAI vs. Boolean approach remission, golimumab vs. placebo (combined with MTX), sensitivity analysis 121](#_Toc97015305)

[Supplement Figure 136: DAS 28 vs. CDAI, low disease activity, rituximab vs. placebo (combined with MTX), sensitivity analysis 122](#_Toc97015306)

[Supplement Figure 137: DAS 28 vs. SDAI, low disease activity, rituximab vs. placebo (combined with MTX), sensitivity analysis 122](#_Toc97015307)

[Supplement Figure 138: SDAI vs. CDAI, low disease activity, rituximab vs. placebo (combined with MTX), sensitivity analysis 122](#_Toc97015308)

[Supplement Figure 139: DAS 28 vs. CDAI, remission, rituximab vs. placebo (combined with MTX), sensitivity analysis 123](#_Toc97015309)

[Supplement Figure 140: DAS 28 vs. SDAI, remission, rituximab vs. placebo (combined with MTX), sensitivity analysis 123](#_Toc97015310)

[Supplement Figure 141: SDAI vs. CDAI, remission, rituximab vs. placebo (combined with MTX), sensitivity analysis 123](#_Toc97015311)

[Supplement Figure 142: DAS 28 vs. Boolean approach, remission, rituximab vs. placebo (combined with MTX), sensitivity analysis 124](#_Toc97015312)

[Supplement Figure 143: SDAI vs. Boolean approach, remission, rituximab vs. placebo (combined with MTX), sensitivity analysis 124](#_Toc97015313)

[Supplement Figure 144: CDAI vs. Boolean approach, remission, rituximab vs. placebo (combined with MTX), sensitivity analysis 124](#_Toc97015314)

[Supplement Figure 145: DAS 28 vs. CDAI, low disease activity, tocilizumab vs. placebo (combined with MTX), sensitivity analysis 125](#_Toc97015315)

[Supplement Figure 146: DAS 28 vs. SDAI, low disease activity, tocilizumab vs. placebo (combined with MTX), sensitivity analysis 126](#_Toc97015316)

[Supplement Figure 147: SDAI vs. CDAI, low disease activity, tocilizumab vs. placebo (combined with MTX), sensitivity analysis 127](#_Toc97015317)

[Supplement Figure 148: DAS 28 vs. CDAI, remission, tocilizumab vs. placebo (combined with MTX), sensitivity analysis 128](#_Toc97015318)

[Supplement Figure 149: DAS 28 vs. SDAI, remission, tocilizumab vs. placebo (combined with MTX), sensitivity analysis 129](#_Toc97015319)

[Supplement Figure 150: SDAI vs. CDAI, remission, tocilizumab vs. placebo (combined with MTX), sensitivity analysis 130](#_Toc97015320)

[Supplement Figure 151: DAS 28 vs. Boolean approach, remission, tocilizumab vs. placebo (combined with MTX), sensitivity analysis 131](#_Toc97015321)

[Supplement Figure 152: SDAI vs. Boolean approach, remission, tocilizumab vs. placebo (combined with MTX), sensitivity analysis 132](#_Toc97015322)

[Supplement Figure 153: CDAI vs. Boolean approach, remission, tocilizumab vs. placebo (combined with MTX), sensitivity analysis 133](#_Toc97015323)

[Supplement Figure 154: DAS 28 vs. CDAI, low disease activity, tofacitinib vs. adalimumab (combined with MTX), sensitivity analysis 133](#_Toc97015324)

[Supplement Figure 155: DAS 28 vs. SDAI, low disease activity, tofacitinib vs. adalimumab (combined with MTX), sensitivity analysis 134](#_Toc97015325)

[Supplement Figure 156: SDAI vs. CDAI, low disease activity, tofacitinib vs. adalimumab (combined with MTX), sensitivity analysis 134](#_Toc97015326)

[Supplement Figure 157: DAS 28 vs. CDAI, remission, tofacitinib vs. adalimumab (combined with MTX), sensitivity analysis 134](#_Toc97015327)

[Supplement Figure 158: DAS 28 vs. SDAI, remission, tofacitinib vs. adalimumab (combined with MTX), sensitivity analysis 135](#_Toc97015328)

[Supplement Figure 159: SDAI vs. CDAI, remission, tofacitinib vs. adalimumab (combined with MTX), sensitivity analysis 135](#_Toc97015329)

[Supplement Figure 160: DAS 28 vs. Boolean approach, remission, tofacitinib vs. adalimumab (combined with MTX), sensitivity analysis 135](#_Toc97015330)

[Supplement Figure 161: SDAI vs. Boolean approach, remission, tofacitinib vs. adalimumab (combined with MTX), sensitivity analysis 136](#_Toc97015331)

[Supplement Figure 162: CDAI vs. Boolean approach, remission, tofacitinib vs. adalimumab (combined with MTX), sensitivity analysis 136](#_Toc97015332)

# Details on statistical methods

For the main analysis, confidence intervals for RORs were calculated taking data dependency into account, as the paired OR estimates originated from the same studies. Since paired patient-level data for estimating the correlation were not available, the correlations between the two logORs for each comparison of composite measures were estimated empirically across all treatment comparisons and subsequently applied to estimate the corresponding variance of the RORs. Estimations of correlations were conducted separately for studies on MTX-naïve patients, on patients after MTX failure, and on patients after biologic failure based on the data available from the systematic review conducted by IQWiG. For studies on patients with MTX intolerance, a separate estimation of the correlation was not feasible due to the small number of studies available. Thus, correlations for patients after MTX failure were applied to these studies, as the treatment context for these patients is similar. For studies including patients after MTX failure as well as patients after biologic failure, the mean value of the correlations for both populations was applied. Due to the fact, that the newly approved JAK inhibitors and the IL-6 inhibitor sarilumab were added to the study pool later, the correlation for the 7 corresponding studies was estimated based on the data available from the systematic review conducted by IQWiG.

# Main analyses considering data dependency

## Tabular results

### Biologics vs. placebo

Supplement Table 1: Results on RORs for assessment of low disease activity using the DAS 28 < 3.2, SDAI ≤ 11 or CDAI ≤ 10 in MTX-naïve patients, patients after MTX failure and patients after biologic failure, main analysis

| Biologics versus placebo; in combination with MTX (Number of studies) | Ratio of odds ratios (95% CI) | p value | Heterogeneity among studies  p value / I² (%) | Heterogeneity among study pools  p value / I² (%) |
| --- | --- | --- | --- | --- |
| DAS 28 < 3.2 vs. CDAI ≤ 10 | | | | |
| Abatacept (8) | 1.16 (1.00 to 1.35) | 0.051 | 0.347 / 10.6 | 0.479 / 0 |
| Adalimumab (12) | **1.10 (1.00 to 1.22)** | **0.045** | 0.448 / 0 | 0.895 / 0 |
| Anakinra (2)* | **1.42 (1.08 to 1.87)** | **0.012** | 0.456 / 0 | not applicable |
| Certolizumab pegol (6) | 1.01 (0.85 to 1.19) | 0.952 | 0.789 / 0 | 0.606 / 0 |
| Etanercept (3)† | 0.96 (0.78 to 1.18) | 0.691 | 0.170 / 40.2 | 0.863 / 0 |
| Golimumab (5) | 1.03 (0.83 to 1.29) | 0.777 | 0.686 / 0 | 0.643 / 0 |
| Infliximab (1)* | 0.65 (0.35 to 1.22) | 0.181 | not applicable | not applicable |
| Rituximab (2)‡ | 1.38 (0.61 to 3.11) | 0.441 | 0.355 / 0 | not applicable |
| Tocilizumab (9) | **2.90 (2.50 to 3.38)** | **< 0.001** | 0.220 / 21.5 | 0.490 / 0 |
| DAS 28 < 3.2 vs. SDAI ≤ 11 | | | | |
| Abatacept (8) | 1.06 (0.92 to 1.21) | 0.416 | 0.826 / 0 | 0.619 / 0 |
| Adalimumab (12) | 1.07 (0.98 to 1.16) | 0.142 | 0.348 / 9.8 | 0.868 / 0 |
| Anakinra (2)* | 1.27 (0.97 to 1.67) | 0.080 | 0.847 / 0 | not applicable |
| Certolizumab pegol (6) | 1.03 (0.89 to 1.19) | 0.719 | 0.742 / 0 | 0.522 / 0 |
| Etanercept (3)† | 0.97 (0.81 to 1.16) | 0.737 | 0.064 / 58.6 | 0.704 / 0 |
| Golimumab (5) | 0.93 (0.76 to 1.14) | 0.495 | 0.285 / 20.4 | 0.446 / 0 |
| Infliximab (1)* | 0.57 (0.30 to 1.08) | 0.083 | not applicable | not applicable |
| Rituximab (2)‡ | 1.37 (0.64 to 2.93) | 0.423 | 0.320 / 0 | not applicable |
| Tocilizumab (9) | **2.54 (2.22 to 2.91)** | **< 0.001** | 0.110 / 33.1 | 0.185 / 40.7 |
| SDAI ≤ 11 vs. CDAI ≤ 10 | | | | |
| Abatacept (8) | -§ | | < 0.001 / 82.9 | 0.014 / 76.7 |
| MTX-naïve (2) | 1.00 (0.92 to 1.09) | 0.985 | 0.564 / 0 | not applicable |
| after MTX failure (5) | -§ | | < 0.001 / 89.3 | not applicable |
| after biologic failure (2) | 1.25 (0.98 to 1.59) | 0.074 | 0.438 / 0 | not applicable |
| Adalimumab (12) | -§ | | 0.038 / 45.4 | 0.962 / 0 |
| MTX-naïve (5) | 1.04 (0.99 to 1.09) | 0.150 | 0.670 / 0 | not applicable |
| after MTX failure (7) | -§ | | 0.003 / 69.3 | not applicable |
| after biologic failure (1) | 1.00 (0.64 to 1.56) | 1.000 | not applicable | not applicable |
| Anakinra (2)* | **1.12 (1.02 to 1.23)** | **0.017** | 0.084 / 66.6 | not applicable |
| Certolizumab pegol (6) | 0.98 (0.93 to 1.04) | 0.595 | 0.934 / 0 | 0.976 / 0 |
| Etanercept (3)† | 1.00 (0.92 to 1.08) | 0.947 | 0.958 / 0 | 0.608 / 0 |
| Golimumab (5) | **1.08 (1.01 to 1.16)** | **0.035** | 0.137 / 42.7 | 0.588 / 0 |
| Infliximab (1)* | 1.15 (0.91 to 1.46) | 0.244 | not applicable | not applicable |
| Rituximab (2)‡ | 1.01 (0.88 to 1.15) | 0.941 | 0.987 / 0 | not applicable |
| Tocilizumab (9) | **1.10 (1.05 to 1.15)** | **< 0.001** | 0.119 / 32.1 | 0.068 / 62.9 |
| CDAI=clinical disease activity index; DAS 28=disease activity score 28; MTX=methotrexate; SDAI=simplified disease activity index. Effects shown in bold font indicate a statistically significant ratio of odds ratios. *Includes only studies on patients after MTX failure. †Includes only studies on MTX-naïve patients and patients after MTX failure. ‡Includes only studies on patients after biologic failure. §Effect not interpretable due to heterogeneity among studies and / or study pools (p < 0.05). | | | | |

Supplement Table 2: Results on RORs for assessment of remission using the DAS 28 < 2.6, SDAI ≤ 3.3, CDAI ≤ 2.8 or Boolean approach in MTX-naïve patients, patients after MTX failure and patients after biologic failure, main analysis

| Biologics versus placebo; in combination with MTX (Number of studies) | Ratio of odds ratios (95% CI) | p value | Heterogeneity among studies  p value / I² (%) | Heterogeneity among study pools  p value / I² (%) |
| --- | --- | --- | --- | --- |
| DAS 28 < 2.6 vs. CDAI ≤ 2.8 | | | | |
| Abatacept (8) | 1.01 (0.73 to 1.39) | 0.953 | 0.153 / 33.1 | 0.107 / 55.3 |
| Adalimumab (13) | **1.24 (1.04 to 1.49)** | **0.019** | 0.651 / 0 | 0.873 / 0 |
| Anakinra (2)* | **3.40 (1.24 to 9.27)** | **0.017** | 0.810 / 0 | not applicable |
| Certolizumab pegol (6) | 1.06 (0.80 to 1.42) | 0.670 | 0.754 / 0 | 0.387 / 0 |
| Etanercept (3)† | 0.79 (0.51 to 1.22) | 0.284 | 0.675 / 0 | 0.398 / 0 |
| Golimumab (5) | 1.00 (0.62 to 1.61) | 0.994 | 0.699 / 0 | 0.649 / 0 |
| Infliximab (1)* | 0.70 (0.11 to 4.30) | 0.696 | not applicable | not applicable |
| Rituximab (2)‡ | 0.80 (0.21 to 3.03) | 0.744 | 0.879 / 0 | not applicable |
| Tocilizumab (9) | **2.86 (2.16 to 3.78)** | **< 0.001** | 0.554 / 0 | 0.050 / 66.6 |
| DAS 28 < 2.6 vs. SDAI ≤ 3.3 | | | | |
| Abatacept (8) | 0.94 (0.68 to 1.29) | 0.687 | 0.359 / 9.1 | 0.471 / 0 |
| Adalimumab (13) | 1.14 (0.96 to 1.35) | 0.139 | 0.790 / 0 | 0.542 / 0 |
| Anakinra (2)* | **2.82 (1.02 to 7.80)** | **0.046** | 0.943 / 0 | not applicable |
| Certolizumab pegol (6) | 1.13 (0.86 to 1.48) | 0.377 | 0.894 / 0 | 0.674 / 0 |
| Etanercept (3)† | 0.72 (0.47 to 1.11) | 0.136 | 0.834 / 0 | 0.395 / 0 |
| Golimumab (5) | 0.92 (0.59 to 1.42) | 0.700 | 0.704 / 0 | 0.473 / 0 |
| Infliximab (1)* | 0.31 (0.03 to 3.38) | 0.338 | not applicable | not applicable |
| Rituximab (2)‡ | 0.54 (0.12 to 2.51) | 0.436 | 0.567 / 0 | not applicable |
| Tocilizumab (9) | **2.70 (2.08 to 3.51)** | **< 0.001** | 0.751 / 0 | 0.112 / 54.3 |
| SDAI ≤ 3.3 vs. CDAI ≤ 2.8 | | | | |
| Abatacept (8) | 0.99 (0.88 to 1.11) | 0.859 | 0.443 / 0 | 0.203 / 37.3 |
| Adalimumab (13) | -§ | | 0.023 / 48.2 | 0.170 / 43.6 |
| MTX-naïve (5) | 1.06 (0.99 to 1.13) | 0.074 | 0.213 / 31.3 | not applicable |
| after MTX failure (8) | -§ | | 0.028 / 55.4 | not applicable |
| after biologic failure (1) | 1.00 (0.19 to 5.22) | 1.000 | not applicable | not applicable |
| Anakinra (2)* | 1.20 (0.78 to 1.84) | 0.408 | 0.684 / 0 | not applicable |
| Certolizumab pegol (6) | 0.94 (0.86 to 1.02) | 0.160 | 0.656 / 0 | 0.352 / 4.1 |
| Etanercept (3)† | 1.09 (0.94 to 1.27) | 0.241 | 0.464 / 0 | 0.989 / 0 |
| Golimumab (5) | 1.07 (0.90 to 1.28) | 0.436 | 0.515 / 0 | 0.649 / 0 |
| Infliximab (1)* | 2.23 (0.71 to 7.03) | 0.172 | not applicable | not applicable |
| Rituximab (2)‡ | 1.52 (0.56 to 4.15) | 0.416 | 0.531 / 0 | not applicable |
| Tocilizumab (9) | 1.06 (0.96 to 1.17) | 0.240 | 0.598 / 0 | 0.149 / 47.6 |
| DAS 28 < 2.6 vs. Boolean approach | | | | |
| Abatacept (8) | 0.80 (0.54 to 1.20) | 0.279 | 0.390 / 5.4 | 0.077 / 60.9 |
| Adalimumab (13) | 1.02 (0.85 to 1.24) | 0.814 | 0.730 / 0 | 0.872 / 0 |
| Anakinra (2)* | 0.65 (0.13 to 3.18) | 0.595 | 0.813 / 0 | not applicable |
| Certolizumab pegol (6) | **1.35 (1.02 to 1.77)** | **0.033** | 0.810 / 0 | 0.490 / 0 |
| Etanercept (3)† | 1.05 (0.71 to 1.55) | 0.825 | 0.819 / 0 | 0.980 / 0 |
| Golimumab (5) | 0.93 (0.54 to 1.61) | 0.803 | 0.467 / 0 | 0.422 / 0 |
| Infliximab (1)* | 0.50 (0.04 to 6.81) | 0.606 | not applicable | not applicable |
| Rituximab (2)‡ | 2.15 (0.73 to 6.31) | 0.164 | 0.603 / 0 | not applicable |
| Tocilizumab (9) | **2.63 (1.96 to 3.54)** | **< 0.001** | 0.306 / 13.5 | 0.316 / 13.3 |
| SDAI ≤ 3.3 vs. Boolean approach | | | | |
| Abatacept (8) | 0.81 (0.59 to 1.12) | 0.205 | 0.566 / 0 | 0.405 / 0 |
| Adalimumab (13) | 0.89 (0.77 to 1.03) | 0.119 | 0.710 / 0 | 0.205 / 36.9 |
| Anakinra (2)* | **0.23 (0.06 to 0.83)** | **0.024** | 0.723 / 0 | not applicable |
| Certolizumab pegol (6) | 1.20 (0.99 to 1.47) | 0.069 | 0.936 / 0 | 0.869 / 0 |
| Etanercept (3)† | **1.42 (1.01 to 2.01)** | **0.044** | 0.335 / 11.6 | 0.342 / 0 |
| Golimumab (5) | 0.96 (0.62 to 1.47) | 0.833 | 0.267 / 23.1 | 0.915 / 0 |
| Infliximab (1)* | 1.61 (0.17 to 15.41) | 0.678 | not applicable | not applicable |
| Rituximab (2)‡ | 3.12 (0.77 to 12.67) | 0.112 | 0.222 / 32.9 | not applicable |
| Tocilizumab (9) | 1.05 (0.84 to 1.32) | 0.648 | 0.449 / 0 | 0.959 / 0 |
| CDAI ≤ 2.8 vs. Boolean approach | | | | |
| Abatacept (8) | 0.82 (0.59 to 1.14) | 0.227 | 0.474 / 0 | 0.728 / 0 |
| Adalimumab (13) | **0.82 (0.70 to 0.96)** | **0.014** | 0.979 / 0 | 0.536 / 0 |
| Anakinra (2)* | **0.20 (0.05 to 0.73)** | **0.015** | 0.618 / 0 | not applicable |
| Certolizumab pegol (6) | **1.29 (1.04 to 1.60)** | **0.020** | 0.970 / 0 | 0.754 / 0 |
| Etanercept (3)† | 1.31 (0.92 to 1.86) | 0.137 | 0.253 / 26.5 | 0.333 / 0 |
| Golimumab (5) | 0.93 (0.59 to 1.48) | 0.764 | 0.232 / 28.5 | 0.896 / 0 |
| Infliximab (1)* | 0.72 (0.08 to 6.30) | 0.770 | not applicable | not applicable |
| Rituximab (2)‡ | 2.53 (0.78 to 8.20) | 0.121 | 0.458 / 0 | not applicable |
| Tocilizumab (9) | 1.01 (0.79 to 1.28) | 0.953 | 0.418 / 3.0 | 0.704 / 0 |
| CDAI=clinical disease activity index; DAS 28=disease activity score 28; MTX=methotrexate; SDAI=simplified disease activity index. Effects shown in bold font indicate a statistically significant ratio of odds ratios. *Includes only studies on patients after MTX failure. †Includes only studies on MTX-naïve patients and patients after MTX failure. ‡Includes only studies on patients after biologic failure. §Effect not interpretable due to heterogeneity among studies and / or study pools (p < 0.05). | | | | |

### Biologics vs. biologics / JAK inhibitors vs. biologics

Supplement Table 3: Results on RORs for assessment of low disease activity using the DAS 28 < 3.2, SDAI ≤ 11 or CDAI ≤ 10 for direct comparisons of biological medicines among each other or comparisons with JAK inhibitors in patients after MTX failure, biologic failure or patients intolerant to MTX, main analysis

| Comparison between biologics or JAK inhibitors and biologics (Number of studies) | Ratio of odds ratios (95% CI) | p value | Heterogeneity among studies  p value / I² (%) |
| --- | --- | --- | --- |
| DAS 28 < 3.2 vs. CDAI ≤ 10 | | | |
| Sarilumab vs. adalimumab; monotherapy (1)* | **2.07 (1.20 to 3.57)** | **0.009** | not applicable |
| Tocilizumab vs. adalimumab; monotherapy (1)* | **1.83 (1.12 to 2.98)** | **0.016** | not applicable |
| Tocilizumab + MTX vs. adalimumab + MTX (1)† | **2.65 (1.09 to 6.44)** | **0.031** | not applicable |
| Certolizumab pegol + MTX vs. adalimumab + MTX (1)‡ | 0.96 (0.80 to 1.16) | 0.680 | not applicable |
| Baricitinib + MTX vs. adalimumab + MTX (1)‡ | 1.00 (0.82 to 1.21) | 0.987 | not applicable |
| Filgotinib + MTX vs. adalimumab + MTX (1)‡ | 1.06 (0.87 to 1.29) | 0.588 | not applicable |
| Tofacitinib + MTX vs. adalimumab + MTX (2)§ | 0.85 (0.70 to 1.02) | 0.088 | 0.606 / 0 |
| Upadacitinib + MTX vs. adalimumab + MTX (1)‡ | 1.07 (0.89 to 1.29) | 0.476 | not applicable |
| Upadacitinib + MTX vs. abatacept + MTX (1)† | **1.46 (1.03 to 2.08)** | **0.032** | not applicable |
| Abatacept + MTX vs. adalimumab + MTX (1)‡ | 0.96 (0.77 to 1.20) | 0.721 | not applicable |
| DAS 28 < 3.2 vs. SDAI ≤ 11 | | | |
| Sarilumab vs. adalimumab; monotherapy (1)* | **2.14 (1.26 to 3.64)** | **0.005** | not applicable |
| Tocilizumab vs. adalimumab; monotherapy (1)* | **1.82 (1.14 to 2.94)** | **0.013** | not applicable |
| Tocilizumab + MTX vs. adalimumab + MTX (1)† | 1.28 (0.55 to 2.94) | 0.570 | not applicable |
| Certolizumab pegol + MTX vs. adalimumab + MTX (1)‡ | 1.04 (0.87 to 1.25) | 0.679 | not applicable |
| Baricitinib + MTX vs. adalimumab + MTX (1)‡ | 0.99 (0.82 to 1.19) | 0.917 | not applicable |
| Filgotinib + MTX vs. adalimumab + MTX (1)‡ | 0.98 (0.81 to 1.20) | 0.878 | not applicable |
| Tofacitinib + MTX vs. adalimumab + MTX (2)§ | 0.87 (0.73 to 1.04) | 0.115 | 0.481 / 0 |
| Upadacitinib + MTX vs. adalimumab + MTX (1)‡ | 1.04 (0.87 to 1.25) | 0.634 | not applicable |
| Upadacitinib + MTX vs. abatacept + MTX (1)† | **1.38 (1.00 to 1.91)** | **0.049** | not applicable |
| Abatacept + MTX vs. adalimumab + MTX (1)‡ | 0.98 (0.79 to 1.21) | 0.860 | not applicable |
| SDAI ≤ 11 vs. CDAI ≤ 10 | | | |
| Sarilumab vs. adalimumab; monotherapy (1)* | 0.97 (0.82 to 1.14) | 0.687 | not applicable |
| Tocilizumab vs. adalimumab; monotherapy (1)* | 1.00 (0.85 to 1.17) | 0.974 | not applicable |
| Tocilizumab + MTX vs. adalimumab + MTX (1)† | **2.08 (1.69 to 2.56)** | **< 0.001** | not applicable |
| Certolizumab pegol + MTX vs. adalimumab + MTX (1)‡ | **0.93 (0.87 to 0.99)** | **0.016** | not applicable |
| Baricitinib + MTX vs. adalimumab + MTX (1)‡ | 1.01 (0.94 to 1.08) | 0.800 | not applicable |
| Filgotinib + MTX vs. adalimumab + MTX (1)‡ | **1.07 (1.00 to 1.15)** | **0.042** | not applicable |
| Tofacitinib + MTX vs. adalimumab + MTX (2)§ | 0.98 (0.93 to 1.03) | 0.397 | 0.579 / 0 |
| Upadacitinib + MTX vs. adalimumab + MTX (1)‡ | 1.02 (0.96 to 1.09) | 0.462 | not applicable |
| Upadacitinib + MTX vs. abatacept + MTX (1)† | 1.06 (0.98 to 1.14) | 0.131 | not applicable |
| Abatacept + MTX vs. adalimumab + MTX (1)‡ | 0.98 (0.91 to 1.05) | 0.583 | not applicable |
| CDAI=clinical disease activity index; DAS 28=disease activity score 28; MTX=methotrexate; SDAI=simplified disease activity index. Effects shown in bold font indicate a statistically significant ratio of odds ratios. *Includes only studies on patients with MTX intolerance. †Includes only studies on patients after biologic failure. ‡Includes only studies on patients after MTX failure. §Includes only studies on patients after MTX or biologic failure. | | | |

Supplement Table 4: Results on RORs for assessment of remission using the DAS 28 < 2.6, SDAI ≤ 3.3, CDAI ≤ 2.8 or Boolean approach for direct comparisons of biological medicines among each other or comparisons with JAK inhibitors in patients after MTX failure, biologic failure or patients intolerant to MTX, main analysis

| Comparison between biologics or JAK inhibitors and biologics (Number of studies) | Ratio of odds ratios (95% CI) | p value | Heterogeneity among studies  p value / I² (%) |
| --- | --- | --- | --- |
| DAS 28 < 2.6 vs. CDAI ≤ 2.8 | | | |
| Sarilumab vs. adalimumab; monotherapy (1)* | 1.62 (0.43 to 6.14) | 0.478 | not applicable |
| Tocilizumab vs. adalimumab; monotherapy (1)* | **3.45 (1.37 to 9.09)** | **0.009** | not applicable |
| Tocilizumab + MTX vs. adalimumab + MTX (1)† | 0.93 (0.23 to 3.70) | 0.908 | not applicable |
| Certolizumab pegol + MTX vs. adalimumab + MTX (1)‡ | 1.00 (0.73 to 1.36) | 0.982 | not applicable |
| Baricitinib + MTX vs. adalimumab + MTX (1)‡ | 0.79 (0.58 to 1.08) | 0.147 | not applicable |
| Filgotinib + MTX vs. adalimumab + MTX (1)‡ | 0.96 (0.72 to 1.29) | 0.796 | not applicable |
| Tofacitinib + MTX vs. adalimumab + MTX (2)§ | **0.74 (0.57 to 0.95)** | **0.020** | 0.601 / 0 |
| Upadacitinib + MTX vs. adalimumab + MTX (1)‡ | 1.00 (0.73 to 1.37) | 0.999 | not applicable |
| Upadacitinib + MTX vs. abatacept + MTX (1)† | 1.37 (0.96 to 1.95) | 0.082 | not applicable |
| Abatacept + MTX vs. adalimumab + MTX (1)‡ | 0.89 (0.61 to 1.30) | 0.539 | not applicable |
| DAS 28 < 2.6 vs. SDAI ≤ 3.3 | | | |
| Sarilumab vs. adalimumab; monotherapy (1)* | 1.42 (0.40 to 5.04) | 0.586 | not applicable |
| Tocilizumab vs. adalimumab; monotherapy (1)* | 2.27 (0.91 to 5.88) | 0.080 | not applicable |
| Tocilizumab + MTX vs. adalimumab + MTX (1)† | 0.43 (0.08 to 2.22) | 0.318 | not applicable |
| Certolizumab pegol + MTX vs. adalimumab + MTX (1)‡ | 1.06 (0.79 to 1.43) | 0.696 | not applicable |
| Baricitinib + MTX vs. adalimumab + MTX (1)‡ | 0.76 (0.57 to 1.03) | 0.073 | not applicable |
| Filgotinib + MTX vs. adalimumab + MTX (1)‡ | 1.02 (0.77 to 1.34) | 0.887 | not applicable |
| Tofacitinib + MTX vs. adalimumab + MTX (2)§ | 0.82 (0.65 to 1.04) | 0.105 | 0.604 / 0 |
| Upadacitinib + MTX vs. adalimumab + MTX (1)‡ | 0.93 (0.69 to 1.26) | 0.660 | not applicable |
| Upadacitinib + MTX vs. abatacept + MTX (1)† | 1.20 (0.88 to 1.62) | 0.244 | not applicable |
| Abatacept + MTX vs. adalimumab + MTX (1)‡ | 0.94 (0.65 to 1.36) | 0.753 | not applicable |
| SDAI ≤ 3.3 vs. CDAI ≤2.8 | | | |
| Sarilumab vs. adalimumab; monotherapy (1)* | 1.14 (0.66 to 1.97) | 0.641 | not applicable |
| Tocilizumab vs. adalimumab; monotherapy (1)* | **1.54 (1.06 to 2.17)** | **0.020** | not applicable |
| Tocilizumab + MTX vs. adalimumab + MTX (1)† | 2.13 (0.80 to 5.56) | 0.129 | not applicable |
| Certolizumab pegol + MTX vs. adalimumab + MTX (1)‡ | 0.94 (0.84 to 1.06) | 0.360 | not applicable |
| Baricitinib + MTX vs. adalimumab + MTX (1)‡ | 1.04 (0.92 to 1.18) | 0.538 | not applicable |
| Filgotinib + MTX vs. adalimumab + MTX (1)‡ | 0.94 (0.84 to 1.06) | 0.310 | not applicable |
| Tofacitinib + MTX vs. adalimumab + MTX (2)§ | 0.90 (0.80 to 1.02) | 0.091 | 0.912 / 0 |
| Upadacitinib + MTX vs. adalimumab + MTX (1)‡ | 1.07 (0.94 to 1.22) | 0.293 | not applicable |
| Upadacitinib + MTX vs. abatacept + MTX (1)† | 1.14 (0.93 to 1.40) | 0.193 | not applicable |
| Abatacept + MTX vs. adalimumab + MTX (1)‡ | 0.94 (0.81 to 1.10) | 0.453 | not applicable |
| DAS 28 < 2.6 vs. Boolean approach | | | |
| Sarilumab vs. adalimumab; monotherapy (1)* | 2.69 (0.62 to 11.73) | 0.187 | not applicable |
| Tocilizumab vs. adalimumab; monotherapy (1)* | 2.27 (0.81 to 6.25) | 0.117 | not applicable |
| Tocilizumab + MTX vs. adalimumab + MTX (1)† | 0.89 (0.23 to 3.45) | 0.872 | not applicable |
| Certolizumab pegol + MTX vs. adalimumab + MTX (1)‡ | 1.19 (0.84 to 1.69) | 0.321 | not applicable |
| Baricitinib + MTX vs. adalimumab + MTX (1)‡ | 0.83 (0.58 to 1.19) | 0.311 | not applicable |
| Filgotinib + MTX vs. adalimumab + MTX (1)‡ | 0.96 (0.68 to 1.33) | 0.789 | not applicable |
| Tofacitinib + MTX vs. adalimumab + MTX (2)§ | 0.77 (0.58 to 1.04) | 0.086 | 0.644 / 0 |
| Upadacitinib + MTX vs. adalimumab + MTX (1)‡ | 0.93 (0.64 to 1.35) | 0.700 | not applicable |
| Upadacitinib + MTX vs. abatacept + MTX (1)† | 1.38 (0.94 to 2.04) | 0.101 | not applicable |
| Abatacept + MTX vs. adalimumab + MTX (1)‡ | 1.06 (0.65 to 1.72) | 0.811 | not applicable |
| SDAI ≤ 3.3 vs. Boolean approach | | | |
| Sarilumab vs. adalimumab; monotherapy (1)* | 1.89 (0.55 to 6.55) | 0.314 | not applicable |
| Tocilizumab vs. adalimumab; monotherapy (1)* | 1.00 (0.45 to 2.23) | 1.000 | not applicable |
| Tocilizumab + MTX vs. adalimumab + MTX (1)† | 2.08 (0.57 to 7.69) | 0.269 | not applicable |
| Certolizumab pegol + MTX vs. adalimumab + MTX (1)‡ | 1.12 (0.85 to 1.49) | 0.406 | not applicable |
| Baricitinib + MTX vs. adalimumab + MTX (1)‡ | 1.09 (0.81 to 1.46) | 0.582 | not applicable |
| Filgotinib + MTX vs. adalimumab + MTX (1)‡ | 0.94 (0.72 to 1.22) | 0.627 | not applicable |
| Tofacitinib + MTX vs. adalimumab + MTX (2)§ | 0.94 (0.74 to 1.19) | 0.615 | 0.944 / 0 |
| Upadacitinib + MTX vs. adalimumab + MTX (1)‡ | 0.99 (0.74 to 1.35) | 0.971 | not applicable |
| Upadacitinib + MTX vs. abatacept + MTX (1)† | 1.16 (0.85 to 1.57) | 0.349 | not applicable |
| Abatacept + MTX vs. adalimumab + MTX (1)‡ | 1.13 (0.76 to 1.67) | 0.555 | not applicable |
| CDAI ≤ 2.8 vs. Boolean approach | | | |
| Sarilumab vs. adalimumab; monotherapy (1)* | 1.66 (0.46 to 6.00) | 0.439 | not applicable |
| Tocilizumab vs. adalimumab; monotherapy (1)* | 0.65 (0.29 to 1.45) | 0.298 | not applicable |
| Tocilizumab + MTX vs. adalimumab + MTX (1)† | 0.97 (0.27 to 3.45) | 0.962 | not applicable |
| Certolizumab pegol + MTX vs. adalimumab + MTX (1)‡ | 1.19 (0.89 to 1.59) | 0.229 | not applicable |
| Baricitinib + MTX vs. adalimumab + MTX (1)‡ | 1.04 (0.77 to 1.41) | 0.778 | not applicable |
| Filgotinib + MTX vs. adalimumab + MTX (1)‡ | 0.99 (0.76 to 1.30) | 0.960 | not applicable |
| Tofacitinib + MTX vs. adalimumab + MTX (2)§ | 1.05 (0.81 to 1.35) | 0.727 | 0.991 / 0 |
| Upadacitinib + MTX vs. adalimumab + MTX (1)‡ | 0.93 (0.68 to 1.27) | 0.643 | not applicable |
| Upadacitinib + MTX vs. abatacept + MTX (1)† | 1.01 (0.71 to 1.44) | 0.951 | not applicable |
| Abatacept + MTX vs. adalimumab + MTX (1)‡ | 1.19 (0.80 to 1.79) | 0.386 | not applicable |
| CDAI=clinical disease activity index; DAS 28=disease activity score 28; MTX=methotrexate; SDAI=simplified disease activity index. Effects shown in bold font indicate a statistically significant ratio of odds ratios. *Includes only studies on patients with MTX intolerance. †Includes only studies on patients after biologic failure. ‡Includes only studies on patients after MTX failure. §Includes only studies on patients after MTX or biologic failure. | | | |

## Forest plots

Forest plots are shown for comparisons including more than one study.

### Biologics vs. placebo

#### Abatacept vs. placebo

Supplement Figure 1: DAS 28 vs. CDAI, low disease activity, abatacept vs. placebo (combined with MTX), main analysis

Supplement Figure 2: DAS 28 vs. SDAI, low disease activity, abatacept vs. placebo (combined with MTX), main analysis

Supplement Figure 3: SDAI vs. CDAI, low disease activity, abatacept vs. placebo (combined with MTX), main analysis

Supplement Figure 4: DAS 28 vs. CDAI, remission, abatacept vs. placebo (combined with MTX), main analysis

Supplement Figure 5: DAS 28 vs. SDAI, remission, abatacept vs. placebo (combined with MTX), main analysis

Supplement Figure 6: SDAI vs. CDAI, remission, abatacept vs. placebo (combined with MTX), main analysis

Supplement Figure 7: DAS 28 vs. Boolean approach, remission, abatacept vs. placebo (combined with MTX), main analysis

Supplement Figure 8: SDAI vs. Boolean approach, remission, abatacept vs. placebo (combined with MTX), main analysis

Supplement Figure 9: CDAI vs. Boolean approach, remission, abatacept vs. placebo (combined with MTX), main analysis

#### Adalimumab vs. placebo

Supplement Figure 10: DAS 28 vs. CDAI, low disease activity, adalimumab vs. placebo (combined with MTX), main analysis

Supplement Figure 11: DAS 28 vs. SDAI, low disease activity, adalimumab vs. placebo (combined with MTX), main analysis

Supplement Figure 12: SDAI vs. CDAI, low disease activity, adalimumab vs. placebo (combined with MTX), main analysis

Supplement Figure 13: DAS 28 vs. CDAI, remission, adalimumab vs. placebo (combined with MTX), main analysis

Supplement Figure 14: DAS 28 vs. SDAI, remission, adalimumab vs. placebo (combined with MTX), main analysis

Supplement Figure 15: SDAI vs. CDAI, remission, adalimumab vs. placebo (combined with MTX), main analysis

Supplement Figure 16: DAS 28 vs. Boolean approach, remission, adalimumab vs. placebo (combined with MTX), main analysis

Supplement Figure 17: SDAI vs. Boolean approach, remission, adalimumab vs. placebo (combined with MTX), main analysis

Supplement Figure 18: CDAI vs. Boolean approach, remission, adalimumab vs. placebo (combined with MTX), main analysis

#### Anakinra vs. placebo

Supplement Figure 19: DAS 28 vs. CDAI, low disease activity, anakinra vs. placebo (combined with MTX), main analysis

Supplement Figure 20: DAS 28 vs. SDAI, low disease activity, anakinra vs. placebo (combined with MTX), main analysis

Supplement Figure 21: SDAI vs. CDAI, low disease activity, anakinra vs. placebo (combined with MTX), main analysis

Supplement Figure 22: DAS 28 vs. CDAI, remission, anakinra vs. placebo (combined with MTX), main analysis

Supplement Figure 23: DAS 28 vs. SDAI, remission, anakinra vs. placebo (combined with MTX), main analysis

Supplement Figure 24: SDAI vs. CDAI, remission, anakinra vs. placebo (combined with MTX), main analysis

Supplement Figure 25: DAS 28 vs. Boolean approach, remission, anakinra vs. placebo (combined with MTX), main analysis

Supplement Figure 26: SDAI vs. Boolean approach, remission, anakinra vs. placebo (combined with MTX), main analysis

Supplement Figure 27: CDAI vs. Boolean approach, remission, anakinra vs. placebo (combined with MTX), main analysis

#### Certolizumab pegol vs. placebo

Supplement Figure 28: DAS 28 vs. CDAI, low disease activity, certolizumab pegol vs. placebo (combined with MTX), main analysis

Supplement Figure 29: DAS 28 vs. SDAI, low disease activity, certolizumab pegol vs. placebo (combined with MTX), main analysis

Supplement Figure 30: SDAI vs. CDAI, low disease activity, certolizumab pegol vs. placebo (combined with MTX), main analysis

Supplement Figure 31: DAS 28 vs. CDAI, remission, certolizumab pegol vs. placebo (combined with MTX), main analysis

Supplement Figure 32: DAS 28 vs. SDAI, remission, certolizumab pegol vs. placebo (combined with MTX), main analysis

Supplement Figure 33: SDAI vs. CDAI, remission, certolizumab pegol vs. placebo (combined with MTX), main analysis

Supplement Figure 34: DAS 28 vs. Boolean approach, remission, certolizumab pegol vs. placebo (combined with MTX), main analysis

Supplement Figure 35: SDAI vs. Boolean approach, remission, certolizumab pegol vs. placebo (combined with MTX), main analysis

Supplement Figure 36: CDAI vs. Boolean approach, remission, certolizumab pegol vs. placebo (combined with MTX), main analysis

#### Etanercept vs. placebo

Supplement Figure 37: DAS 28 vs. CDAI, low disease activity, etanercept vs. placebo (combined with MTX), main analysis

Supplement Figure 38: DAS 28 vs. SDAI, low disease activity, etanercept vs. placebo (combined with MTX), main analysis

Supplement Figure 39: SDAI vs. CDAI, low disease activity, etanercept vs. placebo (combined with MTX), main analysis

Supplement Figure 40: DAS 28 vs. CDAI, remission, etanercept vs. placebo (combined with MTX), main analysis

Supplement Figure 41: DAS 28 vs. SDAI, remission, etanercept vs. placebo (combined with MTX), main analysis

Supplement Figure 42: SDAI vs. CDAI, remission, etanercept vs. placebo (combined with MTX), main analysis

Supplement Figure 43: DAS 28 vs. Boolean approach, remission, etanercept vs. placebo (combined with MTX), main analysis

Supplement Figure 44: SDAI vs. Boolean approach, remission, etanercept vs. placebo (combined with MTX), main analysis

Supplement Figure 45: CDAI vs. Boolean approach, remission, etanercept vs. placebo (combined with MTX), main analysis

#### Golimumab vs. placebo

Supplement Figure 46: DAS 28 vs. CDAI, low disease activity, golimumab vs. placebo (combined with MTX), main analysis

Supplement Figure 47: DAS 28 vs. SDAI, low disease activity, golimumab vs. placebo (combined with MTX), main analysis

Supplement Figure 48: SDAI vs. CDAI, low disease activity, golimumab vs. placebo (combined with MTX), main analysis

Supplement Figure 49: DAS 28 vs. CDAI, remission, golimumab vs. placebo (combined with MTX), main analysis

Supplement Figure 50: DAS 28 vs. SDAI, remission, golimumab vs. placebo (combined with MTX), main analysis

Supplement Figure 51: SDAI vs. CDAI, remission, golimumab vs. placebo (combined with MTX), main analysis

Supplement Figure 52: DAS 28 vs. Boolean approach, remission, golimumab vs. placebo (combined with MTX), main analysis

Supplement Figure 53: SDAI vs. Boolean approach, remission, golimumab vs. placebo (combined with MTX), main analysis

Supplement Figure 54: CDAI vs. Boolean approach remission, golimumab vs. placebo (combined with MTX), main analysis

#### Rituximab vs. placebo

Supplement Figure 55: DAS 28 vs. CDAI, low disease activity, rituximab vs. placebo (combined with MTX), main analysis

Supplement Figure 56: DAS 28 vs. SDAI, low disease activity, rituximab vs. placebo (combined with MTX), main analysis

Supplement Figure 57: SDAI vs. CDAI, low disease activity, rituximab vs. placebo (combined with MTX), main analysis

Supplement Figure 58: DAS 28 vs. CDAI, remission, rituximab vs. placebo (combined with MTX), main analysis

Supplement Figure 59: DAS 28 vs. SDAI, remission, rituximab vs. placebo (combined with MTX), main analysis

Supplement Figure 60: SDAI vs. CDAI, remission, rituximab vs. placebo (combined with MTX), main analysis

Supplement Figure 61: DAS 28 vs. Boolean approach, remission, rituximab vs. placebo (combined with MTX), main analysis

Supplement Figure 62: SDAI vs. Boolean approach, remission, rituximab vs. placebo (combined with MTX), main analysis

Supplement Figure 63: CDAI vs. Boolean approach, remission, rituximab vs. placebo (combined with MTX), main analysis

#### Tocilizumab vs. placebo

Supplement Figure 64: DAS 28 vs. CDAI, low disease activity, tocilizumab vs. placebo (combined with MTX), main analysis

Supplement Figure 65: DAS 28 vs. SDAI, low disease activity, tocilizumab vs. placebo (combined with MTX), main analysis

Supplement Figure 66: SDAI vs. CDAI, low disease activity, tocilizumab vs. placebo (combined with MTX), main analysis

Supplement Figure 67: DAS 28 vs. CDAI, remission, tocilizumab vs. placebo (combined with MTX), main analysis

Supplement Figure 68: DAS 28 vs. SDAI, remission, tocilizumab vs. placebo (combined with MTX), main analysis

Supplement Figure 69: SDAI vs. CDAI, remission, tocilizumab vs. placebo (combined with MTX), main analysis

Supplement Figure 70: DAS 28 vs. Boolean approach, remission, tocilizumab vs. placebo (combined with MTX), main analysis

Supplement Figure 71: SDAI vs. Boolean approach, remission, tocilizumab vs. placebo (combined with MTX), main analysis

Supplement Figure 72: CDAI vs. Boolean approach, remission, tocilizumab vs. placebo (combined with MTX), main analysis

### JAK inhibitor (tofacitinib) vs. TNFα inhibitor (adalimumab)

Supplement Figure 73: DAS 28 vs. CDAI, low disease activity, tofacitinib vs. adalimumab (combined with MTX), main analysis

Supplement Figure 74: DAS 28 vs. SDAI, low disease activity, tofacitinib vs. adalimumab (combined with MTX), main analysis

Supplement Figure 75: SDAI vs. CDAI, low disease activity, tofacitinib vs. adalimumab (combined with MTX), main analysis

Supplement Figure 76: DAS 28 vs. CDAI, remission, tofacitinib vs. adalimumab (combined with MTX), main analysis

Supplement Figure 77: DAS 28 vs. SDAI, remission, tofacitinib vs. adalimumab (combined with MTX), main analysis

Supplement Figure 78: SDAI vs. CDAI, remission, tofacitinib vs. adalimumab (combined with MTX), main analysis

Supplement Figure 79: DAS 28 vs. Boolean approach, remission, tofacitinib vs. adalimumab (combined with MTX), main analysis

Supplement Figure 80: SDAI vs. Boolean approach, remission, tofacitinib vs. adalimumab (combined with MTX), main analysis

Supplement Figure 81: CDAI vs. Boolean approach, remission, tofacitinib vs. adalimumab (combined with MTX), main analysis

# Sensitivity analyses not considering data dependency

## Summary of results

Supplement Table 5: Overview of results on RORs for assessment of low disease activity and remission using the DAS 28, SDAI, CDAI and the Boolean approach (remission only), placebo-controlled studies

| Biologic versus placebo; in combination with MTX | Low disease activity, ROR (p value) | | | | | Remission, ROR (p value) | | | | | | | |
| --- | --- | --- | --- | --- | --- | --- | --- | --- | --- | --- | --- | --- | --- |
|  | Number of studies | Number of patients | DAS 28  vs.  CDAI | DAS 28 vs.  SDAI | SDAI  vs.  CDAI | Number of studies | Number of patients | DAS 28 vs.  CDAI | DAS 28  vs.  SDAI | SDAI  vs.  CDAI | DAS 28 vs. Boolean approach | SDAI vs. Boolean approach | CDAI vs. Boolean approach |
| **IL-6 inhibitor** | | | | | | | | | | | | | |
| Tocilizumab | 9 | 3307 | **2.92 (<0.001)** | **2.62 (<0.001)** | 1.12 (0.391) | 9 | 3272 | **2.80 (<0.001)** | **2.60 (<0.001)** | 1.07 (0.748) | **2.59 (<0.001)** | 1.03 (0.905) | 0.96 (0.861) |
| **TNFα inhibitor** | | | | | | | | | | | | | |
| Adalimumab | 12 | 4520 | 1.11 (0.297) | 1.06 (0.522) | 1.04 (0.677) | 13 | 4640 | 1.23 (0.117) | 1.13 (0.354) | 1.09 (0.541) | 1.02  (0.894) | 0.90 (0.497) | 0.83 (0.218) |
| Certolizumab pegol | 6 | 2445 | 1.01 (0.935) | 1.03 (0.869) | 0.98 (0.914) | 6 | 2458 | 1.07 (0.762) | 1.13 (0.568) | 0.94 (0.776) | 1.37  (0.148) | 1.21 (0.373) | 1.29 (0.236) |
| Etanercept | 3* | 877 | 0.96 (0.847) | 0.97 (0.877) | 0.99 (0.975) | 3* | 877 | 0.79 (0.425) | 0.71 (0.280) | 1.10 (0.805) | 1.04 (0.891) | 1.45 (0.309) | 1.33 (0.426) |
| Golimumab | 5 | 987 | 1.04 (0.864) | 0.95 (0.824) | 1.09 (0.706) | 5 | 987 | 1.00 (0.999) | 0.91 (0.787) | 1.09 (0.835) | 0.86 (0.719) | 0.88 (0.777) | 0.86 (0.733) |
| Infliximab | 1† | 171 | 0.65 (0.514) | 0.57 (0.404) | 1.15 (0.842) | 1† | 171 | 0.70 (0.779) | 0.31 (0.476) | 2.23 (0.666) | 0.50 (0.680) | 1.61 (0.822) | 0.72 (0.864) |
| **IL-1 receptor antagonist** | | | | | | | | | | | | | |
| Anakinra | 2† | 1035 | 1.43 (0.220) | 1.27 (0.406) | 1.13 (0.688) | 2† | 1035 | 3.41 (0.071) | 2.83 (0.141) | 1.20 (0.836) | 0.64  (0.644) | 0.23 (0.185) | 0.19 (0.136) |
| **T-cell activation inhibitor** | | | | | | | | | | | | | |
| Abatacept | 8 | 2363 | 1.15 (0.351) | 1.05 (0.746) | 1.10 (0.520) | 8 | 2364 | 1.00 (0.986) | 0.94 (0.799) | 1.04 (0.891) | 0.81  (0.473) | 0.81 (0.519) | 0.81 (0.492) |
| **B-cell depleting agent** | | | | | | | | | | | | | |
| Rituximab | 2‡ | 528 | 1.39 (0.591) | 1.38 (0.596) | 1.01 (0.992) | 2‡ | 534 | 0.80 (0.846) | 0.49 (0.615) | 1.62 (0.749) | 2.15  (0.446) | 3.91 (0.336) | 2.58 (0.420) |
| The thresholds used for the assessments were: DAS 28 < 3.2, SDAI ≤ 11 and CDAI ≤ 10 for low disease activity, DAS 28 < 2.6, SDAI ≤ 3.3, CDAI ≤ 2.8 and Boolean approach (≤ 1 swollen joint, ≤ 1 tender joint, C-reactive protein ≤ 1 mg/dl and global assessment of disease activity by the patient ≤ 1 on a scale from 0 to 10) for remission. Statistically significant RORs are shown in bold font. 95% CIs are shown in Supplement Tables 7 and 8. * Includes only studies on MTX-naïve patients and patients after MTX failure. † Includes only studies on patients after MTX failure. § Includes only studies on patients after biologic failure. CDAI=clinical disease activity index; DAS 28=disease activity score 28; MTX=methotrexate; RORs= ratio of odds ratios; SDAI=simplified disease activity index. | | | | | | | | | | | | | |

Supplement Table 6: Overview of results on RORs for assessment of low disease activity and remission using the DAS 28, SDAI, CDAI and the Boolean approach (remission only), active-controlled studies

| Treatment comparison | Low disease activity, ROR (p value) | | | | | Remission, ROR (p value) | | | | | | | |
| --- | --- | --- | --- | --- | --- | --- | --- | --- | --- | --- | --- | --- | --- |
|  | Number of studies | Number of patients | DAS 28 vs. CDAI | DAS 28 vs. SDAI | SDAI vs. CDAI | Number of studies | Number of patients | DAS 28  vs.  CDAI | DAS 28 vs.  SDAI | SDAI  vs.  CDAI | DAS 28 vs. Boolean approach | SDAI vs. Boolean approach | CDAI vs. Boolean approach |
| **IL-6 inhibitor vs. TNFα inhibitor** | | | | | | | | | | | | | |
| Sarilumab vs. adalimumab; monotherapy | 1* | 169 | 2.07 (0.193) | 2.14 (0.171) | 0.97 (0.947) | 1* | 169 | 1.62 (0.614) | 1.42 (0.711) | 1.14 (0.908) | 2.69 (0.320) | 1.89 (0.585) | 1.66 (0.665) |
| Tocilizumab vs. adalimumab; monotherapy | 1* | 131 | 1.83 (0.241) | 1.82 (0.242) | 1.00 (0.996) | 1* | 131 | 3.45 (0.078) | 2.27 (0.261) | 1.54 (0.560) | 2.27 (0.261) | 1.00 (1.000) | 0.65 (0.560) |
| Tocilizumab + MTX vs. adalimumab + MTX | 1† | 69 | 2.65 (0.165) | 1.28 (0.735) | 2.08 (0.304) | 1† | 69 | 0.93 (0.940) | 0.43 (0.516) | 2.13 (0.618) | 0.89 (0.918) | 2.08 (0.632) | 0.97 (0.982) |
| **TNFα inhibitor vs. TNFα inhibitor** | | | | | | | | | | | | | |
| Certolizumab pegol + MTX vs. adalimumab + MTX | 1‡ | 836 | 0.96 (0.842) | 1.04 (0.845) | 0.93 (0.694) | 1‡ | 836 | 1.00 (0.988) | 1.06 (0.801) | 0.94 (0.821) | 1.19 (0.473) | 1.12 (0.651) | 1.19 (0.499) |
| **JAK inhibitor vs. TNFα inhibitor** | | | | | | | | | | | | | |
| Baricitinib + MTX vs. adalimumab + MTX | 1‡ | 817 | 1.00 (0.994) | 0.99 (0.961) | 1.01 (0.967) | 1‡ | 817 | 0.79 (0.321) | 0.76 (0.244) | 1.04 (0.879) | 0.83 (0.457) | 1.09 (0.763) | 1.04 (0.874) |
| Filgotinib + MTX vs. adalimumab + MTX | 1‡ | 800 | 1.06 (0.793) | 0.98 (0.942) | 1.07 (0.739) | 1‡ | 800 | 0.96 (0.861) | 1.02 (0.927) | 0.94 (0.802) | 0.96 (0.846) | 0.94 (0.791) | 0.99 (0.978) |
| Tofacitinib + MTX vs. adalimumab + MTX | 2§ | 1152 | 0.85 (0.331) | 0.87 (0.397) | 0.98 (0.898) | 2§ | 1152 | 0.74 (0.143) | 0.82 (0.352) | 0.90 (0.646) | 0.77 (0.255) | 0.94 (0.807) | 1.05 (0.854) |
| Upadacitinib + MTX vs. adalimumab + MTX | 1‡ | 978 | 1.07 (0.730) | 1.04 (0.822) | 1.02 (0.904) | 1‡ | 978 | 1.00 (1.000) | 0.93 (0.774) | 1.07 (0.795) | 0.93 (0.776) | 0.99 (0.984) | 0.93 (0.794) |
| **JAK inhibitor vs. T-cell activation inhibitor** | | | | | | | | | | | | | |
| Upadacitinib + MTX vs. abatacept + MTX | 1† | 438 | 1.46 (0.167) | 1.38 (0.241) | 1.06 (0.835) | 1† | 438 | 1.37 (0.320) | 1.20 (0.574) | 1.14 (0.702) | 1.38 (0.341) | 1.16 (0.698) | 1.01 (0.976) |
| **T-cell activation inhibitor vs. TNFα inhibitor** | | | | | | | | | | | | | |
| Abatacept + MTX vs. adalimumab + MTX | 1‡ | 606 | 0.96 (0.863) | 0.98 (0.934) | 0.98 (0.928) | 1‡ | 606 | 0.89 (0.673) | 0.94 (0.836) | 0.94 (0.853) | 1.06 (0.857) | 1.13 (0.744) | 1.19 (0.619) |
| The thresholds used for the assessments were: DAS 28 < 3.2, SDAI ≤ 11 and CDAI ≤ 10 for low disease activity, DAS 28 < 2.6, SDAI ≤ 3.3, CDAI ≤ 2.8 and Boolean approach (≤ 1 swollen joint, ≤ 1 tender joint, C-reactive protein ≤ 1 mg/dl and global assessment of disease activity by the patient ≤ 1 on a scale from 0 to 10) for remission. 95% CIs are shown in Supplement Tables 9 and 10. *Includes only studies on patients with MTX intolerance. † Includes only studies on patients after biologic failure. ‡ Includes only studies on patients after MTX failure. §Includes only studies on patients after MTX or biologic failure. CDAI=clinical disease activity index; DAS 28=disease activity score 28; MTX=methotrexate; RORs=ratio of odds ratios, SDAI=simplified disease activity index. | | | | | | | | | | | | | |

## Tabular results

### Biologics vs. placebo

Supplement Table 7: Results on RORs for assessment of low disease activity using the DAS 28 < 3.2, SDAI ≤ 11 or CDAI ≤ 10 in MTX-naïve patients, patients after MTX failure and patients after biologic failure, sensitivity analysis

| Biologics versus placebo; in combination with MTX (Number of studies) | Ratio of odds ratios (95% CI) | p value | Heterogeneity among studies  p value / I² (%) | Heterogeneity among study pools  p value / I² (%) |
| --- | --- | --- | --- | --- |
| DAS 28 < 3.2 vs. CDAI ≤ 10 | | | | |
| Abatacept (8) | 1.15 (0.86 to 1.53) | 0.351 | 0.972 / 0 | 0.796 / 0 |
| Adalimumab (12) | 1.11 (0.92 to 1.33) | 0.297 | 0.995 / 0 | 0.962 / 0 |
| Anakinra (2)* | 1.43 (0.81 to 2.51) | 0.220 | 0.710 / 0 | not applicable |
| Certolizumab pegol (6) | 1.01 (0.73 to 1.40) | 0.935 | 0.968 / 0 | 0.831 / 0 |
| Etanercept (3)† | 0.96 (0.64 to 1.43) | 0.847 | 0.697 / 0 | 0.932 / 0 |
| Golimumab (5) | 1.04 (0.67 to 1.60) | 0.864 | 0.960 / 0 | 0.861 / 0 |
| Infliximab (1)* | 0.65 (0.18 to 2.37) | 0.514 | not applicable | not applicable |
| Rituximab (2)‡ | 1.39 (0.42 to 4.63) | 0.591 | 0.549 / 0 | not applicable |
| Tocilizumab (9) | **2.92 (2.20 to 3.87)** | **< 0.001** | 0.974 / 0 | 0.784 / 0 |
| DAS 28 < 3.2 vs. SDAI ≤ 11 | | | | |
| Abatacept (8) | 1.05 (0.78 to 1.41) | 0.746 | 0.998 / 0 | 0.844 / 0 |
| Adalimumab (12) | 1.06 (0.88 to 1.28) | 0.522 | 0.996 / 0 | 0.962 / 0 |
| Anakinra (2)* | 1.27 (0.72 to 2.26) | 0.406 | 0.926 / 0 | not applicable |
| Certolizumab pegol (6) | 1.03 (0.74 to 1.42) | 0.869 | 0.965 / 0 | 0.821 / 0 |
| Etanercept (3)† | 0.97 (0.65 to 1.45) | 0.877 | 0.698 / 0 | 0.860 / 0 |
| Golimumab (5) | 0.95 (0.62 to 1.47) | 0.824 | 0.878 / 0 | 0.802 / 0 |
| Infliximab (1)* | 0.57 (0.15 to 2.16) | 0.404 | not applicable | not applicable |
| Rituximab (2)‡ | 1.38 (0.42 to 4.61) | 0.596 | 0.550 / 0 | not applicable |
| Tocilizumab (9) | **2.62 (1.98 to 3.47)** | **< 0.001** | 0.971 / 0 | 0.654 / 0 |
| SDAI ≤ 11 vs. CDAI ≤ 10 | | | | |
| Abatacept (8) | 1.10 (0.83 to 1.46) | 0.520 | 0.994 / 0 | 0.837 / 0 |
| MTX-naïve (2) | 1.00 (0.66 to 1.52) | 0.997 | 0.907 / 0 | not applicable |
| after MTX failure (5) | 1.18 (0.79 to 1.77) | 0.409 | 0.900 / 0 | not applicable |
| after biologic failure (2) | 1.26 (0.28 to 5.76) | 0.766 | 0.913 / 0 | not applicable |
| Adalimumab (12) | 1.04 (0.86 to 1.25) | 0.677 | 1.000 / 0 | 0.999 / 0 |
| MTX-naïve (5) | 1.04 (0.81 to 1.33) | 0.770 | 0.999 / 0 | not applicable |
| after MTX failure (7) | 1.04 (0.78 to 1.39) | 0.765 | 0.997 / 0 | not applicable |
| after biologic failure (1) | 1.00 (0.04 to 25.41) | 1.000 | not applicable | not applicable |
| Anakinra (2)* | 1.13 (0.63 to 2.00) | 0.688 | 0.768 / 0 | not applicable |
| Certolizumab pegol (6) | 0.98 (0.72 to 1.34) | 0.914 | 1.000 / 0 | 0.999 / 0 |
| Etanercept (3)† | 0.99 (0.66 to 1.49) | 0.975 | 1.000 / 0 | 0.928 / 0 |
| Golimumab (5) | 1.09 (0.71 to 1.67) | 0.706 | 0.996 / 0 | 0.989 / 0 |
| Infliximab (1)* | 1.15 (0.29 to 4.65) | 0.842 | not applicable | not applicable |
| Rituximab (2)‡ | 1.01 (0.39 to 2.61) | 0.992 | 0.998 / 0 | not applicable |
| Tocilizumab (9) | 1.12 (0.87 to 1.44) | 0.391 | 1.000 / 0 | 0.923 / 0 |
| CDAI=clinical disease activity index; DAS 28=disease activity score 28; MTX=methotrexate; SDAI=simplified disease activity index. Effects shown in bold font indicate a statistically significant ratio of odds ratios. *Includes only studies on patients after MTX failure. †Includes only studies on MTX-naïve patients and patients after MTX failure. ‡Includes only studies on patients after biologic failure. | | | | |

Supplement Table 8: Results on RORs for assessment of remission using the DAS 28 < 2.6, SDAI ≤ 3.3, CDAI ≤ 2.8 or Boolean definition in MTX-naïve patients, patients after MTX failure and patients after biologic failure, sensitivity analysis

| Biologics versus placebo; in combination with MTX (Number of studies) | Ratio of odds ratios (95% CI) | p value | Heterogeneity among studies  p value / I² (%) | Heterogeneity among study pools  p value / I² (%) |
| --- | --- | --- | --- | --- |
| DAS 28 < 2.6 vs. CDAI ≤ 2.8 | | | | |
| Abatacept (8) | 1.00 (0.63 to 1.59) | 0.986 | 0.635 / 0 | 0.323 / 11.6 |
| Adalimumab (13) | 1.23 (0.95 to 1.59) | 0.117 | 0.952 / 0 | 0.895 / 0 |
| Anakinra (2)* | 3.41 (0.90 to 12.96) | 0.071 | 0.861 / 0 | not applicable |
| Certolizumab pegol (6) | 1.07 (0.70 to 1.61) | 0.762 | 0.959 / 0 | 0.671 / 0 |
| Etanercept (3)† | 0.79 (0.43 to 1.42) | 0.425 | 0.829 / 0 | 0.537 / 0 |
| Golimumab (5) | 1.00 (0.50 to 1.98) | 0.999 | 0.882 / 0 | 0.808 / 0 |
| Infliximab (1)* | 0.70 (0.05 to 8.84) | 0.779 | not applicable | not applicable |
| Rituximab (2)‡ | 0.80 (0.08 to 7.78) | 0.846 | 0.931 / 0 | not applicable |
| Tocilizumab (9) | **2.80 (1.86 to 4.21)** | **< 0.001** | 0.971 / 0 | 0.264 / 24.9 |
| DAS 28 < 2.6 vs. SDAI ≤ 3.3 | | | | |
| Abatacept (8) | 0.94 (0.59 to 1.51) | 0.799 | 0.816 / 0 | 0.689 / 0 |
| Adalimumab (13) | 1.13 (0.87 to 1.46) | 0.354 | 0.985 / 0 | 0.723 / 0 |
| Anakinra (2)* | 2.83 (0.71 to 11.26) | 0.141 | 0.960 / 0 | not applicable |
| Certolizumab pegol (6) | 1.13 (0.75 to 1.70) | 0.568 | 0.988 / 0 | 0.851 / 0 |
| Etanercept (3)† | 0.71 (0.39 to 1.32) | 0.280 | 0.928 / 0 | 0.537 / 0 |
| Golimumab (5) | 0.91 (0.46 to 1.80) | 0.787 | 0.895 / 0 | 0.702 / 0 |
| Infliximab (1)* | 0.31 (0.01 to 7.65) | 0.476 | not applicable | not applicable |
| Rituximab (2)‡ | 0.49 (0.03 to 7.79) | 0.615 | 0.776 / 0 | not applicable |
| Tocilizumab (9) | **2.60 (1.73 to 3.92)** | **< 0.001** | 0.998 / 0 | 0.466 / 0 |
| SDAI ≤ 3.3 vs. CDAI ≤ 2.8 | | | | |
| Abatacept (8) | 1.04 (0.61 to 1.76) | 0.891 | 0.999 / 0 | 0.853 / 0 |
| Adalimumab (13) | 1.09 (0.82 to 1.45) | 0.541 | 1.000 / 0 | 0.937 / 0 |
| MTX-naïve (5) | 1.06 (0.77 to 1.47) | 0.720 | 0.994 / 0 | not applicable |
| after MTX failure (8) | 1.20 (0.67 to 2.15) | 0.540 | 0.983 / 0 | not applicable |
| after biologic failure (1) | 1.00 (0.00 to 279.74) | 1.000 | not applicable | not applicable |
| Anakinra (2)* | 1.20 (0.21 to 6.74) | 0.836 | 0.920 / 0 | not applicable |
| Certolizumab pegol (6) | 0.94 (0.62 to 1.42) | 0.776 | 0.999 / 0 | 0.902 / 0 |
| Etanercept (3)† | 1.10 (0.53 to 2.26) | 0.805 | 0.984 / 0 | 0.996 / 0 |
| Golimumab (5) | 1.09 (0.50 to 2.37) | 0.835 | 0.996 / 0 | 0.977 / 0 |
| Infliximab (1)* | 2.23 (0.06 to 83.99) | 0.666 | not applicable | not applicable |
| Rituximab (2)‡ | 1.62 (0.08 to 31.03) | 0.749 | 0.843 / 0 | not applicable |
| Tocilizumab (9) | 1.07 (0.70 to 1.65) | 0.748 | 1.000 / 0 | 0.898 / 0 |
| DAS 28 < 2.6 vs. Boolean definition | | | | |
| Abatacept (8) | 0.81 (0.46 to 1.43) | 0.473 | 0.803 / 0 | 0.277 / 22.2 |
| Adalimumab (13) | 1.02 (0.77 to 1.35) | 0.894 | 0.967 / 0 | 0.967 / 0 |
| Anakinra (2)* | 0.64 (0.09 to 4.29) | 0.644 | 0.850 / 0 | not applicable |
| Certolizumab pegol (6) | 1.37 (0.90 to 2.08) | 0.148 | 0.959 / 0 | 0.701 / 0 |
| Etanercept (3)† | 1.04 (0.59 to 1.85) | 0.891 | 0.930 / 0 | 0.982 / 0 |
| Golimumab (5) | 0.86 (0.39 to 1.93) | 0.719 | 0.672 / 0 | 0.617 / 0 |
| Infliximab (1)* | 0.50 (0.02 to 13.13) | 0.680 | not applicable | not applicable |
| Rituximab (2)‡ | 2.15 (0.30 to 15.37) | 0.446 | 0.776 / 0 | not applicable |
| Tocilizumab (9) | **2.59 (1.65 to 4.04)** | **< 0.001** | 0.903 / 0 | 0.618 / 0 |
| SDAI ≤ 3.3 vs. Boolean definition | | | | |
| Abatacept (8) | 0.81 (0.44 to 1.52) | 0.519 | 0.983 / 0 | 0.791 / 0 |
| Adalimumab (13) | 0.90 (0.66 to 1.22) | 0.497 | 0.998 / 0 | 0.688 / 0 |
| Anakinra (2)* | 0.23 (0.03 to 2.04) | 0.185 | 0.842 / 0 | not applicable |
| Certolizumab pegol (6) | 1.21 (0.80 to 1.84) | 0.373 | 0.998 / 0 | 0.964 / 0 |
| Etanercept (3)† | 1.45 (0.71 to 2.96) | 0.309 | 0.829 / 0 | 0.597 / 0 |
| Golimumab (5) | 0.88 (0.36 to 2.13) | 0.777 | 0.721 / 0 | 0.937 / 0 |
| Infliximab (1)* | 1.61 (0.03 to 103.84) | 0.822 | not applicable | not applicable |
| Rituximab (2)‡ | 3.91 (0.24 to 63.23) | 0.336 | 0.584 / 0 | not applicable |
| Tocilizumab (9) | 1.03 (0.65 to 1.63) | 0.905 | 0.996 / 0 | 0.988 / 0 |
| CDAI ≤ 2.8 vs. Boolean definition | | | | |
| Abatacept (8) | 0.81 (0.44 to 1.49) | 0.492 | 0.964 / 0 | 0.932 / 0 |
| Adalimumab (13) | 0.83 (0.61 to 1.12) | 0.218 | 1.000 / 0 | 0.842 / 0 |
| Anakinra (2)* | 0.19 (0.02 to 1.68) | 0.136 | 0.773 / 0 | not applicable |
| Certolizumab pegol (6) | 1.29 (0.85 to 1.97) | 0.236 | 0.999 / 0 | 0.931 / 0 |
| Etanercept (3)† | 1.33 (0.66 to 2.66) | 0.426 | 0.748 / 0 | 0.575 / 0 |
| Golimumab (5) | 0.86 (0.35 to 2.08) | 0.733 | 0.648 / 0 | 0.910 / 0 |
| Infliximab (1)* | 0.72 (0.02 to 28.84) | 0.864 | not applicable | not applicable |
| Rituximab (2)‡ | 2.58 (0.26 to 25.97) | 0.420 | 0.716 / 0 | not applicable |
| Tocilizumab (9) | 0.96 (0.60 to 1.53) | 0.861 | 0.988 / 0 | 0.894 / 0 |
| CDAI=clinical disease activity index; DAS 28=disease activity score 28; MTX=methotrexate; SDAI=simplified disease activity index. Effects shown in bold font indicate a statistically significant ratio of odds ratios. *Includes only studies on patients after MTX failure. †Includes only studies on MTX-naïve patients and patients after MTX failure. ‡Includes only studies on patients after biologic failure. | | | | |

### Biologics vs. biologics / JAK inhibitors vs. biologics

Supplement Table 9: Results on RORs for assessment of low disease activity using the DAS 28 < 3.2, SDAI ≤ 11 or CDAI ≤ 10 for direct comparisons of biological medicines among each other or comparisons with JAK inhibitors in patients after MTX failure, biologic failure or patients intolerant to MTX, sensitivity analysis

| Comparison between biologics or JAK inhibitors and biologics (Number of studies) | Ratio of odds ratios (95% CI) | p value | Heterogeneity among studies  p value / I² (%) |
| --- | --- | --- | --- |
| DAS 28 < 3.2 vs. CDAI ≤ 10 | | | |
| Sarilumab vs. adalimumab; monotherapy (1)* | 2.07 (0.69 to 6.20) | 0.193 | not applicable |
| Tocilizumab vs. Aadalimumab; monotherapy (1)* | 1.83 (0.67 to 5.00) | 0.241 | not applicable |
| Tocilizumab + MTX vs. adalimumab + MTX (1)† | 2.65 (0.67 to 10.00) | 0.165 | not applicable |
| Certolizumab pegol + MTX vs. adalimumab + MTX (1)‡ | 0.96 (0.65 to 1.41) | 0.842 | not applicable |
| Baricitinib + MTX vs. adalimumab + MTX (1)‡ | 1.00 (0.67 to 1.48) | 0.994 | not applicable |
| Filgotinib + MTX vs. adalimumab + MTX (1)‡ | 1.06 (0.70 to 1.60) | 0.793 | not applicable |
| Tofacitinib + MTX vs. adalimumab + MTX (2)§ | 0.85 (0.61 to 1.18) | 0.331 | 0.768 / 0 |
| Upadacitinib + MTX vs. adalimumab + MTX (1)‡ | 1.07 (0.73 to 1.57) | 0.730 | not applicable |
| Upadacitinib + MTX vs. abatacept + MTX (1)† | 1.46 (0.85 to 2.52) | 0.167 | not applicable |
| Abatacept + MTX vs. adalimumab + MTX (1)‡ | 0.96 (0.61 to 1.51) | 0.863 | not applicable |
| DAS 28 < 3.2 vs. SDAI ≤ 11 | | | |
| Sarilumab vs. adalimumab; monotherapy (1)* | 2.14 (0.72 to 6.36) | 0.171 | not applicable |
| Tocilizumab vs. adalimumab; monotherapy (1)* | 1.82 (0.67 to 5.00) | 0.242 | not applicable |
| Tocilizumab + MTX vs. adalimumab + MTX (1)† | 1.28 (0.31 to 10.00) | 0.735 | not applicable |
| Certolizumab pegol + MTX vs. adalimumab + MTX (1)‡ | 1.04 (0.71 to 1.52) | 0.845 | not applicable |
| Baricitinib + MTX vs. adalimumab + MTX (1)‡ | 0.99 (0.67 to 1.47) | 0.961 | not applicable |
| Filgotinib + MTX vs. adalimumab + MTX (1)‡ | 0.98 (0.65 to 1.49) | 0.942 | not applicable |
| Tofacitinib + MTX vs. adalimumab + MTX (2)§ | 0.87 (0.63 to 1.21) | 0.397 | 0.705 / 0 |
| Upadacitinib + MTX vs. adalimumab + MTX (1)‡ | 1.04 (0.71 to 1.53) | 0.822 | not applicable |
| Upadacitinib + MTX vs. abatacept + MTX (1)† | 1.38 (0.80 to 2.38) | 0.241 | not applicable |
| Abatacept + MTX vs. adalimumab + MTX (1)‡ | 0.98 (0.63 to 1.54) | 0.934 | not applicable |
| SDAI ≤ 11 vs. CDAI ≤ 10 | | | |
| Sarilumab vs. adalimumab; monotherapy (1)* | 0.97 (0.36 to 2.59) | 0.947 | not applicable |
| Tocilizumab vs. adalimumab; monotherapy (1)* | 1.00 (0.37 to 2.66) | 0.996 | not applicable |
| Tocilizumab + MTX vs. adalimumab + MTX (1)† | 2.08 (0.52 to 8.33) | 0.304 | not applicable |
| Certolizumab pegol + MTX vs. adalimumab + MTX (1)‡ | 0.93 (0.63 to 1.37) | 0.694 | not applicable |
| Baricitinib + MTX vs. adalimumab + MTX (1)‡ | 1.01 (0.68 to 1.50) | 0.967 | not applicable |
| Filgotinib + MTX vs. adalimumab + MTX (1)‡ | 1.07 (0.71 to 1.63) | 0.739 | not applicable |
| Tofacitinib + MTX vs. adalimumab + MTX (2)§ | 0.98 (0.70 to 1.36) | 0.898 | 0.933 / 0 |
| Upadacitinib + MTX vs. adalimumab + MTX (1)‡ | 1.02 (0.70 to 1.50) | 0.904 | not applicable |
| Upadacitinib + MTX vs. abatacept + MTX (1)† | 1.06 (0.62 to 1.81) | 0.835 | not applicable |
| Abatacept + MTX vs. adalimumab + MTX (1)‡ | 0.98 (0.62 to 1.54) | 0.928 | not applicable |
| CDAI=clinical disease activity index; DAS 28=disease activity score 28; MTX=methotrexate; SDAI=simplified disease activity index. Effects shown in bold font indicate a statistically significant ratio of odds ratios. *Includes only studies on patients with MTX intolerance. †Includes only studies on patients after biologic failure. ‡Includes only studies on patients after MTX failure. §Includes only studies on patients after MTX or biologic failure. | | | |

Supplement Table 10: Results on RORs for assessment of remission using the DAS 28 < 2.6, SDAI ≤ 3.3, CDAI ≤ 2.8 or Boolean definition for direct comparisons of biological medicines among each other or comparisons with JAK inhibitors in patients after MTX failure, biologic failure or patients intolerant to MTX, sensitivity analysis

| Comparison between biologics or JAK inhibitors and biologics (Number of studies) | Ratio of odds ratios (95% CI) | p value | Heterogeneity among studies  p value / I² (%) |
| --- | --- | --- | --- |
| DAS 28 < 2.6 vs. CDAI ≤ 2.8 | | | |
| Sarilumab vs. adalimumab; monotherapy (1)* | 1.62 (0.25 to 10.60) | 0.614 | not applicable |
| Tocilizumab vs. adalimumab; monotherapy (1)* | 3.45 (0.87 to 14.29) | 0.078 | not applicable |
| Tocilizumab + MTX vs. adalimumab + MTX (1)† | 0.93 (0.11 to 7.69) | 0.940 | not applicable |
| Certolizumab pegol + MTX vs. adalimumab + MTX (1)‡ | 1.00 (0.63 to 1.57) | 0.988 | not applicable |
| Baricitinib + MTX vs. adalimumab + MTX (1)‡ | 0.79 (0.50 to 1.25) | 0.321 | not applicable |
| Filgotinib + MTX vs. adalimumab + MTX (1)‡ | 0.96 (0.62 to 1.48) | 0.861 | not applicable |
| Tofacitinib + MTX vs. adalimumab + MTX (2)§ | 0.74 (0.49 to 1.11) | 0.143 | 0.740 / 0 |
| Upadacitinib + MTX vs. adalimumab + MTX (1)‡ | 1.00 (0.63 to 1.59) | 1.000 | not applicable |
| Upadacitinib + MTX vs. abatacept + MTX (1)† | 1.37 (0.74 to 2.54) | 0.320 | not applicable |
| Abatacept + MTX vs. adalimumab + MTX (1)‡ | 0.89 (0.51 to 1.54) | 0.673 | not applicable |
| DAS 28 < 2.6 vs. SDAI ≤ 3.3 | | | |
| Sarilumab vs. adalimumab; monotherapy (1)* | 1.42 (0.22 to 9.17) | 0.711 | not applicable |
| Tocilizumab vs. adalimumab; monotherapy (1)* | 2.27 (0.54 to 10.00) | 0.261 | not applicable |
| Tocilizumab + MTX vs. adalimumab + MTX (1)† | 0.43 (0.03 to 5.56) | 0.516 | not applicable |
| Certolizumab pegol + MTX vs. adalimumab + MTX (1)‡ | 1.06 (0.67 to 1.69) | 0.801 | not applicable |
| Baricitinib + MTX vs. adalimumab + MTX (1)‡ | 0.76 (0.48 to 1.20) | 0.244 | not applicable |
| Filgotinib + MTX vs. adalimumab + MTX (1)‡ | 1.02 (0.66 to 1.57) | 0.927 | not applicable |
| Tofacitinib + MTX vs. adalimumab + MTX (2)§ | 0.82 (0.55 to 1.24) | 0.352 | 0.765 / 0 |
| Upadacitinib + MTX vs. adalimumab + MTX (1)‡ | 0.93 (0.59 to 1.49) | 0.774 | not applicable |
| Upadacitinib + MTX vs. abatacept + MTX (1)† | 1.20 (0.64 to 2.24) | 0.574 | not applicable |
| Abatacept + MTX vs. adalimumab + MTX (1)‡ | 0.94 (0.54 to 1.65) | 0.836 | not applicable |
| SDAI ≤ 3.3 vs. CDAI ≤ 2.8 | | | |
| Sarilumab vs. adalimumab; monotherapy (1)* | 1.14 (0.12 to 10.52) | 0.908 | not applicable |
| Tocilizumab vs. adalimumab; monotherapy (1)* | 1.54 (0.36 to 6.25) | 0.560 | not applicable |
| Tocilizumab + MTX vs. adalimumab + MTX (1)† | 2.13 (0.11 to 50.00) | 0.618 | not applicable |
| Certolizumab pegol + MTX vs. adalimumab + MTX (1)‡ | 0.94 (0.58 to 1.54) | 0.821 | not applicable |
| Baricitinib + MTX vs. adalimumab + MTX (1)‡ | 1.04 (0.63 to 1.72) | 0.879 | not applicable |
| Filgotinib + MTX vs. adalimumab + MTX (1)‡ | 0.94 (0.60 to 1.49) | 0.802 | not applicable |
| Tofacitinib + MTX vs. adalimumab + MTX (2)§ | 0.90 (0.57 to 1.41) | 0.646 | 0.976 / 0 |
| Upadacitinib + MTX vs. adalimumab + MTX (1)‡ | 1.07 (0.64 to 1.79) | 0.795 | not applicable |
| Upadacitinib + MTX vs. abatacept + MTX (1)† | 1.14 (0.57 to 2.27) | 0.702 | not applicable |
| Abatacept + MTX vs. adalimumab + MTX (1)‡ | 0.94 (0.50 to 1.77) | 0.853 | not applicable |
| DAS 28 < 2.6 vs. Boolean definition | | | |
| Sarilumab vs. adalimumab; monotherapy (1)* | 2.69 (0.38 to 18.97) | 0.320 | not applicable |
| Tocilizumab vs. adalimumab; monotherapy (1)* | 2.27 (0.54 to 10.00) | 0.261 | not applicable |
| Tocilizumab + MTX vs. adalimumab + MTX (1)† | 0.89 (0.11 to 7.14) | 0.918 | not applicable |
| Certolizumab pegol + MTX vs. adalimumab + MTX (1)‡ | 1.19 (0.74 to 1.92) | 0.473 | not applicable |
| Baricitinib + MTX vs. adalimumab + MTX (1)‡ | 0.83 (0.51 to 1.36) | 0.457 | not applicable |
| Filgotinib + MTX vs. adalimumab + MTX (1)‡ | 0.96 (0.60 to 1.51) | 0.846 | not applicable |
| Tofacitinib + MTX vs. adalimumab + MTX (2)§ | 0.77 (0.50 to 1.20) | 0.255 | 0.757 / 0 |
| Upadacitinib + MTX vs. adalimumab + MTX (1)‡ | 0.93 (0.56 to 1.54) | 0.776 | not applicable |
| Upadacitinib + MTX vs. abatacept + MTX (1)† | 1.38 (0.71 to 2.70) | 0.341 | not applicable |
| Abatacept + MTX vs. adalimumab + MTX (1)‡ | 1.06 (0.56 to 2.02) | 0.857 | not applicable |
| SDAI ≤ 3.3 vs. Boolean definition | | | |
| Sarilumab vs. adalimumab; monotherapy (1)* | 1.89 (0.19 to 18.62) | 0.585 | not applicable |
| Tocilizumab vs. adalimumab; monotherapy (1)* | 1.00 (0.23 to 4.40) | 1.000 | not applicable |
| Tocilizumab + MTX vs. adalimumab + MTX (1)† | 2.08 (0.11 to 50.00) | 0.632 | not applicable |
| Certolizumab pegol + MTX vs. adalimumab + MTX (1)‡ | 1.12 (0.68 to 1.89) | 0.651 | not applicable |
| Baricitinib + MTX vs. adalimumab + MTX (1)‡ | 1.09 (0.64 to 1.86) | 0.763 | not applicable |
| Filgotinib + MTX vs. adalimumab + MTX (1)‡ | 0.94 (0.58 to 1.52) | 0.791 | not applicable |
| Tofacitinib + MTX vs. adalimumab + MTX (2)§ | 0.94 (0.58 to 1.53) | 0.807 | 0.973 / 0 |
| Upadacitinib + MTX vs. adalimumab + MTX (1)‡ | 0.99 (0.57 to 1.73) | 0.984 | not applicable |
| Upadacitinib + MTX vs. abatacept + MTX (1)† | 1.16 (0.56 to 2.41) | 0.698 | not applicable |
| Abatacept + MTX vs. adalimumab + MTX (1)‡ | 1.13 (0.55 to 2.29) | 0.744 | not applicable |
| CDAI ≤ 2.8 vs. Boolean definition | | | |
| Sarilumab vs. adalimumab; monotherapy (1)* | 1.66 (0.17 to 16.53) | 0.665 | not applicable |
| Tocilizumab vs. adalimumab; monotherapy (1)* | 0.65 (0.16 to 2.78) | 0.560 | not applicable |
| Tocilizumab + MTX vs. adalimumab + MTX (1)† | 0.97 (0.07 to 14.29) | 0.982 | not applicable |
| Certolizumab pegol + MTX vs. adalimumab + MTX (1)‡ | 1.19 (0.72 to 1.96) | 0.499 | not applicable |
| Baricitinib + MTX vs. adalimumab + MTX (1)‡ | 1.04 (0.61 to 1.79) | 0.874 | not applicable |
| Filgotinib + MTX vs. adalimumab + MTX (1)‡ | 0.99 (0.61 to 1.62) | 0.978 | not applicable |
| Tofacitinib + MTX vs. adalimumab + MTX (2)§ | 1.05 (0.65 to 1.69) | 0.854 | 0.995 / 0 |
| Upadacitinib + MTX vs. adalimumab + MTX (1)‡ | 0.93 (0.53 to 1.61) | 0.794 | not applicable |
| Upadacitinib + MTX vs. abatacept + MTX (1)† | 1.01 (0.49 to 2.09) | 0.976 | not applicable |
| Abatacept + MTX vs. adalimumab + MTX (1)‡ | 1.19 (0.59 to 2.41) | 0.619 | not applicable |
| CDAI=clinical disease activity index; DAS 28=disease activity score 28; MTX=methotrexate; SDAI=simplified disease activity index. Effects shown in bold font indicate a statistically significant ratio of odds ratios. *Includes only studies on patients with MTX intolerance. †Includes only studies on patients after biologic failure. ‡Includes only studies on patients after MTX failure. §Includes only studies on patients after MTX or biologic failure. | | | |

## Forest plots

Forest plots are shown for comparisons including more than one study.

### Biologics vs. placebo

#### Abatacept vs. placebo

Supplement Figure 82: DAS 28 vs. CDAI, low disease activity, abatacept vs. placebo (combined with MTX), sensitivity analysis

Supplement Figure 83: DAS 28 vs. SDAI, low disease activity, abatacept vs. placebo (combined with MTX), sensitivity analysis

Supplement Figure 84: SDAI vs. CDAI, low disease activity, abatacept vs. placebo (combined with MTX), sensitivity analysis

Supplement Figure 85: DAS 28 vs. CDAI, remission, abatacept vs. placebo (combined with MTX), sensitivity analysis

Supplement Figure 86: DAS 28 vs. SDAI, remission, abatacept vs. placebo (combined with MTX), sensitivity analysis

Supplement Figure 87: SDAI vs. CDAI, remission, abatacept vs. placebo (combined with MTX), sensitivity analysis

Supplement Figure 88: DAS 28 vs. Boolean approach, remission, abatacept vs. placebo (combined with MTX), sensitivity analysis

Supplement Figure 89: SDAI vs. Boolean approach, remission, abatacept vs. placebo (combined with MTX), sensitivity analysis

Supplement Figure 90: CDAI vs. Boolean approach, remission, abatacept vs. placebo (combined with MTX), sensitivity analysis

#### Adalimumab vs. placebo

Supplement Figure 91: DAS 28 vs. CDAI, low disease activity, adalimumab vs. placebo (combined with MTX), sensitivity analysis

Supplement Figure 92: DAS 28 vs. SDAI, low disease activity, adalimumab vs. placebo (combined with MTX), sensitivity analysis

Supplement Figure 93: SDAI vs. CDAI, low disease activity, adalimumab vs. placebo (combined with MTX), sensitivity analysis

Supplement Figure 94: DAS 28 vs. CDAI, remission, adalimumab vs. placebo (combined with MTX), sensitivity analysis

Supplement Figure 95: DAS 28 vs. SDAI, remission, adalimumab vs. placebo (combined with MTX), sensitivity analysis

Supplement Figure 96: SDAI vs. CDAI, remission, adalimumab vs. placebo (combined with MTX), sensitivity analysis

Supplement Figure 97: DAS 28 vs. Boolean approach, remission, adalimumab vs. placebo (combined with MTX), sensitivity analysis

Supplement Figure 98: SDAI vs. Boolean approach, remission, adalimumab vs. placebo (combined with MTX), sensitivity analysis

Supplement Figure 99: CDAI vs. Boolean approach, remission, adalimumab vs. placebo (combined with MTX), sensitivity analysis

#### Anakinra vs. placebo

Supplement Figure 100: DAS 28 vs. CDAI, low disease activity, anakinra vs. placebo (combined with MTX), sensitivity analysis

Supplement Figure 101: DAS 28 vs. SDAI, low disease activity, anakinra vs. placebo (combined with MTX), sensitivity analysis

Supplement Figure 102: SDAI vs. CDAI, low disease activity, anakinra vs. placebo (combined with MTX), sensitivity analysis

Supplement Figure 103: DAS 28 vs. CDAI, remission, anakinra vs. placebo (combined with MTX), sensitivity analysis

Supplement Figure 104: DAS 28 vs. SDAI, remission, anakinra vs. placebo (combined with MTX), sensitivity analysis

Supplement Figure 105: SDAI vs. CDAI, remission, anakinra vs. placebo (combined with MTX), sensitivity analysis

Supplement Figure 106: DAS 28 vs. Boolean approach, remission, anakinra vs. placebo (combined with MTX), sensitivity analysis

Supplement Figure 107: SDAI vs. Boolean approach, remission, anakinra vs. placebo (combined with MTX), sensitivity analysis

Supplement Figure 108: CDAI vs. Boolean approach, remission, anakinra vs. placebo (combined with MTX), sensitivity analysis

#### Certolizumab pegol vs. placebo

Supplement Figure 109: DAS 28 vs. CDAI, low disease activity, certolizumab pegol vs. placebo (combined with MTX), sensitivity analysis

Supplement Figure 110: DAS 28 vs. SDAI, low disease activity, certolizumab pegol vs. placebo (combined with MTX), sensitivity analysis

Supplement Figure 111: SDAI vs. CDAI, low disease activity, certolizumab pegol vs. placebo (combined with MTX), sensitivity analysis

Supplement Figure 112: DAS 28 vs. CDAI, remission, certolizumab pegol vs. placebo (combined with MTX), sensitivity analysis

Supplement Figure 113: DAS 28 vs. SDAI, remission, certolizumab pegol vs. placebo (combined with MTX), sensitivity analysis

Supplement Figure 114: SDAI vs. CDAI, remission, certolizumab pegol vs. placebo (combined with MTX), sensitivity analysis

Supplement Figure 115: DAS 28 vs. Boolean approach, remission, certolizumab pegol vs. placebo (combined with MTX), sensitivity analysis

Supplement Figure 116: SDAI vs. Boolean approach, remission, certolizumab pegol vs. placebo (combined with MTX), sensitivity analysis

Supplement Figure 117: CDAI vs. Boolean approach, remission, certolizumab pegol vs. placebo (combined with MTX), sensitivity analysis

#### Etanercept vs. placebo

Supplement Figure 118: DAS 28 vs. CDAI, low disease activity, etanercept vs. placebo (combined with MTX), sensitivity analysis

Supplement Figure 119: DAS 28 vs. SDAI, low disease activity, etanercept vs. placebo (combined with MTX), sensitivity analysis

Supplement Figure 120: SDAI vs. CDAI, low disease activity, etanercept vs. placebo (combined with MTX), sensitivity analysis

Supplement Figure 121: DAS 28 vs. CDAI, remission, etanercept vs. placebo (combined with MTX), sensitivity analysis

Supplement Figure 122: DAS 28 vs. SDAI, remission, etanercept vs. placebo (combined with MTX), sensitivity analysis

Supplement Figure 123: SDAI vs. CDAI, remission, etanercept vs. placebo (combined with MTX), sensitivity analysis

Supplement Figure 124: DAS 28 vs. Boolean approach, remission, etanercept vs. placebo (combined with MTX), sensitivity analysis

Supplement Figure 125: SDAI vs. Boolean approach, remission, etanercept vs. placebo (combined with MTX), sensitivity analysis

Supplement Figure 126: CDAI vs. Boolean approach, remission, etanercept vs. placebo (combined with MTX), sensitivity analysis

#### Golimumab vs. placebo

Supplement Figure 127: DAS 28 vs. CDAI, low disease activity, golimumab vs. placebo (combined with MTX), sensitivity analysis

Supplement Figure 128: DAS 28 vs. SDAI, low disease activity, golimumab vs. placebo (combined with MTX), sensitivity analysis

Supplement Figure 129: SDAI vs. CDAI, low disease activity, golimumab vs. placebo (combined with MTX), sensitivity analysis

Supplement Figure 130: DAS 28 vs. CDAI, remission, golimumab vs. placebo (combined with MTX), sensitivity analysis

Supplement Figure 131: DAS 28 vs. SDAI, remission, golimumab vs. placebo (combined with MTX), sensitivity analysis

Supplement Figure 132: SDAI vs. CDAI, remission, golimumab vs. placebo (combined with MTX), sensitivity analysis

Supplement Figure 133: DAS 28 vs. Boolean approach, remission, golimumab vs. placebo (combined with MTX), sensitivity analysis

Supplement Figure 134: SDAI vs. Boolean approach, remission, golimumab vs. placebo (combined with MTX), sensitivity analysis

Supplement Figure 135: CDAI vs. Boolean approach remission, golimumab vs. placebo (combined with MTX), sensitivity analysis

#### Rituximab vs. placebo

Supplement Figure 136: DAS 28 vs. CDAI, low disease activity, rituximab vs. placebo (combined with MTX), sensitivity analysis

Supplement Figure 137: DAS 28 vs. SDAI, low disease activity, rituximab vs. placebo (combined with MTX), sensitivity analysis

Supplement Figure 138: SDAI vs. CDAI, low disease activity, rituximab vs. placebo (combined with MTX), sensitivity analysis

Supplement Figure 139: DAS 28 vs. CDAI, remission, rituximab vs. placebo (combined with MTX), sensitivity analysis

Supplement Figure 140: DAS 28 vs. SDAI, remission, rituximab vs. placebo (combined with MTX), sensitivity analysis

Supplement Figure 141: SDAI vs. CDAI, remission, rituximab vs. placebo (combined with MTX), sensitivity analysis

Supplement Figure 142: DAS 28 vs. Boolean approach, remission, rituximab vs. placebo (combined with MTX), sensitivity analysis

Supplement Figure 143: SDAI vs. Boolean approach, remission, rituximab vs. placebo (combined with MTX), sensitivity analysis

Supplement Figure 144: CDAI vs. Boolean approach, remission, rituximab vs. placebo (combined with MTX), sensitivity analysis

#### Tocilizumab vs. placebo

Supplement Figure 145: DAS 28 vs. CDAI, low disease activity, tocilizumab vs. placebo (combined with MTX), sensitivity analysis

Supplement Figure 146: DAS 28 vs. SDAI, low disease activity, tocilizumab vs. placebo (combined with MTX), sensitivity analysis

Supplement Figure 147: SDAI vs. CDAI, low disease activity, tocilizumab vs. placebo (combined with MTX), sensitivity analysis

Supplement Figure 148: DAS 28 vs. CDAI, remission, tocilizumab vs. placebo (combined with MTX), sensitivity analysis

Supplement Figure 149: DAS 28 vs. SDAI, remission, tocilizumab vs. placebo (combined with MTX), sensitivity analysis

Supplement Figure 150: SDAI vs. CDAI, remission, tocilizumab vs. placebo (combined with MTX), sensitivity analysis

Supplement Figure 151: DAS 28 vs. Boolean approach, remission, tocilizumab vs. placebo (combined with MTX), sensitivity analysis

Supplement Figure 152: SDAI vs. Boolean approach, remission, tocilizumab vs. placebo (combined with MTX), sensitivity analysis

Supplement Figure 153: CDAI vs. Boolean approach, remission, tocilizumab vs. placebo (combined with MTX), sensitivity analysis

### JAK inhibitor (tofacitinib) vs. TNFα inhibitor (adalimumab)

Supplement Figure 154: DAS 28 vs. CDAI, low disease activity, tofacitinib vs. adalimumab (combined with MTX), sensitivity analysis

Supplement Figure 155: DAS 28 vs. SDAI, low disease activity, tofacitinib vs. adalimumab (combined with MTX), sensitivity analysis

Supplement Figure 156: SDAI vs. CDAI, low disease activity, tofacitinib vs. adalimumab (combined with MTX), sensitivity analysis

Supplement Figure 157: DAS 28 vs. CDAI, remission, tofacitinib vs. adalimumab (combined with MTX), sensitivity analysis

Supplement Figure 158: DAS 28 vs. SDAI, remission, tofacitinib vs. adalimumab (combined with MTX), sensitivity analysis

Supplement Figure 159: SDAI vs. CDAI, remission, tofacitinib vs. adalimumab (combined with MTX), sensitivity analysis

Supplement Figure 160: DAS 28 vs. Boolean approach, remission, tofacitinib vs. adalimumab (combined with MTX), sensitivity analysis

Supplement Figure 161: SDAI vs. Boolean approach, remission, tofacitinib vs. adalimumab (combined with MTX), sensitivity analysis

Supplement Figure 162: CDAI vs. Boolean approach, remission, tofacitinib vs. adalimumab (combined with MTX), sensitivity analysis

# Data for low disease activity and remission measured by DAS 28, SDAI, CDAI and Boolean definition (remission only) from single studies

Supplement Table 11: Results on remission available for the DAS 28 < 2.6. SDAI ≤ 3.3. CDAI ≤ 2.8 or Boolean definition from single studies investigating MTX-naïve patients

| Study  Analysis time point  Treatment arm | N* | CDAI ≤ 2.8  n / N (%) | SDAI ≤ 3.3  n / N (%) | Boolean definition†  n / N (%) | DAS 28 [CRP] < 2.6  n / N (%) |
| --- | --- | --- | --- | --- | --- |
| AGREE |  |  |  |  |  |
| 24 weeks |  |  |  |  |  |
| Abatacept + MTX | 256 | 45 (17.6) | 44 (17.2) | 29 (11.3) | 72 (28.1) |
| Placebo + MTX | 252 | 22 (8.7) | 22 (8.7) | 9 (3.6) | 39 (15.4) |
| 52 weeks |  |  |  |  |  |
| Abatacept + MTX | 256 | 76 (29.7) | 70 (27.3) | 43 (16.8) | 106 (41.4) |
| Placebo + MTX | 252 | 36 (14.2) | 27 (10.7) | 10 (4.0) | 59 (23.3) |
| AVERT |  |  |  |  |  |
| 24 weeks |  |  |  |  |  |
| Abatacept + MTX | 119 | 37 (31.1) | 37 (31.1) | 22 (18.5) | 54 (45.4) |
| Placebo + MTX | 116 | 12 (10.3) | 13 (11.2) | 6 (5.2) | 31 (26.7) |
| 52 weeks |  |  |  |  |  |
| Abatacept + MTX | 119 | 50 (42.0) | 50 (42.0) | 44 (37.0) | 73 (61.3) |
| Placebo + MTX | 116 | 32 (27.6) | 29 (25.0) | 26 (22.4) | 53 (45.7) |
| HIT-HARD |  |  |  |  |  |
| 24 weeks |  |  |  |  |  |
| Adalimumab + MTX | 87 | 29‡ (33.8§) | 27‡ (31.5§) | 26‡ (30§) | 42‡ (48.5§) |
| Placebo + MTX | 85 | 13‡ (15.3§) | 12‡ (14.1§) | 10‡ (11.8§) | 24‡ (28.5§) |
| HOPEFUL-1 |  |  |  |  |  |
| 26 weeks |  |  |  |  |  |
| Adalimumab + MTX | 171 | 34 (19.9) | 38 (22.2) | 33 (19.3) | 87 (50.9) |
| Placebo + MTX | 163 | 18 (11.0) | 20 (12.3) | 14 (8.6) | 43 (26.4) |
| OPTIMA (phase 1) |  |  |  |  |  |
| 26 weeks |  |  |  |  |  |
| Adalimumab + MTX | 515 | 103 (20.0) | 102 (19.8) | 79 (15.3) | 174 (33.8) |
| Placebo + MTX | 517 | 56 (10.8) | 53 (10.3) | 36 (7.0) | 86 (16.6) |
| PREMIER |  |  |  |  |  |
| 26 weeks |  |  |  |  |  |
| Adalimumab + MTX | 268 | 59 (22.0) | 61 (22.8) | 50 (18.7) | 97 (36.2) |
| Placebo + MTX | 257 | 35 (13.6) | 31 (12.1) | 21 (8.2) | 48 (18.7) |
| 52 weeks |  |  |  |  |  |
| Adalimumab + MTX | 268 | 76 (28.4) | 74 (27.6) | 65 (24.3) | 115 (42.9) |
| Placebo + MTX | 257 | 43 (16.7) | 36 (14.0) | 25 (9.7) | 53 (20.6) |
| 104 weeks |  |  |  |  |  |
| Adalimumab + MTX | 268 | 101 (37.7) | 99 (36.9) | 82 (30.6) | 132 (49.3) |
| Placebo + MTX | 257 | 45 (17.5) | 41 (16.0) | 35 (13.6) | 64 (24.9) |
| PROWD |  |  |  |  |  |
| 24 weeks |  |  |  |  |  |
| Adalimumab + MTX | 75 | 23 (30.7) | 23 (30.7) | 20 (26.7) | 33 (44.0)\|\| |
| Placebo + MTX | 73 | 13 (17.8) | 14 (19.2) | 9 (12.3) | 16 (21.9)\|\| |
| 56 weeks |  |  |  |  |  |
| Adalimumab + MTX | 75 | 24 (32.0) | 23 (30.7) | 21 (28.0) | 32 (42.7)\|\| |
| Placebo + MTX | 73 | 21 (28.8) | 19 (26.0) | 18 (24.7) | 26 (35.6)\|\| |
| C-EARLY (phase I) |  |  |  |  |  |
| 24 weeks |  |  |  |  |  |
| Certolizumab pegol + MTX | 655 | 161 (24.6) | 164 (25.0) | 131 (20.0) | 171 (26.1)\|\| |
| Placebo + MTX | 213 | 27 (12.7) | 28 (13.1) | 26 (12.2) | 28 (13.1)\|\| |
| 52 weeks |  |  |  |  |  |
| Certolizumab pegol + MTX | 655 | 255 (38.9) | 255 (38.9) | 212 (32.4) | 279 (42.6)\|\| |
| Placebo + MTX | 213 | 56 (26.3) | 53 (24.9) | 44 (20.7) | 57 (26.8)\|\| |
| C-OPERA |  |  |  |  |  |
| 24 weeks |  |  |  |  |  |
| Certolizumab pegol + MTX | 159 | 69 (43.4) | 77 (48.4) | 58 (36.5) | 84 (52.8)\|\| |
| Placebo + MTX | 157 | 36 (22.9) | 46 (29.3) | 35 (22.3) | 49 (31.2)\|\| |
| 52 weeks |  |  |  |  |  |
| Certolizumab pegol + MTX | 159 | 85 (53.5) | 92 (57.9) | 72 (45.3) | 91 (57.2)\|\| |
| Placebo + MTX | 157 | 52 (33.1) | 53 (33.8) | 44 (28.0) | 59 (37.6)\|\| |
| COMET |  |  |  |  |  |
| 24 weeks |  |  |  |  |  |
| Etanercept + MTX | 265 | 47 (17.7) | 45 / 261 (17.2) | 43 (16.2) | 111 (41.9)\|\| |
| Placebo + MTX | 263 | 19 (7.2) | 16 / 251 (6.4) | 22 (8.4) | 62 (23.6)\|\| |
| 52 weeks |  |  |  |  |  |
| Etanercept + MTX | 265 | 66 (24.9) | 59 (22.6) | 61 (23.0) | 132 (49.8)\|\| |
| Placebo + MTX | 263 | 27 (10.3) | 26 (10.4) | 26 (10.0) | 73 (27.8)\|\| |
| TEMPO |  |  |  |  |  |
| 24 weeks |  |  |  |  |  |
| Etanercept + MTX | 28 | 4 (14.3) | 4 (14.3) | 4 (14.3) | 7 (25.0)\|\| |
| Placebo + MTX | 32 | 2 (6.3) | 2 (6.3) | 1 (3.1) | 4 (12.5)\|\| |
| 52 weeks |  |  |  |  |  |
| Etanercept + MTX | 28 | 3 (10.7) | 3 (10.7) | 5 (17.9) | 11 (39.3)\|\| |
| Placebo + MTX | 32 | 3 (9.4) | 3 (9.4) | 3 (9.4) | 6 (18.8)\|\| |
| 164 weeks |  |  |  |  |  |
| Etanercept + MTX | 28 | 6 (21.4) | 6 (21.4) | 8 (28.6) | 12 (42.9)\|\| |
| Placebo + MTX | 32 | 3 (9.4) | 3 (9.4) | 3 (9.4) | 6 (18.8)\|\| |
| GO-BEFORE |  |  |  |  |  |
| 24 weeks |  |  |  |  |  |
| Golimumab + MTX | 81 | 16 (20.3) | 16 (20.3) | 15 (19.0) | 26 (32.9) |
| Placebo + MTX | 73 | 9 (12.7) | 9 (12.7) | 8 (11.3) | 12 (16.9) |
| 52 weeks |  |  |  |  |  |
| Golimumab + MTX | 81 | 24 (32.0) | 22 (29.3) | 16 (21.3) | 34 (45.3) |
| Placebo + MTX | 73 | 14 (21.5) | 13 (20.0) | 12 (18.5) | 17 (26.2) |
| FUNCTION |  |  |  |  |  |
| 24 weeks |  |  |  |  |  |
| Tocilizumab + MTX | 290 | 71 (24.5) | 79 (27.2) | 61 (21.0) | 130 (44.8)\|\| |
| Placebo + MTX | 287 | 38 (13.2) | 43 (15.0) | 38 (13.2) | 43 (15.0)\|\| |
| 52 weeks |  |  |  |  |  |
| Tocilizumab + MTX | 290 | 93 (32.1) | 94 (32.4) | 80 (27.6) | 143 (49.3)\|\| |
| Placebo + MTX | 287 | 56 (19.5) | 53 (18.5) | 51 (17.8) | 58 (20.2)\|\| |
| 104 weeks |  |  |  |  |  |
| Tocilizumab + MTX | 290 | 110 (37.9) | 117 (40.3) | 96 (33.1) | 138 (47.6)\|\| |
| Placebo + MTX | 287 | 58 (20.2) | 54 (18.8) | 54 (18.8) | 46 (31.7)\|\| |
| U-ACT-EARLY |  |  |  |  |  |
| 24 weeks |  |  |  |  |  |
| Tocilizumab + MTX | 79 | 35 (44.3) | 25 (31.6) | 28 / 98 (28.6) | 88 / 100 (88.0) |
| Placebo + MTX | 84 | 12 (14.3) | 7 / 83 (8.4) | 3 / 98 (3.1) | 41 / 98 (41.8) |
| 52 weeks |  |  |  |  |  |
| Tocilizumab + MTX | 81 | 42 (51.9) | 23 (28.4) | 17 / 86 (19.8) | 74 / 86 (86.0) |
| Placebo + MTX | 77 | 30 (39.0) | 15 / 76 (19.7) | 15 / 87 (17.2) | 68 / 88 (77.3) |
| 104 weeks |  |  |  |  |  |
| Tocilizumab + MTX | 81 | 33 (40.7)¶ | 17 (21.0)¶ | 16 / 95 (16.8)¶ | 64 / 106 (60.4)¶ |
| Placebo + MTX | 86 | 29 (33.7)¶ | 14 (16.3)¶ | 10 / 102 (9.8)¶ | 62 / 108 (57.4)¶ |
| CDAI=clinical disease activity index; CRP=C-reactive protein; DAS 28=disease activity score 28; ESR=erythrocyte sedimentation rate; MTX=methotrexate; N=number of patients included in the analysis; n=number of patients with event; SDAI=simplified disease activity index. *Number of patients included in the analysis. If the number differed between the composite measures, the numbers are mentioned separately as n / N. † ≤ 1 swollen joint, ≤ 1 tender joint, C-reactive protein ≤ 1 mg/dl and global assessment of disease activity by the patient ≤ 1 on a scale from 0 to 10. ‡ Calculated from percentages. § Results for 10-fold multiple imputation. \|\| DAS 28 [ESR]. ¶ Calculated with the assumption that patients with missing values did not achieve remission. | | | | | |

Supplement Table 12: Results on low disease activity available for the DAS 28 < 3.2. SDAI ≤ 11 or CDAI ≤ 10 from single studies investigating MTX-naïve patients

| Study  Analysis time point  Treatment arm | | N* | | CDAI ≤ 10  n / N (%) | | SDAI ≤ 11  n / N (%) | | | DAS 28 [CRP] < 3.2  n / N (%) | |
| --- | --- | --- | --- | --- | --- | --- | --- | --- | --- | --- |
| AGREE | |  | |  | |  | | |  | |
| 24 weeks | |  | |  | |  | | |  | |
| Abatacept + MTX | | 256 | | 121 (47.3) | | 121 (47.3) | | | 109 (42.6) | |
| Placebo + MTX | | 252 | | 82 (32.4) | | 81 (32.0) | | | 71 (28.1) | |
| 52 weeks | |  | |  | |  | | |  | |
| Abatacept + MTX | | 256 | | 148 (57.8) | | 144 (56.3) | | | 139 (54.3) | |
| Placebo + MTX | | 252 | | 96 (37.9) | | 93 (36.8) | | | 93 (36.8) | |
| AVERT | |  | |  | |  | | |  | |
| 24 weeks | |  | |  | |  | | |  | |
| Abatacept + MTX | | 119 | | 72 (60.5) | | 73 (61.3) | | | 76 (63.9) | |
| Placebo + MTX | | 116 | | 48 (41.4) | | 50 (43.1) | | | 47 (40.5) | |
| 52 weeks | |  | |  | |  | | |  | |
| Abatacept + MTX | | 119 | | 83 (69.7) | | 84 (70.6) | | | 84 (70.6) | |
| Placebo + MTX | | 116 | | 74 (63.8) | | 74 (63.8) | | | 72 (62.1) | |
| HIT-HARD | |  | |  | |  | | |  | |
| 24 weeks | |  | |  | |  | | |  | |
| Adalimumab + MTX | | 87 | | 57† (66‡) | | 57† (66‡) | | | 58† (66.9‡) | |
| Placebo + MTX | | 85 | | 40† (46.5‡) | | 38† (44.9‡) | | | 40† (46.9‡) | |
| HOPEFUL-1 | |  | |  | |  | | |  | |
| 26 weeks | |  | |  | |  | | |  | |
| Adalimumab + MTX | | 171 | | 102 (59.6) | | 103 (60.2) | | | 111 (64.9) | |
| Placebo + MTX | | 163 | | 56 (34.4) | | 57 (35.0) | | | 57 (35.0) | |
| OPTIMA (phase 1) | |  | |  | |  | | |  | |
| 26 weeks | |  | |  | |  | | |  | |
| Adalimumab + MTX | | 515 | | 244 (47.4) | | 248 (48.2) | | | 240 (46.6) | |
| Placebo + MTX | | 517 | | 157 (30.4) | | 153 (29.6) | | | 132 (25.5) | |
| PREMIER | |  | |  | |  | |  | | |
| 26 weeks | |  | |  | |  | |  | | |
| Adalimumab + MTX | | 268 | | 153 (57.1) | | 149 (55.6) | | 144 (53.7)§ | | |
| Placebo + MTX | | 257 | | 86 (33.5) | | 83 (32.3) | | 82 (31.9)§ | | |
| 52 weeks | |  | |  | |  | |  | | |
| Adalimumab + MTX | | 268 | | 159 (59.3) | | 160 (59.7) | | 154 (57.5)§ | | |
| Placebo + MTX | | 257 | | 91 (35.4) | | 88 (34.2) | | 83 (32.3)§ | | |
| 104 weeks | |  | |  | |  | |  | | |
| Adalimumab + MTX | | 268 | | 158 (59.0) | | 159 (59.3) | | 158 (59.0)§ | | |
| Placebo + MTX | | 257 | | 95 (37.0) | | 89 (34.6) | | 92 (35.8)§ | | |
| PROWD | |  | |  | |  | |  | | |
| 24 weeks | |  | |  | |  | |  | | |
| Adalimumab + MTX | | 75 | | 42 (56.0) | | 42 (56.0) | | 42 (56.0)§ | | |
| Placebo + MTX | | 73 | | 29 (39.7) | | 29 (39.7) | | 29 (39.7)§ | | |
| 56 weeks | |  | |  | |  | |  | | |
| Adalimumab + MTX | | 75 | | 41 (54.7) | | 39 (52.0) | | 39 (52.0)§ | | |
| Placebo + MTX | | 73 | | 32 (43.8) | | 34 (46.6) | | 33 (45.2)§ | | |
| C-EARLY (phase I) | |  | |  | |  | |  | | |
| 24 weeks | |  | |  | |  | |  | | |
| Certolizumab pegol + MTX | | 655 | | 358 (54.7) | | 357 (54.5) | | 260 (39.7)§ | | |
| Placebo + MTX | | 213 | | 95 (44.6) | | 97 (45.5) | | 65 (30.5)§ | | |
| 52 weeks | |  | |  | |  | |  | | |
| Certolizumab pegol + MTX | | 655 | | 403 (61.5) | | 404 (61.7) | | 358 (54.7)§ | | |
| Placebo + MTX | | 213 | | 104 (48.8) | | 103 (48.4) | | 84 (39.4)§ | | |
| C-OPERA | |  | |  | |  | |  | | |
| 24 weeks | |  | |  | |  | |  | | |
| Certolizumab pegol + MTX | | 159 | | 126 (79.2) | | 129 (81.1) | | 117 (73.6)§ | | |
| Placebo + MTX | | 157 | | 92 (58.6) | | 95 (60.5) | | 75 (47.8)§ | | |
| 52 weeks | |  | |  | |  | |  | | |
| Certolizumab pegol + MTX | | 159 | | 124 (78.0) | | 128 (80.5) | | 112 (70.4)§ | | |
| Placebo + MTX | | 157 | | 85 (54.1) | | 85 (54.1) | | 68 (43.3)§ | | |
| COMET | |  | |  | |  | |  | | |
| 24 weeks | |  | |  | |  | |  | | |
| Etanercept + MTX | | 265 | | 171 (64.5) | | 167 / 261 (64.0) | | 157 (59.2)§ | | |
| Placebo + MTX | | 263 | | 105 (39.9) | | 100 / 251 (39.8) | | 98 (37.3)§ | | |
| 52 weeks | |  | |  | |  | |  | | |
| Etanercept + MTX | | 265 | | 186 (70.2) | | 187 / 261 (71.6) | | 170 (64.2)§ | | |
| Placebo + MTX | | 263 | | 128 (48.7) | | 120 / 251 (47.8) | | 109 (41.4)§ | | |
| TEMPO | |  | |  | |  | |  | | |
| 24 weeks | |  | |  | |  | |  | | |
| Etanercept + MTX | | 28 | | 13 (46.4) | | 13 (46.4) | | 15 (53.6)§ | | |
| Placebo + MTX | | 32 | | 8 (25.0) | | 8 (25.0) | | 5 (15.6)§ | | |
| 52 weeks | |  | |  | |  | |  | | |
| Etanercept + MTX | | 28 | | 18 (64.3) | | 18 (64.3) | | 19 (67.9)§ | | |
| Placebo + MTX | | 32 | | 10 (31.3) | | 10 (31.3) | | 10 (31.3)§ | | |
| 164 weeks | |  | |  | |  | |  | | |
| Etanercept + MTX | | 28 | | 17 (60.7) | | 17 (60.7) | | 17 (60.7)§ | | |
| Placebo + MTX | | 32 | | 12 (37.5) | | 12 (37.5) | | 9 (28.1)§ | | |
| GO-BEFORE | |  | |  | |  | |  | | |
| 24 weeks | |  | |  | |  | |  | | |
| Golimumab + MTX | | 81 | | 41 (51.9) | | 43 (54.4) | | 38 (48.1) | | |
| Placebo + MTX | | 73 | | 23 (32.4) | | 23 (32.4) | | 23 (32.4) | | |
| 52 weeks | |  | |  | |  | |  | | |
| Golimumab + MTX | | 81 | | 41 (54.7) | | 41 (54.7) | | 40 (53.3) | | |
| Placebo + MTX | | 73 | | 35 (53.8) | | 35 (53.8) | | 33 (50.8) | | |
| FUNCTION | |  | |  | |  | |  | | |
| 24 weeks | |  | |  | |  | |  | | |
| Tocilizumab + MTX | | 290 | | 159 (54.8) | | 173 (59.7) | | 167 (57.6) | | |
| Placebo + MTX | | 287 | | 129 (45.0) | | 134 (46.7) | | 77 (26.8) | | |
| 52 weeks | |  | |  | |  | |  | | |
| Tocilizumab + MTX | | 290 | | 178 (61.4) | | 183 (62.7) | | 168 (57.9) | | |
| Placebo + MTX | | 287 | | 132 (46.0) | | 137 (47.7) | | 86 (30.0) | | |
| 104 weeks | |  | |  | |  | |  | | |
| Tocilizumab + MTX | | 290 | | 185 (63.8) | | 190 (65.5) | | 161 (55.5) | | |
| Placebo + MTX | | 287 | | 121 (42.2) | | 127 (44.3) | | 61 (42.1) | | |
| U-ACT-EARLY |  | |  | |  | |  | | |  |
| 24 weeks |  | |  | |  | |  | | |  |
| Tocilizumab + MTX | 100 | | 69 / 79 (87.3) | | 63 / 79 (79.7) | | 95 (95.0) | | |  |
| Placebo + MTX | 98 | | 52 / 84 (61.9) | | 32 / 83 (38.6) | | 54 (55.1) | | |  |
| 52 weeks |  | |  | |  | |  | | |  |
| Tocilizumab + MTX | 86 | | 66 / 81 (81.5) | | 59 / 81 (72.8) | | 78 (90.7) | | |  |
| Placebo + MTX | 88 | | 63 / 77 (81.8) | | 58 / 76 (76.3) | | 76 (86.4) | | |  |
| 104 weeks |  | |  | |  | |  | | |  |
| Tocilizumab + MTX | 106 | | 55/ 81 (67.9)\|\| | | 52 / 81 (64.2)\|\| | | 69 (65.1)\|\| | | |  |
| Placebo + MTX | 108 | | 59 / 86 (68.6)\|\| | | 50 / 86 (58.1)\|\| | | 70 (64.8)\|\| | | |  |
| CDAI=clinical disease activity index; CRP=C-reactive protein; DAS 28=disease activity score 28; ESR=erythrocyte sedimentation rate; MTX=methotrexate; N=number of patients included in the analysis; n=number of patients with event; SDAI=simplified disease activity index. *Number of patients included in the analysis. If the number differed between the composite measures, the numbers are mentioned separately as n / N. †Calculated from percentages. ‡Results for 10-fold multiple imputation. § DAS 28 [ESR]. \|\| Calculated with the assumption that patients with missing values did not achieve low disease activity. | | | | | | | | | |  |

Supplement Table 13: Results on remission available for the DAS 28 < 2.6. SDAI ≤ 3.3. CDAI ≤ 2.8 or Boolean definition from single studies investigating patients after MTX failure

| Study  Analysis time point  Treatment arm | N* | CDAI ≤ 2.8  n / N (%) | SDAI ≤ 3.3  n / N (%) | Boolean definition†  n / N (%) | DAS 28 [CRP] < 2.6  n / N (%) |
| --- | --- | --- | --- | --- | --- |
| AIM |  |  |  |  |  |
| 24 weeks |  |  |  |  |  |
| Abatacept + MTX | 420 | 30 (7.1) | 19 / 418 (4.5) | 12 / 418 (2.9) | 62 / 418 (14.8) |
| Placebo + MTX | 211 | 4 (1.9) | 2 / 211 (0.9) | 3 / 211 (1.4) | 6 / 211 (2.8) |
| 52 weeks |  |  |  |  |  |
| Abatacept + MTX | 424 | 50 (11.8) | 43 (10.1) | 25 (5.9) | 101 (23.8) |
| Placebo + MTX | 212 | 5 (2.4) | 3 (1.4) | 3 (1.4) | 4 (1.9) |
| ATTEST |  |  |  |  |  |
| 24 weeks |  |  |  |  |  |
| Abatacept + MTX | 154 | 14 (9.1) | 13 (8.4) | 10 / 155 (6.5) | 31 / 155 (20.0) |
| Placebo + MTX | 109 | 4 (3.7) | 2 (1.8) | 1 (0.9) | 6 / 109 (5.5) |
| IM101071 |  |  |  |  |  |
| 24 weeks |  |  |  |  |  |
| Abatacept + MTX | 47 | 5 (10.6) | 5 (10.6) | 7 (14.9) | 13 (27.7) |
| Placebo + MTX | 41 | 0 (0) | 0 (0) | 0 (0) | 1 (2.4) |
| IM101100 |  |  |  |  |  |
| 24 weeks |  |  |  |  |  |
| Abatacept + MTX | 115 | 10 (8.7) | 9 (7.8) | 6 (5.2) | 15 (13.0) |
| Placebo + MTX | 119 | 0 (0) | 0 (0) | 0 (0) | 2 (1.7) |
| 52 weeks |  |  |  |  |  |
| Abatacept + MTX | 115 | 9 (7.8) | 6 (5.2) | 4 (3.5) | 19 (16.5) |
| Placebo + MTX | 119 | 1 (0.8) | 1 (0.8) | 0 (0) | 2 (1.7) |
| IM101124 |  |  |  |  |  |
| 24 weeks |  |  |  |  |  |
| Abatacept + MTX | 55 | 1 (1.8) | 1 (1.8) | 1 (1.8) | 13 (23.6) |
| Placebo + MTX | 57 | 3 (5.3) | 3 (5.3) | 1 (1.8) | 5 (8.8) |
| ARMADA |  |  |  |  |  |
| 24 weeks |  |  |  |  |  |
| Adalimumab + MTX | 63 | 8 (12.7) | 7 (11.1) | 8 (12.7) | 23 (36.5) |
| Placebo + MTX | 60 | 1 (1.7) | 1 (1.7) | 2 (3.3) | 2 (3.3) |
| August II |  |  |  |  |  |
| 26 weeks |  |  |  |  |  |
| Adalimumab + MTX | 79 | 9 (11.4) | 10 (12.7) | 7 (8.9) | 18 (22.8) |
| Placebo + MTX | 76 | 3 (3.9) | 3 (3.9) | 3 (3.9) | 8 (10.5) |
| DE019 |  |  |  |  |  |
| 24 weeks |  |  |  |  |  |
| Adalimumab + MTX | 207 | 17 (8.2) | 17 (8.2) | 14 (6.8) | 43 (20.8) |
| Placebo + MTX | 200 | 4 (2.0) | 4 (2.0) | 2 (1.0) | 9 (4.5) |
| 52 weeks |  |  |  |  |  |
| Adalimumab + MTX | 207 | 18 (8.7) | 19 (9.2) | 16 (7.7) | 47 (22.7) |
| Placebo + MTX | 200 | 6 (3.0) | 5 (2.5) | 4 (2.0) | 11 (5.5) |
| IM133001 |  |  |  |  |  |
| 24 weeks |  |  |  |  |  |
| Adalimumab + MTX | 59 | 5‡ (8.5) | 5‡ (8.5) | 6‡ (10.2) | 14‡ (23.7) |
| Placebo + MTX | 61 | 1‡ (1.6) | 3‡ (4.9) | 1‡ (1.6) | 7‡ (11.5) |
| M02-556 |  |  |  |  |  |
| 24 weeks |  |  |  |  |  |
| Adalimumab + MTX | 65 | 4 (6.2) | 7 (10.8) | 4 (6.2) | 20 (30.8) |
| Placebo + MTX | 63 | 1 (1.6) | 1 (1.6) | 1 (1.6) | 4 (6.3) |
| ORAL STANDARD |  |  |  |  |  |
| 26 weeks (phase I) |  |  |  |  |  |
| Adalimumab + MTX | 178 | 10 (5.6) | 12 (6.7) | 8 (4.5) | 33 (18.5) |
| Placebo + MTX | 92 | 2 (2.2) | 2 / 90 (2.2) | 2 (2.2) | 6 / 90 (6.7) |
| RA-BEAM |  |  |  |  |  |
| 24 weeks |  |  |  |  |  |
| Adalimumab + MTX | 330 | 39 (11.8) | 45 (13.6) | 33 (10.0) | 63 (19.1) |
| Placebo + MTX | 488 | 19 (3.9) | 15 (3.1) | 13 (2.7) | 21 (4.3) |
| STAR |  |  |  |  |  |
| 24 weeks |  |  |  |  |  |
| Adalimumab + MTX | 178 | 19 (10.7) | 18 (10.1) | 16 (9.0) | 35 (19.7) |
| Placebo + MTX | 199 | 2 (1.0) | 2 (1.0) | 2 (1.0) | 14 (7.0) |
| 990145 (part A) |  |  |  |  |  |
| 24 weeks |  |  |  |  |  |
| Anakinra + MTX | 449 | 4 (0.9) | 4 (0.9) | 5 (1.1) | 38 (8.5) |
| Placebo + MTX | 450 | 5 (1.1) | 4 (0.9) | 1 (0.2) | 14 (3.1) |
| 52 weeks |  |  |  |  |  |
| Anakinra + MTX | 449 | 9 (2.0) | 8 (1.8) | 8 (1.8) | 29 (6.5) |
| Placebo + MTX | 450 | 4 (0.9) | 2 (0.4) | 3 (0.7) | 11 (2.4) |
| 20000198 |  |  |  |  |  |
| 24 weeks |  |  |  |  |  |
| Anakinra + MTX | 68 | 1 (1.5) | 1 (1.5) | 1 (1.5) | 5 (7.4) |
| Placebo + MTX | 68 | 1 (1.5) | 1 (1.5) | 0 (0) | 2 (2.9) |
| CERTAIN |  |  |  |  |  |
| 24 weeks |  |  |  |  |  |
| Certolizumab pegol + DMARD | 96 | 18 (18.8)§ | 14 (14.6) | 10 (10.4) | 19 (19.8)\|\| |
| Placebo + DMARD | 98 | 6 (6.1)§ | 4 (4.1) | 5 (5.1) | 3 (3.1)\|\| |
| RAPID 1 (phase I) |  |  |  |  |  |
| 24 weeks |  |  |  |  |  |
| Certolizumab pegol + MTX | 392 | 41 (10.5) | 38 (9.7) | 31 (7.9) | 45 / 391 (11.5)\|\| |
| Placebo + MTX | 196 | 4 (2.0) | 4 (2.0) | 3 (1.5) | 3 (1.5)\|\| |
| 52 weeks |  |  |  |  |  |
| Certolizumab pegol + MTX | 392 | 58 (14.8) | 52 (13.3) | 45 (11.5) | 62 / 391 (15.9)\|\| |
| Placebo + MTX | 196 | 3 (1.5) | 3 (1.5) | 1 (0.5) | 3 (1.5)\|\| |
| RAPID 2 |  |  |  |  |  |
| 24 weeks |  |  |  |  |  |
| Certolizumab pegol + MTX | 245 | 14 (5.7) | 13 (5.3) | 7 (2.9) | 23 (9.4)\|\| |
| Placebo + MTX | 126 | 0 (0) | 0 (0) | 0 / 127 (0) | 1 / 125 (0.8)\|\| |
| RA0025 |  |  |  |  |  |
| 24 weeks |  |  |  |  |  |
| Certolizumab pegol + MTX | 70 | 7 (10.0) | 8 (11.4) | 4 (5.7) | 3 (4.3)\|\| |
| Placebo + MTX | 33 | 0 (0) | 0 (0) | 0 (0) | 0 (0)\|\| |
| TEMPO |  |  |  |  |  |
| 24 weeks |  |  |  |  |  |
| Etanercept + MTX | 103 | 12 (11.7) | 11 (10.7) | 9 (8.7) | 28 (27.2) |
| Placebo + MTX | 97 | 2 (2.1) | 2 (2.1) | 4 (4.1) | 13 (13.4) |
| 52 weeks |  |  |  |  |  |
| Etanercept + MTX | 103 | 16 (15.5) | 16 (15.5) | 18 (17.5) | 40 (38.8) |
| Placebo + MTX | 97 | 3 (3.1) | 3 (3.1) | 6 (6.2) | 19 (19.6) |
| 164 weeks |  |  |  |  |  |
| Etanercept + MTX | 103 | 26 (25.2) | 26 (25.2) | 25 (24.3) | 51 (49.5) |
| Placebo + MTX | 97 | 4 (4.1) | 3 (3.1) | 11 (11.3) | 21 (21.6) |
| 16.0014 |  |  |  |  |  |
| 24 weeks |  |  |  |  |  |
| Etanercept + MTX | 59 | 2 (3.4) | 5 (8.5) | 4 (6.8) | 22 (37.3) |
| Placebo + MTX | 30 | 0 (0) | 0 (0) | 0 (0) | 4 (13.3) |
| C0524T28 |  |  |  |  |  |
| 24 weeks |  |  |  |  |  |
| Golimumab + MTX | 132 | 6 (4.5) | 7 (5.3) | 2 (1.5) | 25 (18.9) |
| Placebo + MTX | 132 | 1 (0.8) | 1 (0.8) | 1 (0.8) | 10 (7.6) |
| GO-FORTH |  |  |  |  |  |
| 24 weeks |  |  |  |  |  |
| Golimumab + MTX | 81 | 14 (17.3) | 19 (23.5) | 15 (18.5) | 44 (54.3) |
| Placebo + MTX | 84 | 4 (4.8) | 4 (4.8) | 0 (0.0) | 15 (17.9) |
| GO-FORWARD |  |  |  |  |  |
| 24 weeks |  |  |  |  |  |
| Golimumab + MTX | 88 | 13 (14.8) | 12 (13.6) | 11 (12.5) | 24 (27.3) |
| Placebo + MTX | 127 | 3 (2.4) | 3 (2.4) | 2 (1.6) | 9 (7.1) |
| ATTRACT |  |  |  |  |  |
| 30 weeks |  |  |  |  |  |
| Infliximab + MTX | 84 | 5 (6.0) | 5 / 83 (6.0) | 3 (3.6) | 10 (11.9) |
| Placebo + MTX | 87 | 1 (1.1) | 0 / 86 (0.0) | 0 (0.0) | 3 (3.4) |
| 54 weeks |  |  |  |  |  |
| Infliximab + MTX | 82 | 3 (3.7) | 3 (3.7) | 1 / 84 (1.2) | 9 / 84 (10.7) |
| Placebo + MTX | 88 | 1 (1.1) | 1 (1.1) | 1 (1.1) | 3 (3.4) |
| LITHE |  |  |  |  |  |
| 24 weeks |  |  |  |  |  |
| Tocilizumab + MTX | 308¶ | 15 (4.9**) | 21 / 306¶ (6.9**) | 14 /316¶ (4.4**) | 90 / 321¶ (28.0**)\|\| |
| Placebo + MTX | 313¶ | 6 (1.9**) | 5 / 310¶ (1.6**) | 2 / 316¶ (0.6**) | 7 / 324¶ (2.2**)\|\| |
| 52 weeks |  |  |  |  |  |
| Tocilizumab + MTX | 301¶ | 36 (12.0**) | 40 / 297¶ (13.5**) | 24 / 307¶ (7.8**) | 118 (39.2**)\|\| |
| Placebo + MTX | 312¶ | 7 (2.2**) | 6 / 311¶ (1.9**) | 4 / 316¶ (1.3**) | 11 / 314¶ (3.5**)\|\| |
| MEASURE |  |  |  |  |  |
| 24 weeks |  |  |  |  |  |
| Tocilizumab + MTX | 35†† | 2 (5.7**) | 2 (5.7**) | 2 / 36†† (5.6**) | 8 / 28†† (28.6**)\|\| |
| Placebo + MTX | 36†† | 3 (8.3**) | 4 (11.1**) | 3 (8.3**) | 7 / 33†† (21.2**)\|\| |
| OPTION |  |  |  |  |  |
| 24 weeks |  |  |  |  |  |
| Tocilizumab + MTX | 179†† | 14 (7.8**) | 18 / 177†† (10.2**) | 10 / 182†† (5.5**) | 44 / 178†† (24.7**)\|\| |
| Placebo + MTX | 173†† | 1 (0.6**) | 1 / 171†† (0.6**) | 1 / 175†† (0.6**) | 1 / 172†† (0.6**)\|\| |
| ROSE |  |  |  |  |  |
| 24 weeks |  |  |  |  |  |
| Tocilizumab + MTX | 161†† | 13 (8.1**) | 15 / 159†† (9.4**) | 12 / 162†† (7.4**) | 53 / 156†† (34.0**)\|\| |
| Placebo + MTX | 68†† | 1 (1.5**) | 1 (1.5**) | 1 (1.5**) | 0 (0^g^)\|\| |
| TOWARD |  |  |  |  |  |
| 24 weeks |  |  |  |  |  |
| Tocilizumab + MTX | 325^h^ | 25 (7.7**) | 26 / 319†† (8.2**) | 24 / 331†† (7.3**) | 95 / 323†† (29.4**)\|\| |
| Placebo + MTX | 166^h^ | 3 (1.8**) | 3 / 162†† (1.9**) | 2 / 169†† (1.2**) | 6 / 167†† (3.6**)\|\| |
| TRACE |  |  |  |  |  |
| 24 weeks |  |  |  |  |  |
| Tocilizumab + MTX | 64^h^ | 4 (6.3**) | 5 (7.8**) | 2 (3.1**) | 20 / 63†† (31.7**)\|\| |
| Placebo + MTX | 37^h^ | 2 (5.4**) | 2 (5.4**) | 2 (5.4**) | 2 / 39†† (5.1**)\|\| |
| AMPLE |  |  |  |  |  |
| 24 weeks |  |  |  |  |  |
| Abatacept + MTX | 305 | 44 (14.4) | 40 / 304 (13.2) | 24 / 304 (7.9) | 93 / 304 (30.6) |
| Adalimumab + MTX | 301 | 52 (17.3) | 50 (16.6) | 34 / 302 (11.3) | 115 / 302 (38.1) |
| 52 weeks |  |  |  |  |  |
| Abatacept + MTX | 277 | 65 (23.5) | 64 / 275 (23.3) | 37 / 275 (13.5) | 119 / 275 (43.3) |
| Adalimumab + MTX | 268 | 64 (23.9) | 66 / 267 (24.7) | 42 (15.7) | 112 (41.8) |
| 104 weeks |  |  |  |  |  |
| Abatacept + MTX | 250 | 80 (32.0) | 78 (31.2) | 52 / 251 (20.7) | 127 / 251 (50.6) |
| Adalimumab + MTX | 244 | 74 (30.3) | 79 / 243 (32.5) | 50 (20.5) | 130 (53.3) |
| EXXELERATE |  |  |  |  |  |
| 24 weeks |  |  |  |  |  |
| Certolizumab pegol + MTX | 418‡‡ | 88 (21.1)§§ | 80 (19.1)§§ | 63 (15.1)§§ | 116 (27.8)§§ |
| Adalimumab + MTX | 418‡‡ | 80 (19.1)§§ | 76 (18.2)§§ | 66 (15.8)§§ | 106 (25.4)§§ |
| 52 weeks |  |  |  |  |  |
| Certolizumab pegol + MTX | 418‡‡ | 101 (24.2)§§ | 95 (22.7)§§ | 75 (17.9)§§ | 111 (26.6)§§ |
| Adalimumab + MTX | 418‡‡ | 95 (22.7)§§ | 87 (20.8)§§ | 69 (16.5)§§ | 116 (27.8)§§ |
| 104 weeks |  |  |  |  |  |
| Certolizumab pegol + MTX | 418‡‡ | 103 (24.6)§§ | 94 (22.5)§§ | 90 (21.5)§§ | 106 (25.4)§§ |
| Adalimumab + MTX | 418‡‡ | 95 (22.7)§§ | 88 (21.1)§§ | 85 (20.3)§§ | 98 (23.4)§§ |
| RA-BEAM |  |  |  |  |  |
| 52 weeks |  |  |  |  |  |
| Baricitinib + MTX | 487 | 105 (21.6) | 110 (22.6) | 76 (15.6) | 193 (39.6) |
| Adalimumab + MTX | 330 | 58 (17.6) | 59 (17.9) | 43 (13.0) | 129 (39.1) |
| FINCH 1 |  |  |  |  |  |
| 52 weeks |  |  |  |  |  |
| Filgotinib + MTX | 475 | 140 (29.5) | 141 (29.7) | 107 (22.5) | 256 (53.9) |
| Adalimumab + MTX | 325 | 74 (22.8) | 78 (24.0) | 55 (16.9) | 150 (46.2) |
| SELECT COMPARE |  |  |  |  |  |
| 26 weeks |  |  |  |  |  |
| Upadacitinib + MTX | 651 | 150 (23.0) | 158 (24.3) | 117 (18.0) | 266 (40.9) |
| Adalimumab + MTX | 327 | 45 (13.8) | 45 (13.8) | 32 (9.8) | 88 (26.9) |
| CDAI=clinical disease activity index; CRP=C-reactive protein; DAS 28=disease activity score 28; DMARD=disease-modifying antirheumatic drug; ESR=erythrocyte sedimentation rate; MTX=methotrexate; N=number of patients included in the analysis; n=number of patients with event; SDAI=simplified disease activity index. *Number of patients included in the analysis. If the number differed between the composite measures, the numbers are mentioned separately as n / N. † ≤ 1 swollen joint, ≤ 1 tender joint, C-reactive protein ≤ 1 mg/dl and global assessment of disease activity by the patient ≤ 1 on a scale from 0 to 10. ‡ Calculated from percentages. § Remission defined as CDAI ≤ 2.8 at week 20 and 24. \|\| DAS 28 [ESR]. ¶ Sum of patients assessed and patients not considered after therapy adjustment due to lack of efficacy (number of patients with therapy adjustment due to lack of efficacy estimated approximately for patients after MTX failure based on the complete study population). ** Calculated (n / N). †† Sum of patients assessed and patients not considered after therapy adjustment. ‡‡ Sum of separate values in study documents for patients with and without therapy adjustment. §§ not considering patients in remission after therapy adjustment. | | | | | |

Supplement Table 14: Results on low disease activity available for the DAS 28 < 3.2. SDAI ≤ 11 or CDAI ≤ 10 from single studies investigating patients after MTX failure

| Study  Analysis time point  Treatment arm | N* | | CDAI ≤ 10  n / N (%) | | | | | SDAI ≤ 11 n / N (%) | | | | DAS 28 [CRP] < 3.2  n / N (%) | | | |
| --- | --- | --- | --- | --- | --- | --- | --- | --- | --- | --- | --- | --- | --- | --- | --- |
| AIM |  | |  | | | | |  | | | |  | | | |
| 24 weeks |  | |  | | | | |  | | | |  | | | |
| Abatacept + MTX | 418 | | 148 / 420 (35.2) | | | | | 140 (33.5) | | | | 126 (30.1) | | | |
| Placebo + MTX | 211 | | 34 (16.1) | | | | | 24 (11.4) | | | | 21 (10.0) | | | |
| 52 weeks |  | |  | | | | |  | | | |  | | | |
| Abatacept + MTX | 424 | | 201 (47.4) | | | | | 196 (46.2) | | | | 180 (42.5) | | | |
| Placebo + MTX | 212 | | 32 (15.1) | | | | | 28 (13.2) | | | | 21 (9.9) | | | |
| ATTEST |  | |  | | | | |  | | | |  | | | |
| 24 weeks |  | |  | | | | |  | | | |  | | | |
| Abatacept + MTX | 155 | | 63 / 154 (40.9) | | | | | 58/ 154 (37.7) | | | | 52 (33.5) | | | |
| Placebo + MTX | 109 | | 23 (21.1) | | | | | 22 (20.2) | | | | 20 (18.3) | | | |
| IM101071 |  | |  | | | | |  | | | |  | | | |
| 24 weeks |  | |  | | | | |  | | | |  | | | |
| Abatacept + MTX | 47 | | 20 (42.6) | | | | | 22 (46.8) | | | | 23 (48.9) | | | |
| Placebo + MTX | 41 | | 7 (17.1) | | | | | 7 (17.1) | | | | 5 (12.2) | | | |
| IM101100 |  | |  | | | | |  | | | |  | | | |
| 24 weeks |  | |  | | | | |  | | | |  | | | |
| Abatacept + MTX | 115 | | 33 (28.7) | | | | | 32 (27.8) | | | | 29 (25.2) | | | |
| Placebo + MTX | 119 | | 13 (10.9) | | | | | 13 (10.9) | | | | 10 (8.4) | | | |
| 52 weeks |  | |  | | | | |  | | | |  | | | |
| Abatacept + MTX | 115 | | 43 (37.4) | | | | | 40 (34.8) | | | | 36 (31.3) | | | |
| Placebo + MTX | 119 | | 19 (16.0) | | | | | 15 (12.6) | | | | 9 (7.6) | | | |
| IM101124 |  | |  | | | | |  | | | |  | | | |
| 24 weeks |  | |  | | | | |  | | | |  | | | |
| Abatacept + MTX | 55 | | 16 (29.1) | | | | | 19 (34.5) | | | | 19 (34.5) | | | |
| Placebo + MTX | 57 | | 10 (17.5) | | | | | 8 (14.0) | | | | 7 (12.3) | | | |
| ARMADA |  | |  | | | | |  | | | |  | |  |  |
| 24 weeks |  | |  | | | | |  | | | |  | |  |  |
| Adalimumab + MTX | 63 | | 30 (47.6) | | | | | 31 (49.2) | | | | 33 (52.4) | |  |  |
| Placebo + MTX 20 mg | 60 | | 4 (6.7) | | | | | 4 (6.7) | | | | 4 (6.7) | |  |  |
| August II |  | |  | | | | |  | | | |  | |  |  |
| 26 weeks |  | |  | | | | |  | | | |  | |  |  |
| Adalimumab + MTX | 79 | | 30 (38.0) | | | | | 32 (40.5) | | | | 20 (25.3) | |  |  |
| Placebo + MTX | 76 | | 12 (15.8) | | | | | 13 (17.1) | | | | 11 (14.5) | |  |  |
| DE019 |  | |  | | | | |  | | | |  | |  |  |
| 24 weeks |  | |  | | | | |  | | | |  | |  |  |
| Adalimumab + MTX | 207 | | 73 (35.3) | | | | | 78 (37.7) | | | | 73 (35.3) | |  |  |
| Placebo + MTX | 200 | | 26 (13.0) | | | | | 23 (11.5) | | | | 19 (9.5) | |  |  |
| 52 weeks |  | |  | | | | |  | | | |  | |  |  |
| Adalimumab + MTX | 207 | | 91 (44.0) | | | | | 92 (44.4) | | | | 84 (40.6) | |  |  |
| Placebo + MTX | 200 | | 24 (12.0) | | | | | 23 (11.5) | | | | 25 (12.5) | |  |  |
| M02-556 |  | |  | | | | |  | | | |  | |  |  |
| 24 weeks |  | |  | | | | |  | | | |  | |  |  |
| Adalimumab + MTX | 65 | | 29 (44.6) | | | | | 29 (44.6) | | | | 35 (53.8) | |  |  |
| Placebo + MTX | 63 | | 9 (14.3) | | | | | 11 (17.5) | | | | 9 (14.3) | |  |  |
| ORAL STANDARD |  | |  | | | | |  | | | |  | |  |  |
| 26 weeks (phase I) |  | |  | | | | |  | | | |  | |  |  |
| Adalimumab + MTX | 178 | | 47 (26.4) | | | | | 51 (28.7) | | | | 54 (30.3) | |  |  |
| Placebo + MTX | 92 | | 12 (13.0) | | | | | 13 / 90 (14.4) | | | | 11 / 90 (12.2) | |  |  |
| RA-BEAM |  | |  | | | | |  | | | |  | |  |  |
| 24 weeks |  | |  | | | | |  | | | |  | |  |  |
| Adalimumab + MTX | 330 | | 157 (47.6) | | | | | 160 (48.5) | | | | 158 (47.9) | |  |  |
| Placebo + MTX | 488 | | 96 (19.7) | | | | | 96 (19.7) | | | | 93 (19.1) | |  |  |
| STAR |  | |  | | | | |  | | | |  | |  |  |
| 24 weeks |  | |  | | | | |  | | | |  | |  |  |
| Adalimumab + MTX | 178 | | 51 (28.7) | | | | | 54 (30.3) | | | | 49 (27.5) | |  |  |
| Placebo + MTX | 199 | | 32 (16.1) | | | | | 34 (17.1) | | | | 32 (16.1) | |  |  |
| 990145 (part A) | |  | |  | | | | |  |  | | | | | |
| 24 weeks | |  | |  | | | | |  |  | | | | | |
| Anakinra + MTX | | 449 | | 55 (12.2) | | | | | 56 (12.5) | 72 (16.0) | | | | | |
| Placebo + MTX | | 450 | | 37 (8.2) | | | | | 35 (7.8) | 37 (8.2) | | | | | |
| 52 weeks | |  | |  | | | | |  |  | | | | | |
| Anakinra + MTX | | 449 | | 45 (10.0) | | | | | 46 (10.2) | 63 (14.0) | | | | | |
| Placebo + MTX | | 450 | | 26 (5.8) | | | | | 27 (6.0) | 30 (6.7) | | | | | |
| 20000198 | |  | |  | | | | |  |  | | | | | |
| 24 weeks | |  | |  | | | | |  |  | | | | | |
| Anakinra + MTX | | 68 | | 10 (14.7) | | | | | 10 (14.7) | 9 (13.2) | | | | | |
| Placebo + MTX | | 68 | | 8 (11.8) | | | | | 6 (8.8) | 4 (5.9) | | | | | |
| CERTAIN | |  | |  | | | | |  |  | | | | | |
| 24 weeks | |  | |  | | | | |  |  | | | | | |
| Certolizumab pegol + DMARD | | 92 | | 58 (63.0) | | | | | 60 (65.2) | 39 (42.4)† | | | | | |
| Placebo + DMARD | | 91 | | 27 (29.7) | | | | | 29 (31.9) | 15 (16.5)† | | | | | |
| RAPID 1 (phase I) | |  | |  | | | | |  |  | | | | | |
| 24 weeks | |  | |  | | | | |  |  | | | | | |
| Certolizumab pegol + MTX | | 391 | | 143 / 392 (36.5) | | | | | 141 / 392 (36.0) | 85 (21.7)† | | | | | |
| Placebo + MTX | | 196 | | 11 (5.6) | | | | | 11 (5.6) | 6 (3.1)† | | | | | |
| 52 weeks | |  | |  | | | | |  |  | | | | | |
| Certolizumab pegol + MTX | | 391 | | 169 / 392 (43.1) | | | | | 172 / 392 (43.9) | 114 (29.2)† | | | | | |
| Placebo + MTX | | 196 | | 14 (7.1) | | | | | 15 (7.7) | 5 (2.6)† | | | | | |
| RAPID 2 | |  | |  | | | | |  |  | | | |  |  |
| 24 weeks | |  | |  | | | | |  |  | | | |  |  |
| Certolizumab pegol + MTX | | 245 | | 63 / 245 (25.7) | | | | | 68 / 245 (27.8) | 41 (16.7)† | | | |  |  |
| Placebo + MTX | | 125 | | 3 / 126 (2.4) | | | | | 3 / 126 (2.4) | 2 (1.6)† | | | |  |  |
| RA0025 |  | |  | | |  | | | |  | | | |  |  |
| 24 weeks |  | |  | | |  | | | |  | | | |  |  |
| Certolizumab pegol + MTX | 70 | | 25 (35.7) | | | 24 (34.3) | | | | 11 (15.7)† | | | |  |  |
| Placebo + MTX | 33 | | 5 (15.2) | | | 5 (15.2) | | | | 0 (0)† | | | |  |  |
| TEMPO |  | |  | |  | | | | |  | | | |  |  |
| 24 weeks |  | |  | |  | | | | |  | | | |  |  |
| Etanercept + MTX | 103 | | 46 (44.7) | | 44 (42.7) | | | | | 45 (43.7) | | | |  |  |
| Placebo + MTX | 97 | | 26 (26.8) | | 24 (24.7) | | | | | 28 (28.9) | | | |  |  |
| 52 weeks |  | |  | |  | | | | |  | | | |  |  |
| Etanercept + MTX | 103 | | 55 (53.4) | | 51 (49.5) | | | | | 53 (51.5) | | | |  |  |
| Placebo + MTX | 97 | | 36 (37.1) | | 32 (33.0) | | | | | 30 (30.9) | | | |  |  |
| 164 weeks |  | |  | |  | | | | |  | | | |  |  |
| Etanercept + MTX | 103 | | 68 (66.0) | | 66 (64.1) | | | | | 62 (60.2) | | | |  |  |
| Placebo + MTX | 97 | | 33 (34.0) | | 33 (34.0) | | | | | 31 (32.0) | | | |  |  |
| 16.0014 |  | |  | |  | | | | |  | | | |  |  |
| 24 weeks |  | |  | |  | | | | |  | | | |  |  |
| Etanercept + MTX | 59 | | 25 (42.4) | | 25 (42.4) | | | | | 35 (59.3) | | | |  |  |
| Placebo + MTX | 30 | | 4 (13.3) | | 4 (13.3) | | | | | 6 (20.0) | | | |  |  |
| C0524T28 |  | |  | |  | | | | |  | | | |  |  |
| 24 weeks |  | |  | |  | | | | |  | | | |  |  |
| Golimumab + MTX | 132 | | 32 (24.2) | | 35 (26.5) | | | | | 41 (31.1) | | | |  |  |
| Placebo + MTX | 132 | | 12 (9.1) | | 11 (8.3) | | | | | 15 (11.4) | | | |  |  |
| GO-FORTH |  | |  | | |  | | | | |  | | |  |  |
| 24 weeks |  | |  | | |  | | | | |  | | |  |  |
| Golimumab + MTX | 81 | | 51 (63.0) | | | 54 (66.7) | | | | | 57 (70.4) | | |  |  |
| Placebo + MTX | 84 | | 27 (32.1) | | | 31 (36.9) | | | | | 29 (34.5) | | |  |  |
| GO-FORWARD |  | |  | | | |  | | | |  | | |  |  |
| 24 weeks |  | |  | | | |  | | | |  | | |  |  |
| Golimumab + MTX | 88 | | 38 (43.2) | | | | 41 (46.6) | | | | 34 (38.6) | | |  |  |
| Placebo + MTX | 127 | | 24 (18.9) | | | | 24 (18.9) | | | | 23 (18.1) | | |  |  |
| ATTRACT |  | |  | | | |  | | | |  | | |  |  |
| 30 weeks |  | |  | | | |  | | | |  | | |  |  |
| Infliximab + MTX | 84 | | 25 (29.8) | | | | 24 / 83 (28.9) | | | | 23 (27.4) | | |  |  |
| Placebo + MTX | 87 | | 6 (6.9) | | | | 5 / 86 (5.8) | | | | 8 (9.2) | | |  |  |
| 54 weeks |  | |  | | | |  | | | |  | | |  |  |
| Infliximab + MTX | 84 | | 19 / 82 (23.2) | | | | 18 / 82 (22.0) | | | | 19 (22.6) | | |  |  |
| Placebo + MTX | 88 | | 8 (9.1) | | | | 6 (6.8) | | | | 9 (10.2) | | |  |  |
| LITHE |  | |  | | | |  | | | |  | | |  |  |
| 24 weeks |  | |  | | | |  | | | |  | | |  |  |
| Tocilizumab + MTX | 308‡ | | 111 (36.0§) | | | | 113 / 306‡ (36.9§) | | | | 140 / 321‡ (43.6§)† | | |  |  |
| Placebo + MTX | 313‡ | | 46 (14.7§) | | | | 46 / 310‡ (14.8§) | | | | 22 / 324‡ (6.8§)† | | |  |  |
| 52 weeks |  | |  | | | |  | | | |  | | |  |  |
| Tocilizumab + MTX | 301‡ | | 124 (41.2§) | | | | 135 / 297‡ (45.5§) | | | | 154 (51.2§)† | | |  |  |
| Placebo + MTX | 312‡ | | 50 (16.0§) | | | | 52 / 311‡ (16.7§) | | | | 27 / 314‡ (8.6§)† | | |  |  |
| MEASURE |  | |  | | | |  | | | |  | | |  |  |
| 24 weeks |  | |  | | | |  | | | |  | | |  |  |
| Tocilizumab + MTX | 35\|\| | | 10 (28.6§)‡ | | | | 11 (31.4§)‡ | | | | 12 / 28\|\| (42.9§)†‡ | | |  |  |
| Placebo + MTX | 36\|\| | | 8 (22.2§)‡ | | | | 9 (25§)‡ | | | | 9 / 33\|\| (27.3§)†‡ | | |  |  |
| OPTION |  | |  | | | |  | | | |  | | |  |  |
| 24 weeks |  | |  | | | |  | | | |  | | |  |  |
| Tocilizumab + MTX | 179\|\| | | 69 (38.5§) | | | | 75 / 177\|\| (42.4§) | | | | 75 / 178\|\| (42.1§)† | | |  |  |
| Placebo + MTX | 173\|\| | | 21 (12.1§) | | | | 20 / 171\|\| (11.7§) | | | | 6 / 172\|\| (3.5§)† | | |  |  |
| ROSE |  | |  | | | |  | | | |  | |  |  |  |
| 24 weeks |  | |  | | | |  | | | |  | |  |  |  |
| Tocilizumab + MTX | 161\|\| | | 56 (34.8§) | | | | 60 / 159\|\| (37.7§) | | | | 68 / 156\|\| (43.6§)† | |  |  |  |
| Placebo + MTX | 68\|\| | | 8 (11.8§) | | | | 7 (10.3§) | | | | 6 (8.8§)† | |  |  |  |
| TOWARD |  | |  | | | |  | | | |  | |  |  |  |
| 24 weeks |  | |  | | | |  | | | |  | |  |  |  |
| Tocilizumab + MTX | 325\|\| | | 109\|\| (33.5§) | | | | 114 / 319\|\| (35.7§) | | | | 151/ 323\|\| (46.7§)† | |  |  |  |
| Placebo + MTX | 166\|\| | | 21 (12.7§) | | | | 21 / 162\|\| (13.0§) | | | | 9 / 167\|\| (5.4§)† | |  |  |  |
| TRACE |  | |  | | | |  | | | |  | |  |  |  |
| 24 weeks |  | |  | | | |  | | | |  | |  |  |  |
| Tocilizumab + MTX | 64\|\| | | 21 (32.8§) | | | | 23 (35.9§) | | | | 28 / 63\|\| (44.4§)† | |  |  |  |
| Placebo + MTX | 37\|\| | | 3 (8.1§) | | | | 3 (8.1§) | | | | 2 / 39\|\| (5.1§)† | |  |  |  |
| AMPLE |  | |  | | | |  | | | |  | |  |  |  |
| 24 weeks |  | |  | | | |  | | | |  | |  |  |  |
| Abatacept + MTX | 304 | | 148 / 305 (48.5) | | | | 151 / 304 (49.7) | | | | 147 (48.4) | |  |  |  |
| Adalimumab + MTX | 302 | | 153 / 301 (50.8) | | | | 158 / 301 (52.5) | | | | 156 (51.7) | |  |  |  |
| 52 weeks |  | |  | | | |  | | | |  | |  |  |  |
| Abatacept + MTX | 275 | | 169 / 277 (61.0) | | | | 171 / 275 (62.2) | | | | 163 (59.3) | |  |  |  |
| Adalimumab + MTX | 268 | | 165 / 268 (61.6) | | | | 169 / 267 (63.3) | | | | 164 (61.2) | |  |  |  |
| 104 weeks |  | |  | | | |  | | | |  | |  |  |  |
| Abatacept + MTX | 251 | | 164 / 250 (65.6) | | | | 163 / 250 (65.2) | | | | 164 (65.3) | |  |  |  |
| Adalimumab + MTX | 244 | | 165 / 244 (67.6) | | | | 168 / 243 (69.1) | | | | 166 (68.0) | |  |  |  |
| EXXELERATE |  | |  | | | |  | | | |  | |  |  |  |
| 24 weeks |  | |  | | | |  | | | |  | |  |  |  |
| Certolizumab Pegol + MTX | 418¶ | | 236 (56.5)** | | | | 230 (55.0)** | | | | 225 (53.8)** | |  |  |  |
| Adalimumab + MTX | 418¶ | | 225 (53.8)** | | | | 227 (54.3)** | | | | 218 (52.2)** | |  |  |  |
| 52 weeks |  | |  | | | |  | | | |  | |  |  |  |
| Certolizumab Pegol + MTX | 418¶ | | 232 (55.5)** | | | | 230 (55.0)** | | | | 220 (52.6)** | |  |  |  |
| Adalimumab + MTX | 418¶ | | 237 (56.7)** | | | | 231 (55.3)** | | | | 226 (54.1)** | |  |  |  |
| 104 weeks |  | |  | | | |  | | | |  | |  |  |  |
| Certolizumab Pegol + MTX | 418¶ | | 198 (47.4)** | | | | 193 (46.2)** | | | | 193 (46.2)** | |  |  |  |
| Adalimumab + MTX | 418¶ | | 203 (48.6)** | | | | 202 (48.3)** | | | | 197 (47.1)** | |  |  |  |
| RA-BEAM |  | |  | | | |  | | | |  | |  |  |  |
| 52 weeks |  | |  | | | |  | | | |  | |  |  |  |
| Baricitinib + MTX | 487 | | 277 (56,9) | | | | 278 (57.1) | | | | 271 (55.6) | |  |  |  |
| Adalimumab + MTX | 330 | | 163 (49.4) | | | | 163 (49.4) | | | | 159 (48.2) | |  |  |  |
| FINCH 1 |  | |  | | | |  | | | |  | |  |  |  |
| 52 weeks |  | |  | | | |  | | | |  | |  |  |  |
| Filgotinib + MTX | 475 | | 318 (66.9) | | | | 320 (67.4) | | | | 313 (65.9) | |  |  |  |
| Adalimumab + MTX | 325 | | 199 (61.2) | | | | 195 (60.0) | | | | 191 (58.8) | |  |  |  |
| SELECT COMPARE |  | |  | | | |  | | | |  | |  |  |  |
| 26 weeks |  | |  | | | |  | | | |  | |  |  |  |
| Upadacitinib + MTX | 651 | | 343 (52.7) | | | | 351 (53.9) | | | | 356 (54.7) | |  |  |  |
| Adalimumab + MTX | 327 | | 125 (38.2) | | | | 127 (38.8) | | | | 126 (38.5) | |  |  |  |
| CDAI=clinical disease activity index; CRP=C-reactive protein; DAS 28=disease activity score 28; DMARD=disease-modifying antirheumatic drug; ESR=erythrocyte sedimentation rate; MTX=methotrexate; N=number of patients included in the analysis; n=number of patients with event; SDAI=simplified disease activity index. *Number of patients included in the analysis. If the number differed between the composite measures, the numbers are mentioned separately as n / N. †DAS 28 [ESR]. ‡Sum of patients assessed and patients not considered after therapy adjustment due to lack of efficacy (number of patients with therapy adjustment due to lack of efficacy estimated approximately for patients after MTX failure based on the complete study population).§ Calculated (n / N). \|\| Sum of patients assessed and patients not considered after therapy adjustment. ¶ Sum of separate values in study documents for patients with and without therapy adjustment. ** Not considering patients with low disease activity after therapy adjustment. | | | | | | | | | | | | | | |  |

Supplement Table 15: Results on remission available for the DAS 28 < 2.6. SDAI ≤ 3.3. CDAI ≤ 2.8 or Boolean definition from single studies investigating patients with MTX intolerance

| Study  Analysis time point  Treatment arm | N | CDAI ≤ 2.8  n (%) | SDAI ≤ 3.3  n (%) | Boolean definition*  n (%) | DAS 28 [ESR] < 2.6  n (%) |
| --- | --- | --- | --- | --- | --- |
| ADACTA |  |  |  |  |  |
| 24 weeks |  |  |  |  |  |
| Tocilizumab | 63 | 11 (17.5) | 12 (19.0) | 12 (19.0) | 22 (34.9) |
| Adalimumab | 68 | 8 (11.8) | 6 (8.8) | 6 (8.8) | 6 (8.8) |
| MONARCH |  |  |  |  |  |
| 24 weeks |  |  |  |  |  |
| Sarilumab | 87 | 8 (9.2) | 9 (10.3) | 5 (5.7) | 26 (29.9) |
| Adalimumab | 82 | 2 (2.4) | 2 (2.4) | 2 (2.4) | 5 (6.1) |
| CDAI=clinical disease activity index; DAS 28=disease activity score 28; ESR=erythrocyte sedimentation rate; N=number of patients included in the analysis; n=number of patients with event; SDAI=simplified disease activity index. * ≤ 1 swollen joint, ≤ 1 tender joint, C-reactive protein ≤ 1 mg/dl and global assessment of disease activity by the patient ≤ 1 on a scale from 0 to 10. | | | | | |

Supplement Table 16: Results on low disease activity available for the DAS 28 < 3.2. SDAI ≤ 11 or CDAI ≤ 10 from single studies investigating patients with MTX intolerance

| Study  Analysis time point  Treatment arm | N | CDAI ≤ 10  n (%) | SDAI ≤ 11  n (%) | DAS 28 [ESR] ≤ 3.2  n (%) |
| --- | --- | --- | --- | --- |
| ADACTA |  |  |  |  |
| 24 weeks |  |  |  |  |
| Tocilizumab | 63 | 29 (46.0)* | 30 (47.6)† | 29 (46.0) |
| Adalimumab | 68 | 27 (39.7)* | 28 (41.2)† | 18 (26.5) |
| MONARCH |  |  |  |  |
| 24 weeks |  |  |  |  |
| Sarilumab | 87 | 39 (44.8) | 40 (46.0) | 39 (44.8) |
| Adalimumab | 82 | 15 (18.3) | 16 (19.5) | 8 (9.8) |
| CDAI=clinical disease activity index; DAS 28=disease activity score 28; ESR=erythrocyte sedimentation rate; N=number of patients included in the analysis; n=number of patients with event; SDAI=simplified disease activity index. * Calculated from CDAI ≤ 2.8 and 2.8 < CDAI ≤ 10. † Calculated from SDAI ≤ 3.3 and 3.3 < SDAI ≤ 11. | | | | |

Supplement Table 17: Results on remission available for the DAS 28 < 2.6. SDAI ≤ 3.3. CDAI ≤ 2.8 or Boolean definition from single studies investigating patients after biologic failure

| Study  Analysis time point  Treatment arm | N* | | CDAI ≤ 2.8  n / N (%) | | SDAI ≤ 3.3  n / N (%) | Boolean definition†  n / N (%) | | DAS 28 [CRP] < 2.6  n / N (%) |
| --- | --- | --- | --- | --- | --- | --- | --- | --- |
| ATTAIN |  | |  | |  |  | |  |
| 24 weeks |  | |  | |  |  | |  |
| Abatacept + MTX | 169 | | 11 (6.5) | | 9 (5.3) | 7 (4.1) | | 17 (10.1) |
| Placebo + MTX | 85 | | 0 (0) | | 0 (0) | 0 (0) | | 1 / 86 (1.2) |
| IM101071 |  | |  | |  |  | |  |
| 24 weeks |  | |  | |  |  | |  |
| Abatacept + MTX | 14 | | 0 (0) | | 1 (7.1) | 1 (7.1) | | 2 (14.3) |
| Placebo + MTX | 25 | | 0 (0) | | 0 (0) | 0 (0) | | 0 (0) |
| ORAL STANDARD |  | |  | |  |  | |  |
| 24 weeks |  | |  | |  |  | |  |
| Adalimumab + MTX | 19 | | 0 (0) | | 0 (0) | 1 (5.3) | | 2 (10.5) |
| Placebo + MTX | 12 | | 0 (0) | | 0 (0) | 0 (0) | | 1 (8.3) |
| RA0025 |  | |  | |  |  | |  |
| 24 weeks |  | |  | |  |  | |  |
| Certolizumab pegol + MTX | 11 | | 1 (9.1) | | 0 (0) | 0 (0) | | 0 (0)‡ |
| Placebo + MTX | 7 | | 0 (0) | | 0 (0) | 0 (0) | | 0 (0)‡ |
| GO-AFTER |  | |  | |  |  | |  |
| 24 weeks |  | |  | |  |  | |  |
| Golimumab + MTX | 98 | | 12 (12.2) | | 12 (12.2) | 11 (11.2) | | 14 (14.3) |
| Placebo + MTX | 91 | | 2 (2.2) | | 2 (2.2) | 2 (2.2) | | 3 (3.3) |
| DANCER |  |  | |  | | |  |  |
| 24 weeks |  |  | |  | | |  |  |
| Rituximab + MTX | 16 | 0 (0) | | 0 (0) | | | 0 (0) | 0 (0) |
| Placebo + MTX | 19 | 0 (0) | | 0 (0) | | | 0 (0) | 0 (0) |
| REFLEX |  |  | |  | | |  |  |
| 24 weeks |  |  | |  | | |  |  |
| Rituximab + MTX | 298 | 17 (5.7) | | 16 (5.4) | | | 11 / 293 (3.7) | 25 / 293 (8.4)‡ |
| Placebo + MTX | 201 | 1 (0.5) | | 0 (0) | | | 2 / 200 (1) | 2 / 200 (1)‡ |
| MEASURE |  |  | |  | | |  |  |
| 24 weeks |  |  | |  | | |  |  |
| Tocilizumab + MTX | 28§ | 3 (10.7\|\|) | | 4 / 27§ (14.8\|\|) | | | 4 (14.3\|\|) | 9 / 27§ (33.3\|\|)‡ |
| Placebo + MTX | 24§ | 0 (0) | | 0 (0) | | | 0 (0) | 0 (0)‡ |
| OPTION |  |  | |  | | |  |  |
| 24 weeks |  |  | |  | | |  |  |
| Tocilizumab + MTX | 12§ | 2 (16.7\|\|) | | 2 (16.7\|\|) | | | 1 (8.3\|\|) | 3 (25.0\|\|)‡ |
| Placebo + MTX | 16§ | 0 (0\|\|) | | 0 (0\|\|) | | | 0 /17§ (0\|\|) | 0 / 17§ (0\|\|)‡ |
| RADIATE |  |  | |  | | |  |  |
| 24 weeks |  |  | |  | | |  |  |
| Tocilizumab + MTX | 148§ | 8 (5.4\|\|) | | 8 (5.4\|\|) | | | 6 / 143§ (4.2\|\|) | 38 / 143§ (26.6\|\|)‡ |
| Placebo + MTX | 128§ | 1 (0.8\|\|) | | 1 / 127§ (0.8\|\|) | | | 0 (0\|\|) | 1 / 127§ (0.8\|\|)‡ |
| ROSE |  |  | |  | | |  |  |
| 24 weeks |  |  | |  | | |  |  |
| Tocilizumab + MTX | 100§ | 9 (9\|\|) | | 9 (9\|\|) | | | 7 / 102§ (6.9\|\|) | 29 / 99§ (29.3\|\|)‡ |
| Placebo + MTX | 63§ | 0 (0\|\|) | | 0 / 62§ (0\|\|) | | | 0 (0\|\|) | 1 (1.6\|\|)‡ |
| TOWARD |  |  | |  | | |  |  |
| 24 weeks |  |  | |  | | |  |  |
| Tocilizumab + MTX | 91§ | 6 (6.6\|\|) | | 5 / 88§ (5.7\|\|) | | | 6 / 93§ (6.5\|\|) | 21 / 90§ (23.3\|\|)‡ |
| Placebo + MTX | 41§ | 1 (2.4\|\|) | | 1 (2.4\|\|) | | | 1 (2.4\|\|) | 1 (2.4\|\|)‡ |
| TRACE |  |  | |  | | |  |  |
| 24 weeks |  |  | |  | | |  |  |
| Tocilizumab + MTX | 12 | 1 (8.3) | | 1 (8.3) | | | 0 (0) | 7 (58.3)‡ |
| Placebo + MTX | 4 | 0 (0) | | 0 (0) | | | 0 (0) | 0 (0)‡ |
| ACT-FIRST |  |  | |  | | |  |  |
| 24 weeks |  |  | |  | | |  |  |
| Adalimumab + MTX | 33 | 2 (6.1) | | 1 (3.0) | | | 2 (6.1) | 10 (30.3)‡ |
| Tocilizumab + MTX | 36 | 3 (8.3) | | 3 / 35 (8.6) | | | 3 / 35 (8.6) | 13 (36.1)‡ |
| SELECT CHOICE |  |  | |  | | |  |  |
| 24 weeks |  |  | |  | | |  |  |
| Upadacitinib + MTX | 223 | 51 (22.9) | | 52 (23.3) | | | 38 (17.0) | 115 (51.6) |
| Abatacept + MTX | 215 | 34 (15.8) | | 31 (14.4) | | | 25 (11.6) | 71 (33.0) |
| CDAI=clinical disease activity index; CRP=C-reactive protein; DAS 28=disease activity score 28; ESR=erythrocyte sedimentation rate; MTX=methotrexate; N=number of patients included in the analysis; n=number of patients with event; SDAI=simplified disease activity index. *Number of patients included in the analysis. If the number differed between the composite measures, the numbers are mentioned separately as n / N. † ≤ 1 swollen joint, ≤ 1 tender joint, C-reactive protein ≤ 1 mg/dl and global assessment of disease activity by the patient ≤ 1 on a scale from 0 to 10. ‡DAS 28 [ESR] § Sum of patients assessed and patients not considered after therapy adjustment.due to lack of efficacy. \|\| Calculated (n / N). | | | | | | | | |

Supplement Table 18: Results on low disease activity available for the DAS 28 < 3.2. SDAI ≤ 11 or CDAI ≤ 10 from single studies investigating patients after biologic failure

| Study  Analysis time point  Treatment arm | N* | CDAI ≤ 10  n / N (%) | SDAI ≤ 11  n / N (%) | DAS 28 [CRP] < 3.2  n / N (%) |  |
| --- | --- | --- | --- | --- | --- |
| ATTAIN |  |  |  |  |  |
| 24 weeks |  |  |  |  |  |
| Abatacept + MTX | 169 | 33 (19.5) | 32 (18.9) | 28 (16.6) |  |
| Placebo + MTX | 85 | 4 (4.7) | 3 (3.5) | 4 / 86 (4.7) |  |
| IM101071 |  |  |  |  |  |
| 24 weeks |  |  |  |  |  |
| Abatacept + MTX | 14 | 2 (14.3) | 2 (14.3) | 2 (14.3) |  |
| Placebo + MTX | 25 | 0 (0) | 0 (0) | 0 (0) |  |
| ORAL STANDARD |  |  |  |  |  |
| 24 weeks |  |  |  |  |  |
| Adalimumab + MTX | 19 | 5 (26.3) | 5 (26.3) | 4 (21.1) |  |
| Placebo + MTX | 12 | 1 (8.3) | 1 (8.3) | 1 (8.3) |  |
| RA0025 |  |  |  |  |  |
| 24 weeks |  |  |  |  |  |
| Certolizumab pegol + MTX | 11 | 6 (54.5) | 6 (54.5) | 1 (9.1) |  |
| Placebo + MTX | 7 | 1 (14.3) | 1 (14.3) | 0 (0) |  |
| GO-AFTER |  |  |  |  |  |
| 24 weeks |  |  |  |  |  |
| Golimumab + MTX | 98 | 22 (22.4) | 22 (22.4) | 22 (22.4) |  |
| Placebo + MTX | 91 | 9 (9.9) | 9 (9.9) | 7 (7.7) |  |
| DANCER |  |  |  |  |  |
| 24 weeks |  |  |  |  |  |
| Rituximab + MTX | 16 | 3 (18.8) | 3 (18.8) | 1 (6.3) |  |
| Placebo + MTX | 19 | 0 (0) | 0 (0) | 0 (0) |  |
| REFLEX |  |  |  |  |  |
| 24 weeks |  |  |  |  |  |
| Rituximab + MTX | 293 | 68 / 298 (22.8) | 68 / 298 (22.8) | 44 (14.8)† |  |
| Placebo + MTX | 200 | 10 / 201 (5.0) | 10 / 201 (5.0) | 4 (2.0)† |  |
| MEASURE |  |  |  |  |  |
| 24 weeks |  |  |  |  |  |
| Tocilizumab + MTX | 28‡ | 8 (28.6§) | 7 / 27‡ (25.9§) | 10 / 27‡ (37.0§)† |  |
| Placebo + MTX | 24‡ | 0 (0§) | 0 (0§) | 1 (4.2§)† |  |
| OPTION |  |  |  |  |  |
| 24 weeks |  |  |  |  |  |
| Tocilizumab + MTX | 12‡ | 3 (25.0§) | 3 (25.0§) | 3 (25.0§)† |  |
| Placebo + MTX | 16‡ | 0 (0§) | 0 (0§) | 0 / 17‡ (0§)† |  |
| RADIATE |  |  |  |  |  |
| 24 weeks |  |  |  |  |  |
| Tocilizumab + MTX | 148‡ | 48 (32.4§) | 49 (33.1§) | 63 /143‡ (44.1§)† |  |
| Placebo + MTX | 128‡ | 8 (6.3§) | 8 / 127‡ (6.3§) | 3 / 127‡ (2.4§)† |  |
| ROSE |  |  |  |  |  |
| 24 weeks |  |  |  |  |  |
| Tocilizumab + MTX | 100‡ | 33 (33§) | 37 (37§) | 36 / 99‡ (36.4§) |  |
| Placebo + MTX | 63‡ | 7 (11.1§) | 8 / 62‡ (12.9§) | 4 (6.3§) |  |
| TOWARD |  |  |  |  |  |
| 24 weeks |  |  |  |  |  |
| Tocilizumab + MTX | 91‡ | 20 (22.0§) | 19 / 88‡ (21.6§) | 24 / 90‡ (28.2§)† |  |
| Placebo + MTX | 41‡ | 2 (4.9§) | 2 (4.9§) | 1 (2.4§)† |  |
| TRACE |  |  |  |  |  |
| 24 weeks |  |  |  |  |  |
| Tocilizumab + MTX | 12‡ | 5 (41.7§) | 5 (41.7§) | 8 (66.7§)† |  |
| Placebo + MTX | 4‡ | 1 (25.0§) | 1 (25.0§) | 0 (0§)† |  |
| ACT-FIRST |  |  |  |  |  |
| 24 weeks |  |  |  |  |  |
| Adalimumab + MTX | 33 | 14 (42.4) | 9 (27.3) | 10 (30.3)† |  |
| Tocilizumab + MTX | 36 | 17 (47.2) | 17 / 35 (48.6) | 21 (58.3)† |  |
| SELECT CHOICE |  |  |  |  |  |
| 24 weeks |  |  |  |  |  |
| Upadacitinib + MTX | 223 | 137 (61.4) | 140 (62.8) | 148 (66.4) |  |
| Abatacept + MTX | 215 | 115 (53.5) | 115 (53.5) | 106 (49.3) |  |
| CDAI=clinical disease activity index; CRP=C-reactive protein; DAS 28=disease activity score 28; ESR=erythrocyte sedimentation rate; MTX=methotrexate; N=number of patients included in the analysis; n=number of patients with event; SDAI=simplified disease activity index. *Number of patients included in the analysis. If the number differed between the composite measures, the numbers are mentioned separately as n / N. † DAS 28 [ESR] ‡ Sum of patients assessed and patients not considered after therapy adjustment.due to lack of efficacy. § Calculated (n / N). | | | | | |

Supplement Table 19: Results on remission available for the DAS 28 < 2.6. SDAI ≤ 3.3. CDAI ≤ 2.8 or Boolean definition from single studies investigating patients after MTX or biologic failure

| Study  Analysis time point  Treatment arm | N | CDAI ≤ 2.8  n (%) | SDAI ≤ 3.3  n (%) | Boolean definition*  n (%) | DAS 28 [CRP] < 2.6  n (%) |
| --- | --- | --- | --- | --- | --- |
| ORAL STANDARD |  |  |  |  |  |
| 52 weeks |  |  |  |  |  |
| Tofacitinib + MTX | 193 | 28 (14.5) | 28 (14.5) | 20 (10.4) | 54 (28.0) |
| Adalimumab + MTX | 197 | 23 (11.7) | 25 (12.7) | 17 (8.6) | 53 (26.9) |
| ORAL STRATEGY |  |  |  |  |  |
| 52 weeks |  |  |  |  |  |
| Tofacitinib + MTX | 376 | 70 (18.6) | 61 (16.2) | 49 (13.0) | 114 (30.3) |
| Adalimumab + MTX | 386 | 65 (16.8) | 62 (16.1) | 47 (12.2) | 136 (35.2) |
| CDAI=clinical disease activity index; CRP=C-reactive protein; DAS 28=disease activity score 28; MTX=methotrexate; N=number of patients included in the analysis; n=number of patients with event; SDAI=simplified disease activity index. * ≤ 1 swollen joint, ≤ 1 tender joint, C-reactive protein ≤ 1 mg/dl and global assessment of disease activity by the patient ≤ 1 on a scale from 0 to 10. | | | | | |

Supplement Table 20: Results on low disease activity available for the DAS 28 < 3.2. SDAI ≤ 11 or CDAI ≤ 10 from single studies investigating patients after MTX or biologic failure

| Study  Analysis time point  Treatment arm | N | CDAI ≤ 10  n (%) | SDAI ≤ 11  n (%) | DAS 28 [CRP] ≤ 3.2  n (%) |
| --- | --- | --- | --- | --- |
| ORAL STANDARD |  |  |  |  |
| 52 weeks |  |  |  |  |
| Tofacitinib + MTX | 193 | 86 (44.6) | 88 (45.6) | 88 (45.6) |
| Adalimumab + MTX | 197 | 73 (37.1) | 75 (38.1) | 86 (43.7) |
| ORAL STRATEGY |  |  |  |  |
| 52 weeks |  |  |  |  |
| Tofacitinib + MTX | 376 | 188 (50.0) | 187 (49.7) | 175 (46.5) |
| Adalimumab + MTX | 386 | 202 (52.3) | 204 (52.8) | 201 (52.1) |
| CDAI=clinical disease activity index; CRP=C-reactive protein; DAS 28=disease activity score 28; MTX=methotrexate; N=number of patients included in the analysis; n=number of patients with event; SDAI=simplified disease activity index. | | | | |
